# Autophagy in Viral Development and Progression of Cancer

**DOI:** 10.3389/fonc.2021.603224

**Published:** 2021-03-08

**Authors:** Alejandra Suares, María Victoria Medina, Omar Coso

**Affiliations:** ^1^ Departamento de Fisiología y Biología Molecular, Facultad de Ciencias Exactas y Naturales, Universidad de Buenos Aires, Buenos Aires, Argentina; ^2^ Instituto de Fisiología, Biología Molecular y Neurociencias (IFIBYNE), CONICET—Universidad de Buenos Aires, Buenos Aires, Argentina

**Keywords:** autophagy, human diseases, cancer cells, oncoviruses, cell survival, cell death

## Abstract

Autophagy is a complex degradative process by which eukaryotic cells capture cytoplasmic components for subsequent degradation through lysosomal hydrolases. Although this catabolic process can be triggered by a great variety of stimuli, action in cells varies according to cellular context. Autophagy has been previously linked to disease development modulation, including cancer. Autophagy helps suppress cancer cell advancement in tumor transformation early stages, while promoting proliferation and metastasis in advanced settings. Oncoviruses are a particular type of virus that directly contribute to cell transformation and tumor development. Extensive molecular studies have revealed complex ways in which autophagy can suppress or improve oncovirus fitness while still regulating viral replication and determining host cell fate. This review includes recent advances in autophagic cellular function and emphasizes its antagonistic role in cancer cells.

## Introduction

Living organisms survive and are naturally preserved thanks to the combination of complex systems that coordinate to maintain homeostatic balance (1). The immune and endocrine systems represent good examples, as specialized cells and chemical mediators work together with antibodies and hormones to generate a specific response in the body (2). Individuals constantly face tissue damage due to stressful and environmental signals, as well as normal body deterioration and aging consequences (3). This is why organisms need intracellular signaling mechanisms that allow them to protect themselves from damaged cells, either by killing them or inhibiting their spread (4). In this way, organisms are prevented from preserving defective cell lines with potential mutation or error accumulation that may contribute to disease risk (5).

Macroautophagy (autophagy) is a metabolic process of intracellular component autodegradation, such as proteins and organelles, crucial for maintaining metabolism and cellular homeostasis (6). Normal levels of basal autophagy prevent cells from gradually accumulating proteins and damaged organelles that can become toxic to cells over time (7). Identifying the mechanistic components of this process at the cellular and molecular levels has been of great interest to researchers worldwide since the late 1950s (8). The first scientists to study and coin the name this catabolic mechanism believed that autophagy was just a cytoplasmic “*cleaning mechanism*” by which cells remove harmful components that accumulate in the cytoplasm (9). This explains the etymology of the term, which comes from the Greek words “*phagy*,” meaning “*eat*,” and “*auto*,” meaning “*me*.” However, the role of autophagy in cells is now considered to be much broader as well as strongly influenced by the cellular environment. Autophagy modulation is related to human pathophysiology, and its implications affect different medical fields (10). This review summarizes the advances in molecular biology in relation to how this catabolic process helps develop different human diseases, focusing primarily on autophagy’s dual role in health maintenance and tumor progression, with special interest in tumors associated with viral infections.

## Mechanism of Autophagy

**Figure 1:**
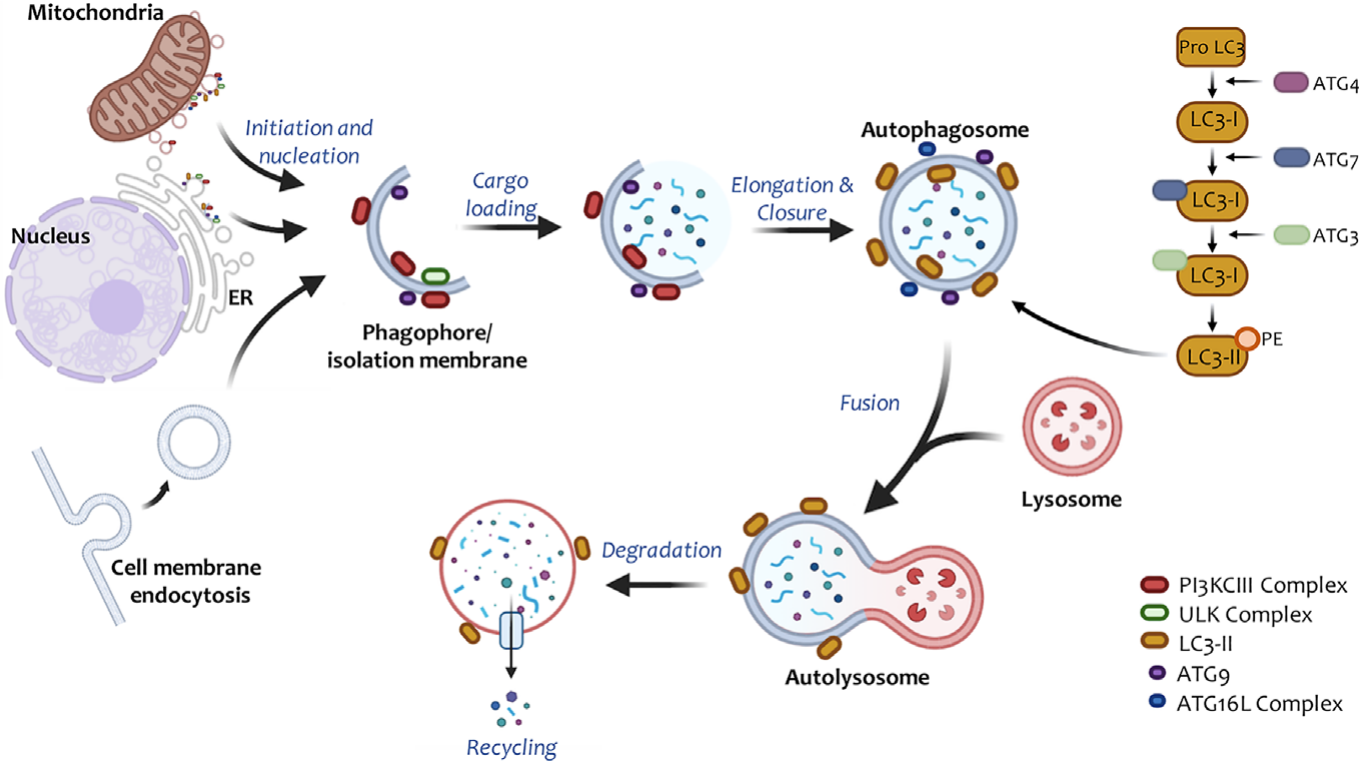
Steps involved in autophagic responses. Autophagy begins with the progressive segregation of cytoplasmic material by double-membrane structures, commonly known as phagophores or isolation membranes. In general, this process is preceded by the inactivation of the PI3K/Akt/mTOR signaling axis. Phagophores are nucleated primarily from the endoplasmic reticulum (ER), but other organelles, such as the Golgi apparatus, plasma membrane, mitochondria, and recycling endosomes have also been shown to participate in this process. The complexes ULK1 and PI3KCIII are involved during phagophore initiation and elongation. Pro-LC3 is free in the cytoplasm and by the action of ATG4 and ATG7, LC3-I is formed. This molecule interacts with the complex ATG16L, ATG3, to later incorporate a phosphatidylethanolamine (PE) molecule into its structure. This results in LC3-II, which binds to autophagosomal membranes and contributes to phagophore elongation as well as closure. Then, these membranous structures seal, and autophagosomes are entirely assembled. Subsequently, autophagosomes fuse with lysosomes to form single membrane structures called autolysosomes, where the degradative process takes place through lysosomal hydrolases. The degradation products of these catabolic reactions reach the cytosol through transporters in the lysosomal membrane and are recycled by bioenergetic circuits.

Up to date, 32 *atg* (autophagy-related genes), involved in regulating different autophagy stages have been identified in mammals. These genes encode numerous proteins (ATG) that regulate the autophagic machinery (11). Autophagy can be divided mechanistically into different stages: 1) initiation and nucleation (molecule recruitment for isolation membrane extension), 2) phagophore elongation and closure (autophagosome), 3) fusion with lysosomes (autolysosomes), 4) degradation, and 5) cytoplasmic material recycling (12) (**Figure 1**). Mammalian cells induce the autophagic machinery in response to various cellular stimuli, such as prolonged starvation (13), decreased glucose levels (14), hypoxia (15–17), increased levels of reactive oxygen species (ROS) (18, 19), and ER (endoplasmic reticulum) stress (20, 21), among others (22).

AMPK protein (AMP-activated kinase) is the main inducer of the autophagic machinery thus reducing intracellular ATP levels. Recent studies have revealed AMPK’s fundamental role in autophagosome maturation and its fusion with lysosomes (23). Similarly, mTOR (mammalian target of rapamycin) activity is regulated by amino acid and glucose levels in mammalian cells (24), mTOR being their main autophagy inhibitor (25). Specifically, mTORC1 (mTOR complex 1) detects the cell’s nutritional status and activates various signaling pathways to regulate cell fate (26). mTORC1 detects and responds to fluctuations of intra and extracellular nutrient levels, mainly amino acids and oxygen, as well as various growth factors (27). A complex dynamic between mTOR and AMPK enables coordinated regulation of signaling pathways in response to cellular environment changes (28).

High-nutrient levels promote mTORC1 inactivation and the induction of anabolic pathways involving protein, lipid, and nucleotide synthesis through S6K (ribosomal protein kinase S6) and 4E-BP1 (initiation factor of the eukaryotic translation 4E - binding protein 1) phosphorylation (29). At the same time, catabolic cellular programs are suppressed as ULK1 is inhibited (Unc-51-like kinase 1, mammalian homolog of *atg1*), thus leading -in turn- to autophagy inhibition (30). Multiprotein complex ULK1 (ULK1, Beclin-1 (BECN1), and PI3KCIII (phosphatidylinositol-3 kinase class III)) mainly regulates the autophagic mechanism initiation process (31). Once active, the complex is recruited to the isolation membrane, where it contributes to PIP3 (phosphatidylinositol 3-phosphate) formation and Akt activation (32). These cellular events dampen TSC 1/2 (tuberous sclerosis protein 1 and 2)inhibitory effect, a protein heterodimer homologous to RHEB (RAS enriched in brain protein) (33, 34). Akt can also be inhibited by mTORC2, further contributing to autophagy inhibition (35). PTEN (phosphatidylinositol-3,4,5-trisphosphate 3-phosphatase) can hinder PIP3 formation, by activating the PI3K/Akt/mTOR pathway (36).

How the isolation membrane resulting in the phagophore forms still remains unclear. However, this membrane has been reported to derive from the plasma membrane, the endoplasmic reticulum (37), the Golgi apparatus (38), the ER-Golgi intermediate compartment (39), and the mitochondria (40). In these membranes, nucleation occurs (41) (**Figure 1**). All proteins involved in pagophore elongation, maturation, and closure are recruited through this process (42). The phagophore incorporates and degrades cytoplasmic material during extension and then it closes up, forming double-membrane vesicular structures called autophagosomes (35).

The action of PI3KCIII multiprotein complex (VPS34 (vacuolar proteins sorting 34), BECN1, p150 (ortholog of mammals of VPS15), mAtg14) in the protein recruitment process to the isolation membrane is fundamental (12). At this point, achieving active recycling is essential, a process involving the intervention of the ATG9 protein anchored in the membranes (31). Some Bcl-2 (B-cell lymphoma 2) family members, such as Bcl-2 and Bcl-xL (B-cell lymphoma extra-large), are known inhibitors of programmed cell death but can also inhibit autophagy through their interaction with BECN1 (43–45). Such interaction does not allow BECN1 to interact with VPS34 (46–48).

After nucleation, the ATG16L complex (ATG12, ATG5, ATG16L1) is recruited into the membrane, where it contributes to LC3 (light chain microtubule-associated protein-1 or MAP1LC3B) (49), GATE-16 (Golgi-associated ATPase enhancer of 16 kDa), and GABARAP (aminobutyric protein associated with the γ-acid receptor) lipidation (11, 50). These three groups belong to the ATG8 protein family, highly conserved across the evolutionary scale (51). Several proteins are anchored to the phagophore membrane, which is shed and returned to the cytoplasm before closure. Meanwhile, LC3 remains attached to the autophagosomal membranes throughout the process, making it a useful autophagosome marker (52) (**Figure 1**).

Several cellular receptors are involved in the selective recognition and recruitment of the cytoplasmic material that is later degraded in autolysosomes. The best-characterized autophagy receptor to date is p62 (Sequestosome 1 or SQSTM1), a molecular adapter with a ubiquitin-binding site and another for LC3 (35). p62 can also promote inflammatory gene expression through NF-kB (nuclear factor kB) regulation, activated when binding to TRAF6 (tumor necrosis factor receptor-associated factor 6) (53). Furthermore, p62 has been shown to activate an antioxidant response by sequestering Keap-1 (Kelch-like ECH-associated protein-1) through an Nrf2-dependent (erythroid-derived nuclear factor 2) mechanism (54) as well as to activate mTORC1 and regulate c-Myc (55).

After formation, autophagosomes relocalize to the perinuclear region through microtubules, where they fuse with lysosomes to form single membrane vesicles called autolysosomes (56), a complex process requiring anchoring factors and about which very little is known so far (57). SNARE proteins (soluble NSF binding protein) take part in the recognition and fusion of these structures. Studies in mice have revealed the importance of a SNARE complex [VTI1B, syntaxin 8, syntaxin 7, and VAMP-8 (vesicle-associated membrane protein 8)] in late fusion with the lysosome (Vadim Atlashkin2003). UVRAG (a gene associated with ultraviolet radiation) can activate GTPase RAB7 to promote fusion (58). Syntaxin 17, located in mature autophagosomes, can also regulate this mechanism (59). Successful binding to lysosomes is necessary for complete autophagy, as lysosomes provide the lytic enzymes needed for the degradation of cytoplasmic components in vesicles (60).

## Functions of Autophagy

Historically, autophagy was considered to be a mechanism benefitting cell survival, as it recycles damaged and potentially toxic cytoplasmic components to increase vitality in cells subjected to stressful conditions, such as nutrient deprivation (61). Depending on cellular context, autophagy is selective or non-selective (massive autophagy). The former shows high specificity in degradation load selection and delivery, while in the latter, cytoplasmic particles are incorporated randomly (62). Selective autophagy is defined by the cytoplasmic material digested in mitophagy (affects mitochondria) (63), pexophagy (peroxisomes) (64), reticulophagy (endoplasmic reticulum and ribosomes) (65), nucleophagy (nucleus) (66), lipophagy (fat cells) (67), xenophagy (involves pathogens and other non-host entities) (68), and aggrephagy (damaged protein aggregates) (69).

With the advancement of knowledge, autophagy has been found not only to promote cell survival, but also to be induced in dying cells (70). The role of autophagy in death is, even today, a critical controversial point among researchers. While some scientists consider autophagy to been an independent death mechanism (autosis) (71), others argue that activation in dying cells occurs as a failure to rescue the cells from the stressful stimuli leading them to death in the first place (72, 73). In light of the growing number of physiological functions related to the autophagic mechanism, connections with numerous human pathologies have also been strengthened (74).

## The Role of Autophagy in Cancer

For cell transformation and tumor development to happen, several basic cellular alterations -referred to as *the hallmarks of cancer*- must occur (75, 76). Increasing evidence suggests a link between autophagy and cancer (77).

**Figure 2:**
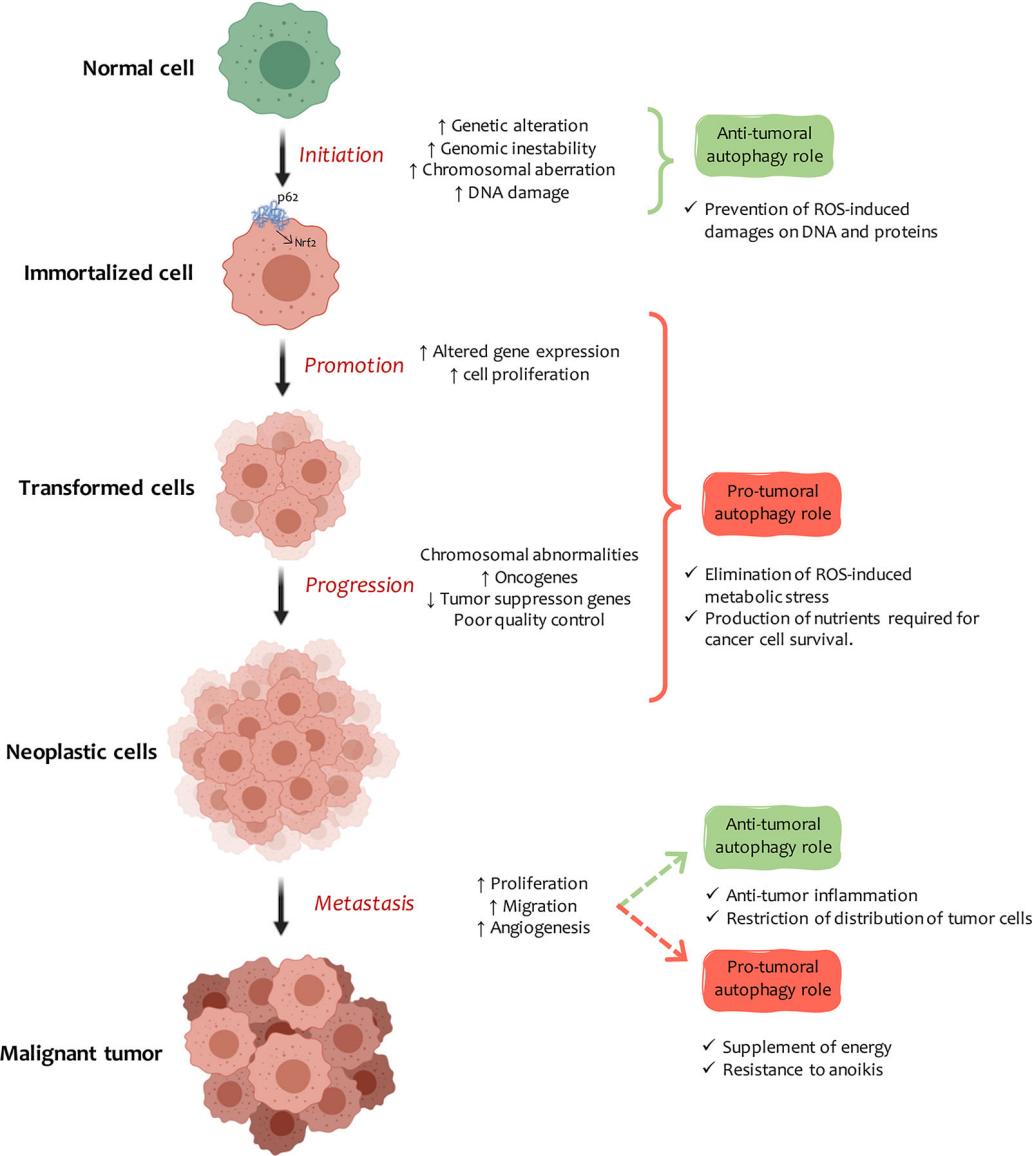
Autophagy and cancer. Autophagy plays a dual role in the development of cancer, the nature of which depends on the tissue, stage, and type of tumor. In carcinogenesis early stage, autophagy induction protects cells from DNA and protein damage due to the indiscriminate increase in ROS and cell-toxic components. Once cells are immortalized, autophagy plays an essential role in promoting tumors. Autophagy induction can be modified, or at least some of the steps involved in the mechanism can be changed. The excessive accumulation of autophagic vesicles leads to p62 molecule accumulation and intracellular signaling activation, which leads to Nrf2 transformation, inflammation, and cell necrosis. In tumor progression, autophagy provides high-energy nutrients to actively dividing transformed cells. When transformed cells metastasize, autophagy can inhibit the process by promoting anti-tumor inflammatory responses or by restricting the expansion of dormant metastatic tumor cells. On the contrary, it supports metastasis by improving cell fitness against a stressful microenvironment (anoikis).

However, establishing the role of autophagy in cancer has proved problematic as it can both contribute to tumor promotion and inhibition, depending on cellular context and disease stage (78). In the early stages of tumor transformation, autophagy can be activated to help cells mitigate mutations and damage their various components. But, once the transformation is complete, tumor cells can make use of the autophagic machinery to meet the high metabolic requirements of these uncontrolled dividing cells (79) (**Figure 2**).

### Autophagy as a Tumor-Suppressing Mechanism

The most relevant findings related to autophagy and its role as a tumor suppressor come from studies on BECN1 (80). Mice with depletion of an allele for this gene showed a higher predisposition to different neoplasm spontaneous development (81). BECN1 has a BH3 domain, so it is not surprising that it can interact with various members of the Bcl-2 protein family and homologous viral proteins (82). Through these interactions, BECN1 can regulate autophagic and programmed cell death (83). Under normal conditions, Bcl-2 inhibits BECN1, whereas under stress conditions, they dissociate. This allows BECN1 to interact with VPS34 and modulate autophagy (84).

BECN1 dysregulation has been associated with the development of several cancers, including 50%–70% of prostate, breast, and ovarian cancers (85). In response to stressful stimuli, BECN1 can interact with Bif-1 (bax1 interaction factor or endophilin B1) through a mechanism involving UVRAG, thus leading to PI3KCIII and VPS34 activation. This regulatory mechanism has been evidenced in various tumor models (86).

Under cellular stress conditions, autophagy induction mitigates oxidative stress by eliminating damaged mitochondria, a crucial source in ROS production (80). When defective autophagy occurs, debris cannot be removed, leading to increased ROS levels and DNA damage with consequent loss of genomic integrity (87, 88). Therefore, autophagy prevents tumor generation by regulating ROS levels (89).

*Atg* genes expression has been directly linked to this process. Studies in mouse liver with silenced *atg7* and *atg5* showed autophagy malfunction as contributing to benign hepatic adenoma development (90). These tumors did not progress over time, suggesting that the loss of autophagy may be sufficient for tumorigenesis onset, but not for progression towards advanced stages of the disease (91). On the other hand, mice with deficient in *atg4C* showed alterations in autophagy and greater predisposition to fibrosarcomas induced by carcinogens (92).

Numerous tumor suppressor proteins promote autophagy (93–95). Tumor suppressor p53 is a usually deregulated protein in many human neoplasms which promotes autophagy when activated by nutrient deprivation or genotoxic stress (91). p53 functional loss is therefore expected to lead to autophagy inhibition (96). However, p53 can act as either an activator or an inhibitor of autophagy depending on its subcellular localization and its action mode (97). Mice with pancreatic oncogenic alleles for *k-ras* develop precancerous lesions and PDCA (pancreatic ductal adenocarcinoma) over time. Here, p53 expression blocks autophagy, thus inhibiting initial carcinogenesis (98). On the other hand, p53 can contribute to autophagy activation through DRAM1 (DNA damage-regulated autophagy modulator protein 1) (99), *atg7*, and *ulk1* (100) modulation. Another p53 target gene is *isg20l1*, which promotes autophagy induction and cell death when activated (101).

Numerous studies have revealed an increase in autophagy levels as carcinogenesis progresses (89). In metastasis early stages, cells acquire migratory properties and detach from the tumor to enter the bloodstream and flow through the body to colonize new tissue. At this point, autophagy exerts an anti-tumor role by modulating inflammation and cell shedding, but it also promotes motility and invasion (102). These results suggest that the autophagic machinery is a regulatory mechanism that can inhibit tumor generation in the early stages of the disease and in metastasis (**Figure 2**).

### Autophagy as a Pro-Survival and Resistance Mechanism

High metabolism requires a stressful condition to which tumor cells must adapt to proliferate actively in combination with a hypoxic cellular environment (86). Under these conditions, cells can activate autophagy to address various cellular needs and promote oncogenesis (77). Autophagy is activated in the hypoxic regions of tumors to counteract cellular oxygen demand (103). When tumor cells blood supply is insufficient, the autophagic machinery can be activated through an HIF-1-dependent mechanism (hypoxia-inducible factor-1) (15), VEGF (vascular endothelial growth factor) (104), PDGF (platelet-derived growth factor) (105) and oxide synthase (106). Hence, autophagy plays an essential role in promoting tumor cell survival under metabolic stress (107). Furthermore, cell division high rate translates into increased energy and biosynthetic needs, which can be satisfied by rising autophagy levels to obtain ATP and metabolic intermediates (108).

Transcription factor p53 acts as a cellular stress sensor in response to DNA damage and oncogenic stress (94) and often mutates in different types of human cancers (109). Moreover, point mutations in p53 prevent it from inhibiting autophagy in some breast cancer models (96). Consequently, this catabolic signaling pathway is activated to help repair damaged DNA and benefit tumor cells (110).

p62 cell adapter is another crucial molecule in nutrient detection. It can also act as a mitotic transit modulator, an oxidative detoxifying protein inducer, and genomic stability regulator (111). It also contributes to the autophagic mechanism by recruiting proteins and organelles into the autophagosomal compartments for subsequent degradation (112). In liver carcinoma cells, mTORC1 inhibition and ER stress promote p62 accumulation and autophagy induction (113). In colorectal cancer cells, p62 promotes invasion and metastasis by inhibiting apoptosis through a mechanism involving the vitamin D receptor and Nrf2 (114). p62 deletion produces significant autophagy inhibition and affects tumor growth in *in vivo* and xenograft models (115). Upregulated p62 is commonly found in various tumor models (111).

Approximately 33% of neoplasms developed in mammals present mutations of the *ras* gene, which indicates the importance of this gene for medical science (116). Recent studies have revealed that autophagy promotes tumor development, invasion, and metastasis in epithelial cells transformed by this oncogene (117). Furthermore, autophagy inhibition in mice with lung tumors induced by *k-ras* expression was fatal, as specimens died of pneumonia (118). In pancreatic adenocarcinoma models with *k-ras* mutations, tumorigenicity was associated with increases in IL-1 (interleukin-1), NF-kB, and p62 levels (119).

Many patients manifest metastatic bodies many years after the primary tumor has appeared (120). Autophagy can suppress cell division and motility, thus conserving dormant tumor cells energy. At some point, latent cells can reactivate proliferation and colonize new tissue in response to changes in the tumor microenvironment (121). Anoikis is an apoptotic cell death model triggered by insufficient interaction between the cell and the extracellular matrix, a critical factor for transformed cell invasion and metastasis (122). When cells detach from their matrix to enter the bloodstream, autophagy protects them from anoikis and promotes metastasis (123). On the other hand, autophagy contributes to carcinogenesis by inhibiting apoptosis caused by mitochondrial dysfunction and excessive ROS production (124) (**Figure 2**).

## Autophagy Within the Tumoral Microenvironment

**Figure 3:**
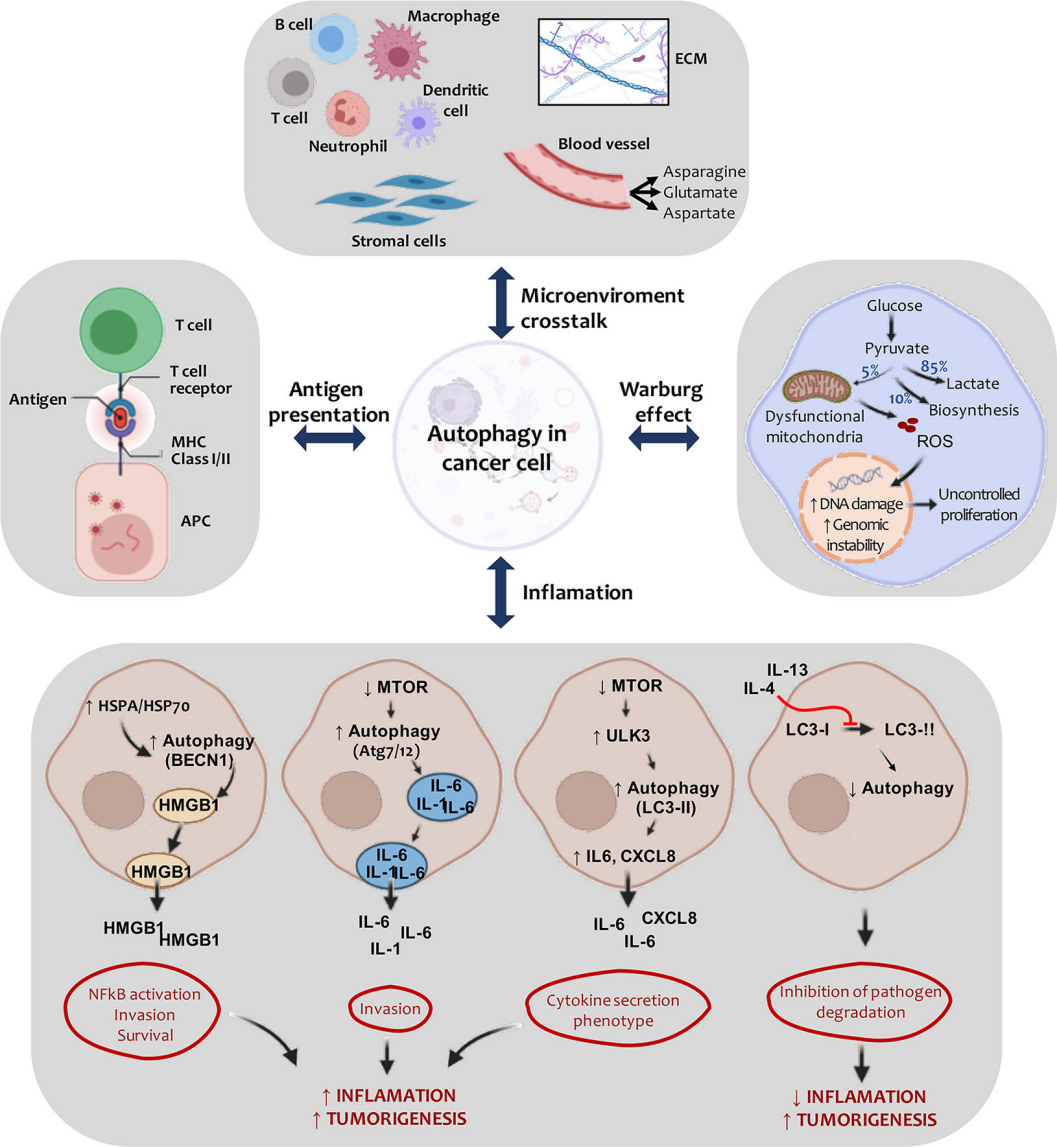
Active crosstalk between autophagy and tumor microenvironment. Carcinogenesis is regulated by autophagy in transformed cells and cells belonging to the tumor microenvironment. Signaling is triggered to the ECM (extracellular matrix) and to stromal cells (such as fibroblasts and pericytes), generating a favorable context for tumor development. As tumor development progresses, cell autophagy activation in the tumor microenvironment serves, in part, to compensate for the inadequate nutritional supply associated with rapidly growing tumors. Here, proper quality control of the mitochondria is necessary to aid glycolysis in tumor cells. In this way, the energy balance (Warburg effect) typically found in malignant cells is maintained. Also, the autophagic process is involved in multiple aspects of lymphocyte development, innate immune signaling and antigen presentation by APCs (antigen presenting cells), processes that are relevant to the disease pathogenesis. HSPA/HSP70 overexpression can induce HMGB1 release in a BECN1-dependent process. This event culminates in the activation of NFκB and promotes tumor proliferation and invasion. Similarly, autophagy induction can contribute to IL-6, IL-1, and IL-8 secretion and promote inflammation. The expression of specific cytokines may inhibit this process, generating a decrease in the inflammatory process.

The tumor microenvironment is the tumor stroma and occupies most of the neoplasm (125). We can therefore hypothesize that as carcinogenesis progresses, the interaction between tumor cells and surrounding stromal cells increases (126). Compared to normal tissues, the tumor microenvironment is characterized by low oxygen levels, high lactate levels, extracellular acidosis, and decreased nutrients (127). It presents great cellular heterogeneity, composed of mesenchymal stem cells, fibroblasts, endothelial cells, immune cells, cytokines, and growth factors (128). Cancer-associated fibroblasts (CAF) are the most studied cells in the tumor microenvironment as they play an active role in tumor promotion (129). These components cooperate to contribute to tumor development (121) (**Figure 3**, top panel).

Autophagy is activated in the tumor microenvironment and in adjacent transformed cells to ensure tissue remodeling, angiogenesis, and interaction with surrounding immune cells (126). Autophagy has been detected in most cells of the tumor microenvironment, but with the ability of performing different actions. For example, when faced with a specific stimulus, the autophagic machinery in fibroblasts promotes tumorigenesis. Simultaneously, in some immune cells, such as cytotoxic T cells, it facilitates immune-response execution against neoplastic cells (130). Under extreme physiological conditions, the stroma activates autophagy to supply energy for adjacent tumor cells (131). In addition, tumor cells can modulate autophagic vesicles’ induction in specific stromal cells (108).

For many years, cancer-related studies focused exclusively on transformed cells, ignoring the tumor environment. At present, the tumor microenvironment is considered to play a fundamental role in tumor development, and its study is essential to form a cohesive idea of ​​what happens within the tumor (132). The extracellular matrix is a fundamental component of the tumor microenvironment. In addition to providing a physical scaffold, it contributes to the secretion of key factors for the tumor’s proper development. Macrophages and fibroblasts associated with cancer are the main cellular models associated with the tumor microenvironment (133).

The bone marrow is the leading site of hematopoiesis and bone formation in most vertebrates as well as the location of inactive and undifferentiated hematopoietic stem cells (HSC) (134). In response to specific stimuli, HSC can differentiate into various blood cells (135). As they have a short life cycle, HSC functional differentiation becomes necessary. During differentiation, cells are exposed to low oxygen levels, a sufficient condition for autophagy induction (136). Recent studies have revealed that autophagy is essential both for self-renewal and for HSC differentiation (137, 138).

After extravasation, monocytes are stimulated by CSF-1 (colony-stimulating factor-1) to induce differentiation. However, this stimulating factor is also associated with autophagy induction through ULK1 activation (139) and PI3K/Akt inhibition (140). CSF-2, another cytokine related to macrophage differentiation, prevents BECN1 and Bcl-2 interaction through a mechanism that includes JNK and triggers autophagy (141). At the same time, G-CSF (granulocyte colony-stimulating factor) contributes to cell survival through apoptosis inhibition and autophagy induction (142).

In recent years, studies related to the role of autophagy and CAFs in the tumor microenvironment have increased (143) and autophagic machinery induction has been associated with the NF-kB pathway and Caveolin-1 (Cav-1) expression in the tumor microenvironment (144, 145). Cav-1 is an essential component of the plasma membrane caveolar, contributing to modulating various signaling pathways. Other scientific sources have revealed a direct relationship between the autophagy induction and BNIP3 (E1B-binding protein 19K/Bcl-2 Nip3) expression in fibroblasts. This induction is accompanied by the loss of Cav-1 expression and the increase in BECN1 and ATG16L (146). Pancreatic stellate cells are a specialized type of fibroblast that can be found in tumor stroma, including PDAC (48, 147). Extensive studies have shown that stellate cells can secrete extracellular matrix molecules and cytokines that contribute to tumor aggressiveness (148). The baseline level of autophagy in PDAC patients is high, and active interaction between the tumor and stromal cell autophagy has been observed (149).

Tumor vasculature is involved in immune cell trafficking and activity. However, it also increases nutrient and oxygen circulation to meet solid tumors’ high energy demands (150). A constant imbalance between pro-, and anti-angiogenic signaling in the tumor microenvironment exists, which contributes to new vessel formation through a VEGF-dependent mechanism (151, 152). Endothelial cells that reside in tumors are exposed to high VEGF levels, nutrient deprivation, and aberrant blood circulation, thus leading to increased autophagy levels (150). In fact, in tumor endothelial cells, autophagy levels are higher than in healthy endothelium (153). Said induction, as mentioned in the previous paragraph, can have anti-angiogenic (154) or pro-angiogenic (155) functions and, therefore, contribute to a different cell fate (156).

### Autophagy as a Regulator of Tumor Immunity and Inflammation

Autophagy can induce immune system cells to exert specific responses (157). Recently, autophagy has been shown to influence not only the antigenic profile of antigen donor cells and their ability to release immunogenic signals (158, 159), but also the survival, differentiation, and function of antigen-presenting cells (APC) (160–162).

Innate immunity is the body’s first line of defense against attack by pathogens; it is an active process and it favors the complement system as well as inflammation (163). At the cellular level, the presence of intracellular pathogens is detected by PRRs (pattern recognition receptors) located in the plasma membrane (TLR (Toll-like receptors), 1, 2, 4, 5, and 6), in endosomal membranes (TLR3, TLR7, TLR8, TLR9) or in the cytosol (NOD (Nod-type receptors), RIG-I (gene I-like receptors), RLR (retinoic acid-inducible receptors), and CLR (C-Type lectin-like receptors) (164). PRRs recognize surface antigens of microbes called PAMPs (pathogen-associated molecular patterns), such as lipopolysaccharides of the bacterial cell wall (LPS), flagellin, bacterial, and viral nucleic acids, and finally, some components of the fungal cell walls (165, 166).

After PPRs recognize pathogens, cells can induce autophagy to eliminate them through lysosomal degradation, although this is highly dependent on cellular context and cell type (167). Evidence has shown several TLRs, including TLR1, TLR3, TLR4, TLR5, TLR6, and TLR7, inducing autophagy in humans and mice macrophages (168). The connection between TLR signaling and autophagy is believed to be mediated by adapter proteins TRIF (adapter-inducing interferon-β containing the TIR domain) and Myd88 (Myeloid differentiation primary response 88), which inhibit the interaction between BECN1 and Bcl-2, thus contributing to autophagy induction (169). Furthermore, the link between Myd88 and mTOR has been reported to allow the activation of transcription factors (IRF-5 (interferon regulatory factor 5), IRF-7) that encode for pro-inflammatory cytokine genes and IFN-I (interferon type-I) (170).

Adaptive immunity, on the other hand, produces/makes a more robust and specific response (171), which involves capturing foreign material by APCs (macrophages, B cells, and dendritic cells) to stimulate T lymphocytes and give specific cellular responses (**Figure 3**, left panel) (172). APCs present antigens to major histocompatibility complex (MHC) molecules through the complex interaction of various cellular factors (173). Autophagy inhibition has been found to reduce MHC-I (MHC-class I) molecules in murine B16 melanoma cells and subsequent cytolysis of tumor cells by CD8+ T cells through cross-presentation (174). CD8+ T cells can respond to exogenous antigens and material undergoing phagocytosis (175). CD4 + T cells recognize antigens from MHC-II molecules (MHC-class II) that are processed in endolysosomal compartments (176). Autophagy may be an essential source of MHC-II antigens derived from intracellular sources through lysosome material supply (177). Like T cells, B cells are regulated by the autophagic machinery. For example, deletion of *atg7* or *atg5* in the hematopoietic system results in a reduced number of peripheral B cells (178, 179). Dendritic cells are responsible for presenting pathogenic antigens to CD4+ and CD8+ lymphocytes, a process that is favored by autophagy induction (180).

Autophagy can regulate immunity and inflammation in tumor transformation in order to regulate carcinogenesis (181). Cytokine signaling is involved in tumor-associated inflammation and has been linked to promoting tumor-initiating cell self-renewal, tumor growth, angiogenesis, and metastasis (182). Cytokine secretion is variable depending on cancer type, but generally involves IL-1, IL-6, CXCL8/IL-8, IL-10, and interferon-gamma (183). In estrogen-receptor-negative breast tumors, IL-1 expression has been associated with autophagy induction with p62 and LC3 accumulation (184). In liver tissue carcinomas, IL-37 expression regulates autophagy by inhibiting the PI3K/Akt/mTOR axis (185). Some cytokines stimulate autophagy (Th1, TNF-α (tumoral necrosis factor α), IL-2), while others inhibit it (Th2, IL-4, IL-13, IL-10) (121) (**Figure 3**, bottom panel).

ROS accumulation in tumor development can cause mutations, protein and mitochondrial damage, and increased secretion of inflammatory and antimicrobial agents (186). An increase of intracellular ROS levels is commonly related to inflammatory signaling activation involving NF-kB and regulating the inflammatory response, angiogenesis, and the function of tumor-initiating cells, according to cellular context (187). Furthermore, interaction between NF-kB and the autophagic machinery in order to alter apoptosis and benefit tumor cell survival is common (181). Studies carried out in a murine model of lung adenocarcinoma have revealed that p62 deletion inhibits tumor development through a mechanism that prevents RELA/65 nuclear localization and NF-kB activation (188).

### Autophagy in Tumor Cell Metabolism Remodeling

In the 1920s, scientist Otto Heinrich Warburg discovered that tumor cells produce more energy than usual and absorb more glucose than healthy cells through glycolysis regulation, a process known as the “*Warburg effect*” (189). Numerous scientific reports support the idea that autophagy can generate ATP through a mechanism that involves glycolysis (**Figure 3**, right panel). Through studies linking these cellular processes, a new paradigm known as the “*reverse Warburg effect*” has emerged. Stromal cells have been postulated as the key generators of fuel for transformed cells (190). Nowadays, both tumor cells and adjacent stromal cells are believed to contribute to meeting tumor energy needs (191). The importance of autophagy in glycolysis has also been observed in chronic myeloid leukemia and breast cancer cells (192, 193). In mice with K-Ras-driven lung tumors, loss of *atg7* leads to defective autophagy, which alters tumor fate, forming benign tumors called oncocytomas (118). These tumor masses with low autophagy levels show defective mitochondria and neutral lipid accumulation (particularly cholesterol esters) due to fatty acid oxidation defects (194).

Mitochondria are central regulators of cell metabolism, which is why they must function correctly. In general, autophagy plays a vital role in the cleaning and quality control of these organelles (195). Both glucose-dependent metabolic pathways and mitochondrial metabolism are essential in tumorigenesis modulation (196). Under hypoxic conditions, pro-apoptotic receptors (BNIP3 and NIX) are activated to induce mitophagy and promote cell survival through HIF-1 regulation (197, 198). In breast cancer cell lines, IGF-I (insulin-like growth factor 1) expression induces BNIP3 expression through a HIF-1-dependent mechanism (199).

Likewise, in glioblastoma cells, PINK1 deletion (the kinase 1 induced by PTEN is a mitochondrial protein of the serine/threonine kinase type) promotes the Warburg effect through ROS and HIF-1 level stabilization. It also reduces PKM2 (pyruvate kinase isoenzyme M2) activity, both regulators of aerobic glycolysis (200). Glycolysis can also be modulated by the interaction between p53 signaling pathways and mitophagy in head and neck squamous carcinoma cell lines (201). Although the molecular mechanism that links these two metabolic regulators has not yet been fully comprehended, Parkin has been found to regulate p53, a ubiquitin ligase that modulates mitochondrial energy metabolism, antioxidant defense, and radiation-induced tumorigenesis (201, 202).

Acetyl-coenzyme A (AcCoA) is a critical metabolic intermediate in autophagy regulation: when its intracellular levels decrease, the autophagic machinery is activated (203). Recent studies in glioblastoma cells have shown that AcCoA increase as a glycolysis product can regulate genes involved in cell migration and adhesion (204). Pancreatic cancers are highly desmoplastic, leading to highly inhospitable environments for cells with high ROS levels, hypoxia, and insufficient nutrient levels (147). Pancreatic stellate cells are a specialized type of fibroblast, commonly found in this type of neoplasm, which contributes to mitochondrial metabolism (205). In the face of stressful conditions, these cells can secrete alanine through an autophagy-dependent mechanism and then be absorbed and used by tumor cells (206). This amino acid fuels the Krebs cycle in PDAC and allows glucose to be used for other anabolic processes such as serine/glycine biosynthesis. Autophagy inhibition in pancreatic stellate cells has recently been shown to decrease tumor growth in transplantation models (149).

HIF-1 and the c-Myc oncogenes coordinated expression regulate cellular glucose transporters, glycolytic enzymes, and mitophagy through choline metabolism (207–209). In B lymphoma cells, c-Myc activates the choline phosphate cytidyltransferase A (PCYT1A) enzyme, inducing mitophagy and preventing cells from dying of necroptosis (210). Arginine is another amino acid that can be dysregulated in cancer cells. Autophagic regulator AMBRA1 (regulator 1 of BECN1 and autophagy) can influence tumor metabolism by regulating c-Myc degradation. When mTOR is inhibited, AMBRA1 is activated. This protein favors the interaction between c-Myc and its phosphatase PP2A (protein phosphatase 2A) to result in the dephosphorylation and degradation of c-Myc, thus reducing cell division rate (211). Cell transformation mediated by c-Myc or RAS-v12 overexpression increases AMPK and FoxO3 expression, which results in increased levels of positive autophagy for BNIP3 and LC3 (212). Finally, the close link between the signaling mechanisms triggered in tumor cells and the adjacent stroma should be taken into consideration; all these events together contribute to tumor metabolism (206, 213).

## Autophagy During Viral Infection

Autophagy is an essential cellular response element for various types of infections. In general, intracellular pathogens are sequestered and selectively degraded by autophagosomal vesicles (214). However, many pathogens use the host cell’s autophagic machinery to survive and spread (68). Viruses are a good example: once inside the cell, they modulate autophagy to regulate almost all viral life cycle, including insertion and entry of the virus into the host cell, exposure of viral components, and viral protein production (215). Some viruses use autophagosomal membranes as anchors in the replication process, while others inhibit autophagy from avoiding being degraded by lysosomal enzymes in autolysosomes (216). A more detailed examination of the molecular mechanisms modulated during viral infection in relation to the autophagy degradation pathway will be made in the following sections.

### Autophagy in Antiviral Immunity Regulation

**Figure 4:**
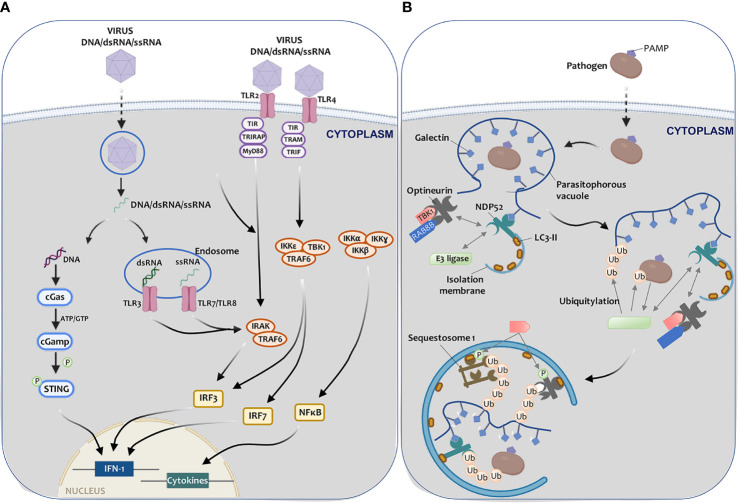
Autophagy-mediated elimination of pathogens. **(A)** Once a virus enters a cell, (I) viral DNA may be exposed in the cytoplasm, leading to cGAS-STING pathway activation and IFN-I expression, thus triggering the antiviral defense; or (II) viral RNA (endosome) can be recognized by TLR3 (dsRNA) and by TLR7 or TLR8 (ssRNA), triggering IRAK and TRAF6, which leads to IFN-I synthesis. Virus recognition by TLR2 or TLR4 may also trigger the cascade that leads to IFN-I (IRF3, IRF7, or NF-kB) and pro-inflammatory cytokine activation transcription factors. **(B)** Once pathogens enter the cell, a parasitophorous vacuole with glycosylated molecules starts enclose them. Galectins present in this vacuole bind to NDP52, which interacts directly with E3 ligase LRSAM1 and indirectly with TBK1. TBK1 interacts with optineurin. NDP52 binds to LC3-II, resulting in xenophagy activation. LRSAM1 polymerize ubiquitin at different targets that are yet to be identified. This hypothetical model includes the pathogen and the parasitophorous vesicle as targets. NDP52 recognizes the ubiquitin tags, optineurin, and sequestosome-I. TBK1 phosphorylates optineurin and sequestosome (I) After these steps, the autophagic isolation membrane elongates to capture the pathogen to degrade it.

Among the various PRRs involved in detecting pathogenic components, TLR receptors located in the plasma membrane and the cytosol stand out (217) (**Figure 4A**). TLRs activation in endosomes requires PAMPs endocytosis, such as viral RNA, damage-associated molecular patterns (DAMPs), apoptotic cells, or autophagy induction (218). Upon activation, TLRs recruit the Myd88 primary response protein or adapter molecule 1 as an NF-kB activator, contributing to the synthesis of inflammatory cytokines, which in turn trigger IFN production (219) (**Figure 4A**). NF-kB, IRF-3, and IRF-7 activation can determine the inflammasome assembly and ultimately result in caspase-1 and IL-1β, and IL-18 activation (220). On the other hand, astrocytes infected with three different Zika virus (ZIKV) strains show an increase in inflammatory molecule release (IP-10, IFN-β, NF-kB) and autophagy activation by a mechanism involving TLR3 (221).

In mice dendritic cells infected with the human herpes simplex virus-1 (HSV-1), *atg5* deletion causes deficiency in the processing and presentation capacity of viral antigens into MHC-II. In this process, TLRs located in endosomal membranes and associated with LC3 have been shown to improve viral antigen processing (222). Moreover, *atg5* has been proven necessary for IFN-α production and TLR9 activation through CpG (DNA regions that make up 40% of gene promoters in mammals, with high concentrations of phosphate-linked cytosine and guanine) in HSV-2 infected cells (223).

The second most important PRRs family are the NOD receptors (224), which constitute the cytosolic counterpart of TLRs and include 23 members in humans (225). NOD1 and NOD2 are two receptors that recognize peptidoglycan, a component of the bacterial cell wall; the stimulation of these two receptors forms a transducer complex called the NOD signalosome (226). This signalosome leads to NF-kB activation, which stimulates chemokines and cytokines production, and which in turn initiates the pro-inflammatory response involved in eliminating pathogens (227). Recent studies have revealed an increase in NOD1 and ATG5-dependent autophagy in hepatic ischemia/reperfusion injuries (228). On the other hand, epithelial cell infection with adherent/invasive *Escherichia Coli* have increased ATG16L1 and LC3 through interaction with NOD2 cytosolic receptor (229).

The CRL receptors family binds to carbohydrates present in pathogens (lectins are proteins that recognize sugars) through a Ca^2+^ dependent mechanism (230). Type C lectin receptor, Mincle (macrophage-inducible Ca^2+^ dependent lectin receptor), and TLR4, induce autophagy by activating Myd88 in macrophages (231). RIG-I receptors are characterized by having a C-terminal regulatory domain and a DExD/H helicase domain that contribute to recognizing and unwinding the viral RNA duplex (232). Melanoma differentiation-associated protein 5 (MDA5) is another essential protein in this family. Both proteins detect viral RNA in the cytosol by interacting with mitochondrial signaling through its caspase recruitment domain and regulating autophagy (233).

The last group of receptors includes cytosolic DNA and RNA sensors. An excellent example of these sensors is the cyclic GMP-AMP synthase (cGAS), which plays a fundamental role in recognizing DNA viruses and IFN pathway induction (234). Excessive IFN stimulation can damage the body, so complex regulatory mechanisms have been developed, one of them being autophagy (235). After infection with HSV-1, BECN1 interacts with cGAS, thus altering its nucleotidyltransferase function and triggering the autophagic machinery. When free in the cytosol, cGAS recognizes virus DNA and activates IRF-3 and STING (interferon gene stimulator) to increase IFN production (236) (**Figure 4A**).

Autophagy can also modulate the adaptive immune response to infection through intracellular pathogens. MHC-I molecules are responsible for presenting pathogenic antigens to CD8+ T lymphocytes in order to trigger different cellular responses, such as endocytosis, vesicle trafficking, and autophagy (237). In the conventional mechanism, the proteasome breaks down these protein antigens into peptide fragments and transports them to the endoplasmic reticulum, where their processing concludes (238) (**Figure 4B**). Some cells, such as dendritic cells, can present pathogenic antigens to lymphocytes through “*cross-presentation*” (239). This mechanism can occur through three different signaling pathways (240). In the first case, pathogens are recognized and transported to the proteasome, where small peptide fragments are released and transported to the ER by TAP1 (transporter associated with antigen processing 1) and TAP2 to be presented to HMC-I molecules (240) (**Figure 4B**). The second pathway is independent of the proteasome, and its lysosomal proteolysis helps facilitate antigen processing (241). Finally, in the last signaling pathway, degradation is proteasomal but independent of TAP (242).

The various death mechanisms involved in dendritic cell cross-presentation have been assessed in comparative studies, which show that *atg5* inhibition also inhibited said presentation. This finding has made it possible to associate autophagy with antigens effective presentation to CD8 + T cells (243). Subsequent studies have revealed that autophagy not only influences antigen processing on HMC-I molecules but that is also a prime antigen source for HMC-II molecules, such as CD4 + T cells (244).

### Viruses Can Activate or Inhibit Autophagy in Favor of Their Replication

Viruses are particles that cannot survive on their own. This is why they have evolved alongside their respective hosts, a process that has given them the ability to use host cell signaling pathways to their advantage (245). To this end, viral particles promote the expression of various viral proteins that mimic host protein structure and function (215). These proteins modulate many cell signaling pathways in favor of viral replication, and autophagy is not exempt from this regulation (246, 247). Viruses with RNA in their genetic material usually contribute to autophagic membrane accumulation, regardless of whether their replication is nuclear or cytosolic (248).

Many viruses that regulate autophagy to facilitate viral survival and replication have been discovered, including poliovirus (249, 250), Coxsackievirus (CVB3) (251, 252), CVB4 (253), Enterovirus 71 (EV71) (254), human rhinovirus (HRV) (255), foot-and-mouth disease virus (FADV) (256), encephalomyocarditis virus (EMCV) (257), dengue virus (DENV) (258, 259), ZIKV (260, 261), mouse hepatitis virus (MHV) (262), Newcastle disease virus (NDV) (263), severe acute respiratory syndrome coronavirus (SARS-CoV) (264), Chikungunya virus (CHIKV) (265), and Japanese encephalitis virus (JEV) (266), among others.

In cells infected with human poliovirus, viral proteins 2BC and 3A promote the formation of autophagic vesicles where viral replication takes place. The virus induces tubular structures in early stages of infection, while forming double-membrane vesicles in advanced settings (267, 268). Recent studies have provided a novel and deeper understanding, by revealing that these viruses can regulate autophagy through a ULK1-independent mechanism (269). During infection with EV71, the ERK inhibition pathway and autophagy impairs viral replication (270).

Measles virus (MeV) belongs to the *Paramyxoviridae* family and manifests itself mainly in children as high fever, acute respiratory infections and typical papular rashes (271). MeV binds to the host cell through CD46 (CD46 complement regulatory protein), a receptor on the plasma membrane that initiates the autophagic cascade when activated (272). Once active, this receptor binds to the VPS34-BECN1 complex *via* the GOPC scaffold protein (containing Golgi-associated PDZ and spiral-spiral motif) (273). Virulent MeV samples recognized by CD150 membrane receptors have not shown to have the ability to regulate autophagy in early stages of infection. However, these strains modulate autophagy late in the mechanism to prevent cell death and benefit viral replication (274).

The human immunodeficiency virus (HIV) is a member of the *Retroviridae* family, affecting more than 30 million people worldwide (275). This human disease progresses towards immune system failure, resulting in infection development or tumor transformation (276), generating alterations in the host’s signaling pathways, and therefore damage accumulation. Critical regulation aspects of the cell cycle are altered, which determine cell transformation and tumor progression, mainly of B cells (277). HIV tat protein, HIV-induced immunosuppression, and a hyperinflammatory state facilitate the oncogenic activity of Kaposi’s sarcoma-associated oncovirus (278). Average survival after HIV infection is estimated to be nine to eleven years without treatment, depending on HIV subtype (279). This retrovirus is transmitted by body fluids and infects CD4+ lymphocytes and macrophages, ensuring prevalence in the host through complex cellular processes (280). In macrophages, autophagy can contribute to HIV degradation or replication. Nef viral protein blocks autophagy initiation by promoting BECN1 binding to Bcl-2 through a PRKN-ligase dependent mechanism (Parkin RBR E3 Ubiquitin Protein Ligase) (281). In studies on CD8+ lymphocytes infected with HIV from patients with lymphoblastic leukemia, the virus was able to inhibit autophagy by reducing ATG8 and BECN1. The opposite result was seen in HIV-infected CD4+ cells (282). Furthermore, an active modulation of ATG1, ATG4D, and ATG5-ATG12 proteins by the virus has been shown. The findings reported so far indicate that HIV can modulate autophagy at different stages to benefit its replication and escape cell degradation (215, 283).

The group of RNA viruses belonging to the *Coronaviridae* (CoV) family has recently gained relevance. SARS-CoV-2 is the etiological agent of COVID-19, a disease that has had a devastating impact in the past year around the world (284). Before COVID-19, six human CoV pathogens had been identified, two of which are aggressive enough to develop massive infections (285). SARS-CoV infected cells can use the autophagic machinery to degrade viral particles or promote their replication and prevalence. However, more studies are needed to better understand the signaling pathways involved in these viral replication processes (286).

The influenza virus (family *Orthomyxoviridae*) is another group of viruses implicated in developing human respiratory diseases. Three influenza serotypes have been characterized, only one of them responsible for generating epidemics annually and pandemics at irregular intervals (287). Viral protein M2 is a proton channel that facilitates the acidification of viral particles and allows their decomposition in the host cell nucleus (288). This protein also blocks autophagosome degradation and redirects LC3 to the plasma membrane, generating a cellular redistribution of membranes coupled with this protein. Through this process, these viruses can lead to the formation of filamentous buds, which appear to increase virus stability (289).

CHIKV is transmitted to humans by the bite of some mosquito species and can induce the autophagic machinery through ER stress, increased ROS levels, and reactive nitrogen species (290). Some studies have reported that when the virus is actively replicating, it induces autophagy through the AMPK pathway. However, this has not been observed when the virus is latent (291). Another virus transmitted to humans by mosquitoes is DENV, a *Flaviviridae* family member that can cause acute or chronic infections (292). These viruses replicate in ER invaginations, so autophagy does not have a structural role in replication. However, cells activate lipophagy to break down cellular triglycerides as well as increase B oxidation and energy production (293). Notably, infections caused by ZIKV, an RNA flavivirus, has generated epidemic outbreaks throughout the world from 2007 to the present (294). A recent study has shown that autophagy can facilitate viral replication through autophagosomal vesicle production or inhibit it in *in vivo* and *in vitro* models (295)

### Oncovirus and Autophagy

Traditionally, cell transformation has been associated with chronic exposure to various carcinogens, such as ionizing radiation and chemical carcinogens, or to genetic predisposition. However, scientific evidence linking viral infections to tumor development has increased (296). Currently, between 15% and 20% of neoplasms are considered to be related to primary viral infections (297). These oncogenic viruses integrate into the host cell genome and utilize host signaling pathways to regulate cell proliferation and differentiation, genomic stability, apoptosis, and immune system recognition (298, 299). Oncoviruses can be classified as direct and indirect carcinogens, although there is some overlap between both. Indirect regulation is related to chronic inflammation modulation contributing to carcinogenesis (300).

Human viral oncogenesis is a complex process in which only a low percentage of individuals develop cancer years after viral infections (301). During this coexistence between the virus and the host, multiple genetic and epigenetic alterations accumulate, contributing to oncogenic pathway dysregulation. In this context, oncoviruses are considered necessary but not sufficient to determine cell transformation (302). Patients with this type of cancer have reduced viral replication, which is required for the cell to actively divide. The virus remains within the cell as a naked nucleic acid in the form of a plasmid or an episome, or it integrates into the cell genome and remains latent (303). Co-evolution of viruses with hosts has shown that the autophagic machinery can be used alongside various proviral and antiviral functions, depending on virus type, cell, and cellular environment (304, 305).

**Figure 5:**
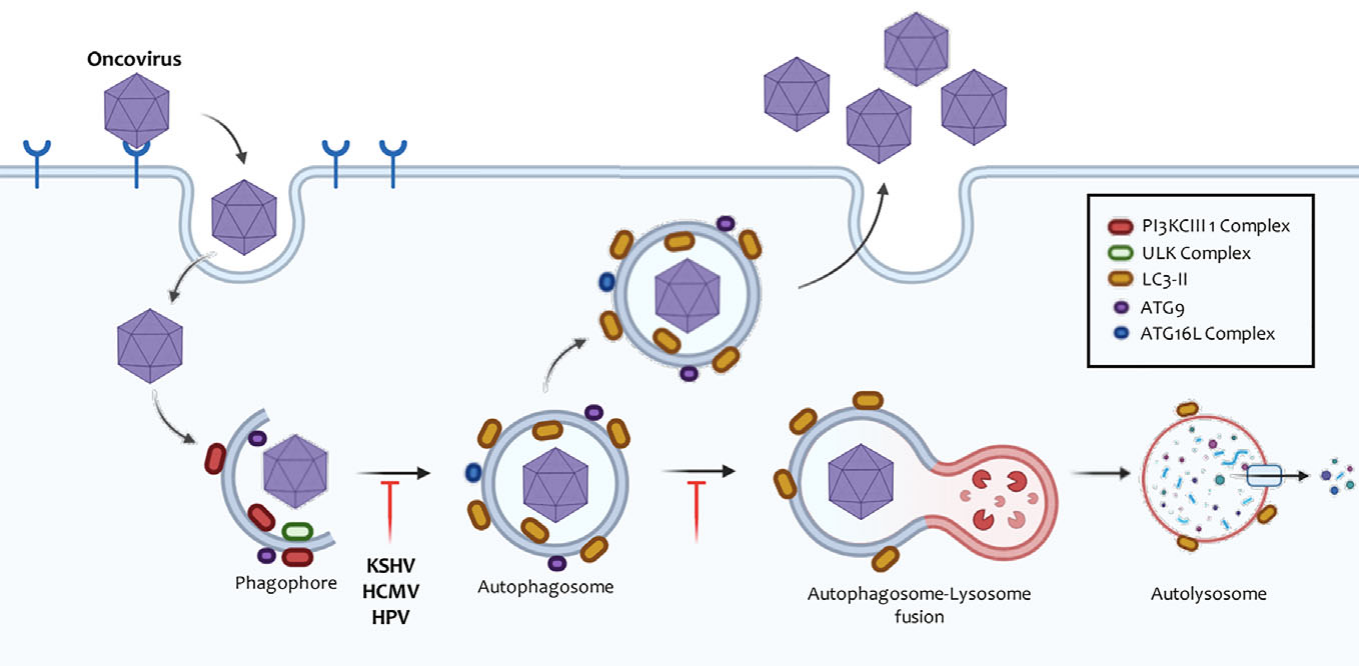
Manipulation of autophagy by oncoviruses. Oncoviruses regulate autophagosome formation or degradation in order to promote their replication cycle. Like many DNA viruses, Herpesviruses prevent their degradation by inhibiting the creation of these vesicles, whereas RNA viruses often induce the accumulation of autophagic membranes independent of their replication in the cytosol or the nucleus. These autophagic membranes can form autolysosomes and finally conclude. In other cases, the autophagic membranes may be used as scaffolds, where the viral replication complexes are positioned or serve as support for the release of the particles. Poliovirus was the first virus where this mechanism has been evidenced.

Up to date, eight different oncogenic viruses have been characterized (**Table 1**) and are described as follows. The human papillomavirus (HPV) and the Merkel cell polyomavirus (MCPyV) are both involved in the development of neoplasms associated with mucosa and skin (306). Hepatitis B virus (HBV) and hepatitis C virus (HVC) are associated with 80% of hepatocellular carcinomas (HCC) (307). The herpes virus associated with the development of Kaposi’s sarcoma (KSHV) and the Epstein-Barr virus (EBV) are mainly viruses associated with endothelial carcinogenesis (308). Cytomegalovirus (HCVM) is another herpes virus that has an onco-modulatory function (309). And human T-cell lymphotropic virus-1 (HTLV-1) is an RNA retrovirus responsible for the development of adult T-cell tumors (ATLL) (310). These viruses can modulate oncogenic responses by regulating autophagy at different points (300, 311) (**Figure 5**).

**Table 1:** Oncogenic viruses and their involvement in autophagy.

Virus	Genetic Material	Capsid	Involvement in autophagy
HHV-8 (KSHV)	dsDNA	Icosahedral capsid embedded in integument, surrounded by a lipid envelope with glycoproteins	Latency: autophagy is decreased - vFLIP inhibits activation of ATG3 and LC3 proteins - LANA binds to viral and host DNA ensuring its stability and inhibiting autophagy Lytic cycle: autophagy is activated - mTORC1 is required for RTA synthesis and viral transport
HHV-4 (EBV)			Latency: PI3K/Akt stimulation inhibits autophagy - LMP-1 activates mTOR to suppress autophagy and facilitate cell growth and proliferation Lytic cycle: autophagy is activated - BALF0 and BALF1 are expressed, both with a region of interaction with LC3 necessary to modulate autophagy induction - RTA promotes autophagy through regulation of ERK1/2
HTLV-1	ssRNA		Early stages: autophagosomes accumulation that contributes to viral replication - Tax protein stimulates BECN1 and NFkB leading to formation of autophagosomes - p47 degradation induces NFkB Late stages: autophagy inhibition - BHZ activates mTOR, blocking autophagosomes binding to lysosomes
HCMV	dsDNA		Primary infection: autophagy is induced - Increased levels of LC3 - TLR2 activation by glycoproteins in the viral envelope One day after primary infection: - TRS1 and IRS1 associate with BECN1 inhibiting autophagy
HCV	ssRNA	Lipid bilayer with 2 glycoproteins: E1 and E2. A non-icosahedral nucleocapsid surrounds the lipid bilayer.	It has been reported an increase in the number of autophagosomes accompanied by decrease of their degradation. Autophagic machinery is necessary for viral translation but becomes expendable once the viral replication process begins. Selective autophagy is induced in some organelles such as mitochondria and lipid deposits in cells. ATG5-12/16L1 protein complex is recruited to membrane, contributing to viral replication.
HBV	dsDNA		SHB protein generates a massive induction of autophagy HBx induce autophagosome formation through BECN1 modulation HBx represses V-ATPase, decreasing acidification of lysosomes, inhibiting autolysosome degradation, contributing to viral development.
HPV	dsDNA		Primary infection can induce autophagic machinery (ATG7, BECN1, and p62 are induced). After primary infection, mTOR is activated, suppressing autophagy, what protects capsid degradation in autophagosomes

## Human Herpesvirus

Herpesviruses are biological structures that have linear double-stranded DNA, ranging from 100 to 200 kb. The viral genome is packaged in an icosahedral capsule embedded in a protein integument and surrounded by a host-derived lipid envelope (312). The viral envelope has glycoproteins that mediate the union with the cytoplasmic membrane of the host for subsequent entry of the virus accompanied by the nucleocapsid and the integument. The capsid travels through microtubules until it reaches the nucleus, coupled with protein complexes that allow nuclear pore opening. It then injects its genetic material into the nucleus (313).

Eight herpesviruses have been identified in humans with a common evolutionary origin, classified according to their genomic sequence and biological characteristics into three subfamilies (alpha, beta, and gamma) (314). Within the gamma-herpesviruses, we can find human herpesviruses 4 (EBV) and 8 (KSHV). These viruses have a high affinity for B lymphocytes, and both establish latent and lytic infections that promote the development of hematological diseases and various types of solid human cancers (315). Surprisingly, the ability of viruses to regulate autophagy can vary according to infection stage (316). HCMV is another herpes virus that primarily infects fibroblasts, but which can also be found in epithelial cells, endothelial cells, hepatocytes, stromal cells, monocytes/macrophages, astrocytes, and neural stem/progenitor cells (309).

### Kaposi’s Sarcoma Virus

KSHV was discovered in 1993 from a tissue sample of a patient with Kaposi’s sarcoma (KS) (317). This virus is generally transmitted by body fluids, although transmission has been observed through the placenta to the fetus in some rare occasions (318, 319). KSHV seroprevalence is estimated to range between 5%–20% worldwide. Yet, only a small number of patients develop secondary diseases associated with the virus, the population mostly at risk being immunosuppressed individuals or individuals with immune system abnormalities, as is the case of AIDS patients (320). KSHV infection is associated with the development of various human pathologies, including Kaposi’s sarcoma, primary effusion lymphoma (PEL), multicentric Castleman’s disease (CMD), and inflammatory cytokine syndromes (KICS) (321). The main reservoir of this virus in its latent form are B lymphocytes, but it can also infect monocytes, fibroblasts, endothelial, epithelial, and dendritic cells through association with receptors on the plasma membrane (322).

After primary infection, the virus remains in the cell as an episome and regulates various host signaling pathways to replicate correctly. The virus produces proteins associated with the viral latency phase which are essential for cell transformation (323). Some of the encoded proteins are complement-fixing proteins (v-CBP), viral interleukin-6 (v-IL-6), viral inflammatory protein type-I (v-MIPI) and type-II (v-MIPII), viral Bcl-2 (v-Bcl-2), viral interferon regulatory factor (v-IRF), viral cyclin (v-Cyclin), latency nuclear antigen (LANA), viral adhesin (v-ADH), receptor-coupled G-protein (v-GCR), thymidylate synthetase, thymidine kinase, ribonucleotide reductase (300, 324)

In various models, autophagy has been found to be a cellular mechanism commonly regulated by viral KSHV proteins (325). After initial expansion at the infection site, the new viral particles spread throughout the body and reach the cells in order to establish a latency phase, especially in the B lymphocytes and endothelial cells of the blood/lymphatic vessels (326). During the latency phase, autophagy and other cellular mechanisms contribute to creating a cellular microenvironment favorable to tumor initiation and progression (321).

**Figure 6:**
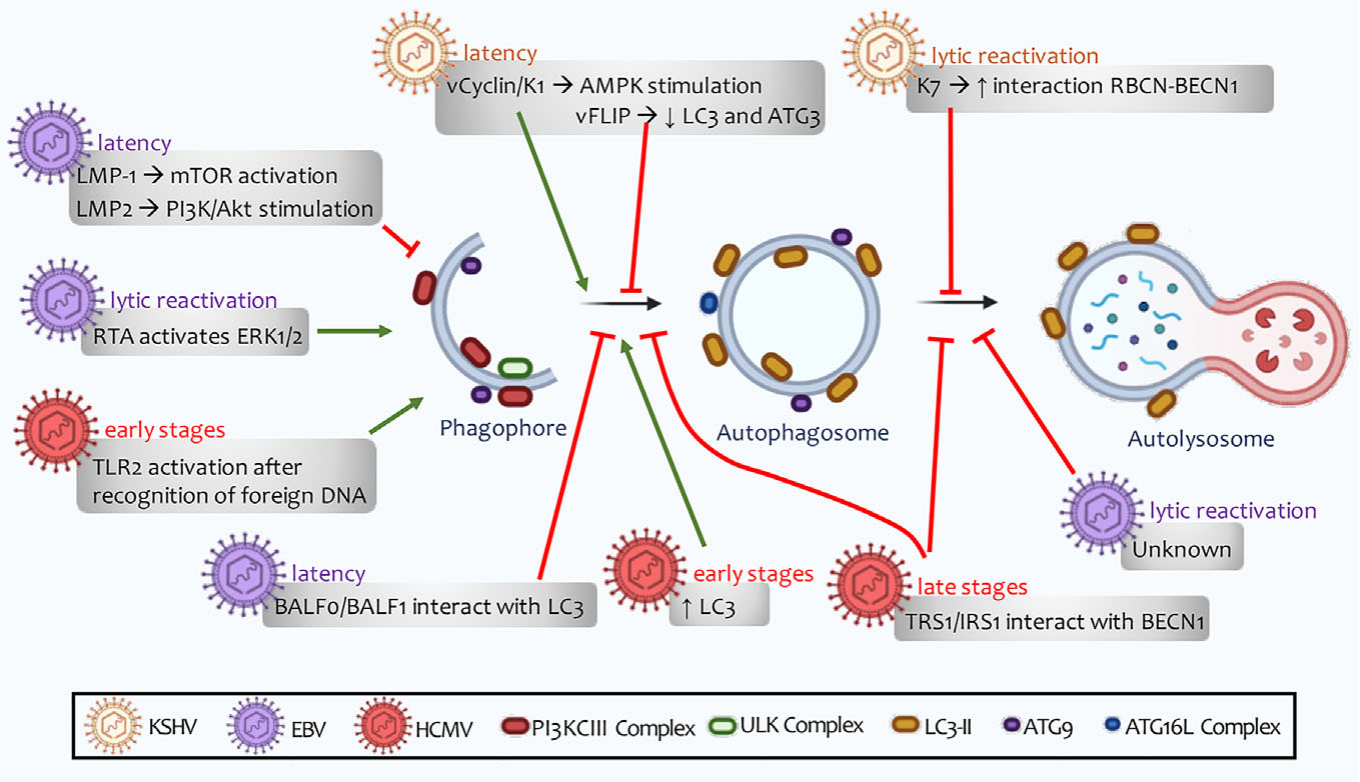
Human Herpesvirus regulation of autophagy. The viral ability to regulate autophagy depends on stage of infection. This regulation can alternate between latent, *de novo*, or lytic reactivation infections. A distinguishing feature of these lymphotropic viruses is their ability to promote autophagy induction, a process followed by inhibition of autophagosome maturation. This defective autophagy does not allow autolysosomes to form, and therefore to perform successful clearance.

The transmembrane glycoprotein K1, encoded by the first KSHV open reading frame, is a signaling protein capable of causing B cell activation (327). V-cyclin and K1 have been found to promote autophagy by stimulating the AMPK pathway (328, 329) (**Figure 6**). On the other hand, vFLIP restricts the autophagic machinery by inhibiting ATG3 and LC3 proteins (330) (**Figure 6**). Once viral latency is established, LANA plays a fundamental role in maintaining this phase through NF-kB activation (331). Granato et al. observed viral particles inside autophagic vesicles in the cytoplasm of PEL cells in active replication, thus postulating that autophagy may also be related to viral transport (332). Finally, another protein linked to viral latency is STAT3 (signal transducer and activator of transcription 3), which remains active in a state of viral latency (330). In dendritic cells, KSHV infection induces STAT3 phosphorylation, promoting cell survival and viral latency. Moreover, the release of IL-10, IL-6, and IL-23, cytokines that contribute to keeping STAT3 active, is also induced (333). This allows the viral genome to remain unchanged and the particles to replicate successfully (334).

Numerous cellular events can activate lytic KSHV reactivation. Cellular microenvironment can shape the viral epigenome to facilitate latency reactivation (335). ER stress can induce *rad21* cleavage, a member of the cohesin complex that generates dramatic changes in the KSHV genome. The loss of DNA loops triggers virus reactivation (336). During this process, cells activate the autophagy machinery with various functionalities (335).

A fundamental component for lytic reactivation is the expression of lytic switch master proteins (RTA), targeting 100 different sites in the KSHV genome and transactivating 34 lytic genes (337). Pringle et al. have reported that mTORC1 is required for lytic replication and RTA synthesis as an activator of cellular transcription. In contrast, this autophagy inhibitor complex has not shown more significant involvement in genomic replication, late gene expression, or in the release of infectious progeny (338). Furthermore, autophagy inhibition has been reported to reduce lytic KSHV reactivation (339).

In contrast to the nuclear LANA function, cytoplasmic isoforms of this viral protein mediate lytic reactivation by antagonizing cellular DNA sensors. These isoforms bind to cGAS, a process that involves STING and NF-kB induction (340). Viral protein K7 expression stimulates RBCN (Rubicon autophagy regulator) interaction with BECN1. These events promote the blocking of autophagosome maturation (333) (**Figure 6**). KSHV monocyte infection counteract ROS increase induced by macrophage colony-stimulating factor (M-CSF), preventing JNK and Bcl-2’s phosphorylation and inhibiting autophagy. Together with the decrease in TNFα and the increase in the immunosuppressive cytokine IL-10, all these events lead to impaired macrophage survival and differentiation (341). Findings so far allow us to infer that these viruses may induce autophagosomes formation, in which they are transported to the cell surface. Viral proteins inhibit lysosome-to autophagosome-binding and, therefore, autolysosome formation is not degraded by lysosomal hydrolases (330) (**Figure 6**).

### Epstein Barr-*Virus*

EBV is a very easily transmitted herpesvirus that is mainly contracted in childhood through body fluids. 90% of the world’s population is believed to have been infected at some point of their lives (342, 343). This virus has contributed to the development of various secondary pathologies, such as infectious mononucleosis and some neoplasms of epithelial and lymphocytic origin (344). After entering host cells, viruses amplify and rapidly enter a state of latency. A distinct fact is the presence of three different latency types that can be independently regulated, autophagy playing an essential role in this regulation (345, 346). Each cell presents multiple copies of viral DNA episomes and produces a series of proteins associated with latency, including six nuclear antigens (EBNA 1, 2, 3A, 3B, 3C, and -LP) and three latent membrane proteins (LMP1, 2A and 2B) (347).

LMP2 viral protein has been reported in most neoplasms associated with this virus. This protein stimulates the PI3K/Akt signaling pathway, a process that triggers apoptosis and autophagy inhibition, thus contributing to tumor cell proliferation (348) (**Figure 6**). This regulation has been seen in gastric carcinomas associated with EBV infection, where the presence of PI3K mutations has been associated with higher tumor occurrence and metastasis (349). Furthermore, LMP2 has been found to contribute to cell proliferation through p27 degradation (350, 351).

Additionally, LMP1 binds to membrane-bound death receptors TRAF and TRADD (tumor necrosis factor receptor type 1-associated death) or activate signaling pathways that include NF-kB, JNK, p38, small GTPases (Cdc42), and the JAK/AP-1/STAT cascades. Moreover, LMP-1 has been reported to activate mTOR to suppress autophagy and facilitate tumor growth and proliferation (352) (**Figure 6**). In contrast, Hurwitz has shown that LMP-1 can inhibit mTOR by secreting CD63-dependent vesicular proteins, contributing to autophagy induction. This induction is not complete since LMP1 inhibits lysosomes’ binding to autophagic vesicles in order to avoid the viral particles’ degradation by lysosomal hydrolases (353). On the other hand, EBNA3C nuclear antigen activates autophagosome formation through transcriptional induction of several autophagy regulators, including ATG3, ATG5, and ATG7 (354).

**Figure 7:**
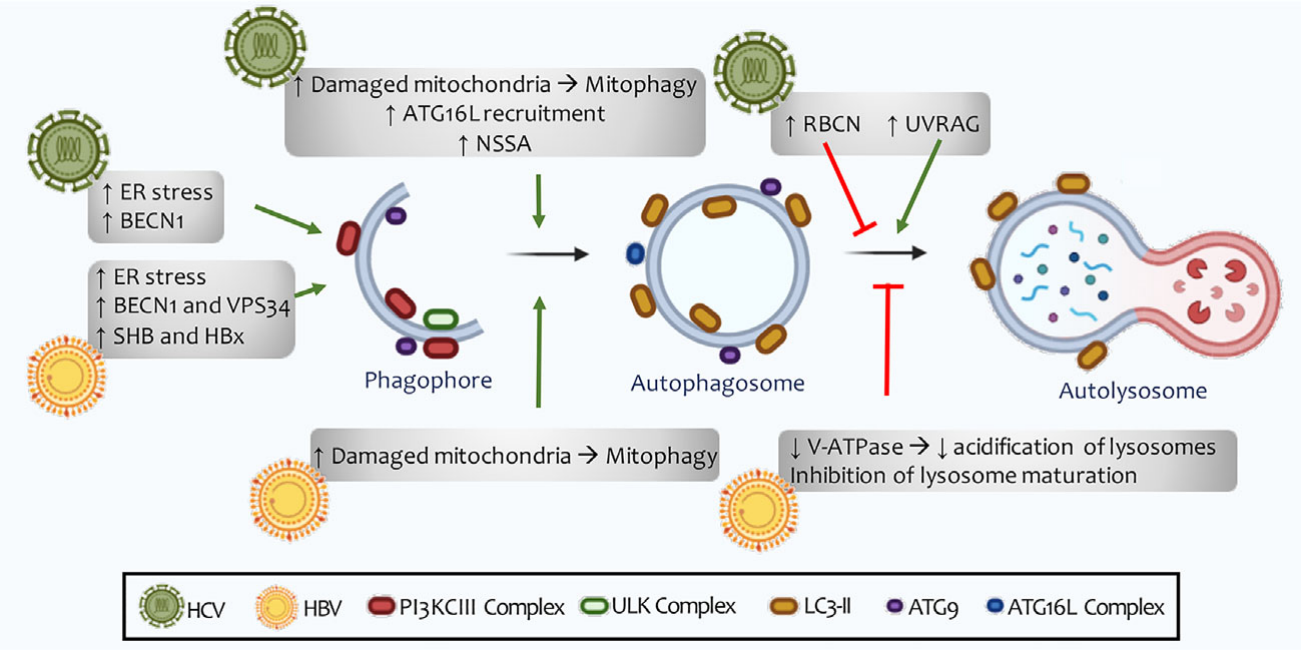
HCV and HBV regulation of autophagy. The autophagic machinery is necessary for incoming viral RNA translation, but it becomes expendable once the viral replication process begins. An increase in the number of autophagosomes does not correlate with an increase in the rate of lysosomal degradation. HCV has been shown to induce the expression of Rubicon, thus inhibiting the maturation of autophagosomes. For its part, HBC reduces the acidification of lysosomes and autolysosomes through the inhibition of V-ATPase (H + type vacuolar enzyme). However, *in vitro* studies show good fusion of autophagosomes with lysosomes, as well as successful autophagic flow, indicating that these viruses are able to induce selective autophagy in some cellular organelles, such as the mitochondria and lipid deposits in cells.

During the EBV lytic cycle, autophagy may present bimodal modulation, showing an early stimulation phase in combination with the inhibition of the late phases of the autophagic mechanism (degradation of cytoplasmic material by lysosomal hydrolases) (355). This final regulation favors the acquisition of envelopes and components of the autophagic machinery by newly synthesized virions (356, 357). The viral proteins associated with this virus that regulate autophagy in the EBV lytic cycle have not yet been fully characterized. RTA function, an early expression protein regulating autophagy through an ERK-dependent mechanism, has been recently highlighted (358) (**Figure 7**).

EBV codes for the expression of two Bcl-2 homologous viral proteins called BHRF1 and BALF0/1 (359, 360). Both viral proteins prevent apoptosis during early B-cell infection but may be dispensable once the latent infection is established (361). BHRF1 anti-apoptotic activity has been studied extensively (362). However, BALF0/1 expression and function remain ambiguous. Two in-frame methionine codons are present near the beginning of the BALF0/1 open reading frame (ORF), suggesting that two proteins with different N-termini may be encoded (363). So far, the BALF1 protein is known to be encoded by the shortest ORF, while the BALFO protein is encoded by the first non-conserved methionine. During the lytic cycle initial phase, BALF0 and BALF1 are expressed, both with a region of interaction with LC3, and modulate autophagy induction (364). So far, we can infer that EBV inhibits autophagy during the latency phase in its initial steps. Simultaneously, during lytic reactivation, the formation of dysfunctional autophagosomes is promoted (**Figure 6** **)**.

### Human Cytomegalovirus

HCMV is a double-stranded DNA herpes virus transmitted through body fluids, not prevalent in any particular age range. This virus has an extensive genome of 236 kilobases, one of the largest viruses that can infect humans (365). Primary infections are generally asymptomatic, although congenital diseases can lead to various severe disabilities or fetal death (309).

HCMV particles have been detected in different cell types, including epithelial cells, connective tissue, hepatocytes, various populations of leukocytes, and vascular endothelial cells (366). HCMV also infects tumor cells and contributes to transformation when affecting healthy tissue by modulating various cellular signaling pathways (309). This virus regulates autophagy in a bimodal manner (367). First, during early stages of infection, it induces autophagic vesicle formation. Later in infection, HMCV blocks autophagy through viral proteins synthesized in the host cell (368). Two proteins involved in autophagy inhibition by association with BECN1 (TRS1 and TRS2) have been identified (**Figure 6**). Mouna et al. has found that co-expression of viral proteins TRS1 and IRS1 is essential for autophagy inhibition in various cell models (369).

Recent attention has been given to viral components that determine HMCV latency and lytic reactivation, with special focus on the *uLb’* gene locus (*ul133-138*) that restrict viral replication by modulating viral latency and immune evasion through the expression of a considerable number of viral proteins (370). An example of this is UL138: this viral protein is presented to HMC-I to regulate the host’s adaptive immunity in fibroblast, and the autophagic machinery holds this event (371). In contrast, it was reported that autophagy inhibition generates a high response of CD8 + lymphocytes due to the internalization of molecules in MHC-I (372).

Early expression of viral proteins associated with HMCV genes 1 and 2 (IE1 and IE2) is necessary for lytic reactivation of host cell virus and immunomodulation (373). IE2 can interact with itself and UL84 as well as with many specific cell transcription factors to regulate gene expression. This protein plays a critical role in viral DNA synthesis and is also considered to counteract host response (374, 375). IE2 overexpression has been recently found to induce autophagy in HMCV-infected cells (376). These results show that in the early stages of HCMV infection, viral proteins contribute to autophagosomal vesicle formation. At the same time, they inhibit vesicles-to-lysosomes binding in later stages, thereby losing their degradative capacity (**Figure 6**).

## Human T-Cell Lymphotropic Virus Type 1

HTLV-1 is a member of the *Retroviridae* family and is represented by a small single-stranded RNA genome approximately nine kilobases long (377). This retrovirus was first isolated in 1979 in samples from patients with cutaneous manifestations of rapidly growing T-cell lymphoma (ATLL) (378). Furthermore, HTLV-1 is associated with the development of poliomyelitis, HTLV-1 associated myelopathy, infectious dermatitis, arthropathy, and facial nerve palsy (379). Evidence suggests that there are between 5 and 20 million HTLV-1 carriers worldwide, but only 3-5% of them develop secondary pathologies (380, 381).

Immediately after entering the host cell, the viral RNA undergoes reverse transcription, and then binds to the cell’s genome as a provirus. This binding generally occurs in areas close to the binding sites of transcription factors such as STAT1, TP53, and HDAC6 (histone deacetylase 6). Dysregulation generates alterations in cell signaling in the expression of specific genes and autophagy is not exempt from this viral regulation.

Regulatory protein HTLV-1 Tax is an oncoprotein that plays an essential transcriptional role in viral replication and participates in T lymphocytes’ transformation. It can also transactivate or transrepression more than 100 cellular genes by linking and modulating stability and activity (300). Recently, HTLV-1 infection has been reported to induce autophagosomes in cells and inhibit their binding to lysosomes through a tax-dependent mechanism. In this way, the number of non-degrading autophagic vesicles, where viruses can replicate, increases considerably (382). Therefore, Tax viral protein, located in the plasma membrane’s lipid microdomains, binds to the IKK complex to stimulate BECN1 and NF-kB activity (383).

Cell adhesion molecule 1 (CADM1) is a member of the immunoglobulin superfamily and is considered to be an excellent cell surface marker of HTLV-1 infected T cells (384). For CADM1 to be correctly expressed, Tax and NF-kB induction and p47 (a negative NF-kB regulator) degradation are necessary. Autophagy is p47 primary degradation mechanism, and it is active in most ATLL cells infected with HTLV-1 (385).

Another essential viral protein for ATLL development is HBZ (bZIP factor) (303). HBZ inhibits both apoptosis and autophagy and may induce the expression of brain-derived neurotrophic factor (BDNF) and its receptor (386). According to the subcellular location of HBZ, it contributes to tumor progression (nuclear) or favourably contributes to inflammation induction (cytoplasmic) (387). When HBZ is exported from the nucleus to the cytoplasm, it activates mTOR through the DNA damage-inducible protein PPP1R15A (a regulatory subunit of protein phosphatase -1 15A) (386). As in other viral infections, HTLV-1 induces autophagosome formation and inhibits binding to lysosomes to prevent degradation. Consequently, the number of autophagosomal vesicles in the cytosol increases, creating a physical obstacle for developing other cellular processes and a favorable environment for viral particle formation (388).

## Hepatitis C Virus

HCV belongs to the *Flaviviridae* family, characterized by viral particles with an RNA helix of 9.6 kb in length and wrapped in a lipid bilayer with two anchored glycoproteins (E1 and E2). In general, it presents a non-icosahedral nucleocapsid, although it is possible to find viruses without nucleocapsid in infected patients’ blood (389). Chronic HCV infection can trigger liver fibrosis and cirrhosis, and it is also associated with hepatocellular carcinoma (HCC) and non-Hodgkin lymphomas development (390). This virus can promote carcinogenesis directly through the modulation of specific signaling pathways and indirectly through chronic inflammation (391).

Once inside the host cell, the virus forms a membranous network used during replication (392). Three membranous rearrangements associated with the virus have been identified: cluster vesicles, contiguous vesicles and double-membrane vesicles. In general, cluster vesicles are associated with viral infection early stages. As the infection progresses, the number of double-membrane vesicles also increases (393).

Some viral proteins (CORE, NS2, NS5B, NS3, NS5A) directly benefit carcinogenesis through the induction of proliferation, angiogenesis, apoptosis, immune response, and inhibition of tumor suppressors (303). HCV can induce autophagosome formation and inhibit binding to lysosomes (311) (**Figure 7**). However, some *in vitro* studies have revealed that the virus can cause successful autophagic flux. This conflicting result may be explained by HCV probably inducing selective autophagy in some cellular organelles, such as the mitochondria and lipid deposits in cells (394).

Furthermore, the ATG16L complex is recruited into the membranous network, contributing to viral particle replication, and subsequent ATG12 removal suppresses viral RNA replication. Autophagy activation can counteract HCV infection, and the virus has developed different strategies to strengthen its persistence by temporarily regulating the autophagic process (395). The autophagic machinery is necessary for the translation of viral RNA in early stages of infection but becomes dispensable later. Once the replication process is complete, autophagy contributes to releasing viruses to the extracellular space, thus benefiting HCV transmission (300).

HCV infection has been found to induce autophagy through the direct interaction of viral proteins with autophagy effectors. In contrast, HCV has been shown to induce stress autophagy of the endoplasmic reticulum by inducing the three response pathways to misfolded proteins (p-ERK, ATF6 (activating transcription factor 6), IRE1) (396) (**Figure 7**). Reticulum stress produces calcium release, which disrupts mitochondrial activity and leads to ROS accumulation and damaged mitochondria. This leads to mitophagy activation through NS5A expression. This HCV non-structural protein causes an increase in LC3 levels and reduces p62 in host cells (397) (**Figure 7**). In patients with chronic HCV infections, mitophagy was observed to be induced due to PINK1 and Parkin’s translocation to the mitochondria outer surface. This mechanism correlates with oxidative phosphorylation dysregulation and mitochondria depletion, contributing to liver injury (398). HCV modulates the autophagic machinery in order to exist in the host cell. Like other viruses, it increases the formation of dysfunctional autophagic vesicles within which it replicates. Furthermore, in tumors associated with this virus chronic infections, high levels of mitophagy were presented (**Figure 7**).

## Hepatitis B Virus

HBV belongs to the *Hepdnaviridae* family and is responsible for developing acute and chronic viral hepatitis as well as long-term complications ranging from fibrosis and cirrhosis to cancer. Chronic infection progression is predominant in infected patients during the perinatal and infantile periods (399). Two billion people are estimated to be infected with HBV worldwide, and more than 350 million to be chronic carriers. Only 25% of infected patients develop liver neoplasms (300).

These viruses present circular double-stranded DNA of 3.2 kb in length, which has four open reading frames and has the peculiarity of forming an incomplete chain (400). The end of one strand is associated with viral DNA polymerase (401). Immediately after entering the cell, the virus undergoes reverse transcription within the nucleocapsid. Consequently, linear DNA is formed and secreted as virions or transported to the nucleus, integrating into the host genome to regulate tumor transformation (402).

HBV has a complex replication cycle and needs to encode at least seven viral proteins that regulate different processes in the host cell in order to complete it successfully (403). HBx (hepatitis B virus protein x) and SHB (small surface protein) expression is associated with autophagy regulation in several biological models (404) (**Figure 7**). SHB protein travels through the cytosol to the endoplasmic reticulum, where stress increases and generates massive autophagy induction. Signaling pathways initiated by stress sensors such as IRE1, p-ERK, and ATF6 help regulate cell homeostasis (405).

As for HBx, it is a multifunctional regulatory protein that may be involved in viral pathogenesis and carcinogenesis (406). Molecular studies have revealed that HBx generates strong autophagy induction by activating DAPK (death-associated protein kinase) and increasing BECN1 (407). However, in later stages of the autophagic mechanism, HBx can repress V-ATPase (vacuolar enzyme type H+), thus reducing lysosome and autolysosome acidification (408) (**Figure 7**). As they become less acidic, autolysosomes lose their degradative capacity as cytoplasmic debris increases in the cell, creating an inhospitable environment that contributes to carcinogenesis (408). The virus can also interfere with autophagic degradation through RAB7 (Ras-related protein), a small GTPase involved in autophagosome maturation and their fusion with lysosomes (409).

HBV-infected patient biopsies showed that viral persistence is correlated with the expression of mitophagy effectors, Parkin, and PINK (410). Furthermore, this mechanism is believed to be regulated by the AMPK/mTOR/ULK1 axis (411). Liver cells infected with HBV show that miR-155 expression contributes to viral replication and enhances autophagy induction (412). At least four miRNAs have been identified to inhibit viral replication in clinical samples from virus-infected patients, (let-7, miR-433, miR-345, miR-511) (413).

Liver cancer is a global concern due to its high resistance to chemotherapeutic drugs, which has been linked to exosome formation in transformed cells (414). These structures increase drug resistance by inducing chaperone-related signaling pathways and LAMP-2-dependent (type-2 lysosomal membrane protein) autophagy. Patients with liver tumors associated with primary HBV infection show greater tumor volume and greater pathogenicity. This suggests that the virus contributes to generating a more aggressive and resistant HCC phenotype (415).

Therefore, we can infer that HBV generates bimodal autophagy regulation: first by inducing the mechanism and then by inhibiting the formation of mature autolysosomes, thus contributing to viral replication and carcinogenesis (**Figure 7**).

## Papillomavirus and Human Polyomavirus

Papillomaviruses (HPV) and human polyomaviruses (PyV) were initially considered members of the same virus family due to their morphological similarity and genome organization, but have now been classified into separate families: *Papillomaviridae* and *Polyomaviridae*, respectively (416), both composed of viruses with a double-stranded circular DNA that encodes various regulatory and structural proteins, some of which have oncogenic properties (417).

Merkel cell polyomavirus (MCPyV) is implicated in developing a very aggressive skin cancer called Merkel carcinoma (418). Most findings about MCPyV oncogenic potential are associated with LT (large antigen T) and sT (small antigen T) expression (419, 420). LT has a J domain (heat shock protein binding domain), a retinoblastoma binding motif (RB, inhibits members of this protein family), and a C-terminus binding to helicase/ATPase domain (required for viral DNA replication) (418). LT’s oncogenic role is mainly associated to its high binding affinity to RB, which promotes the sequestration and inactivation of this tumor suppressor (421).

Regarding the sT antigen, it shares a 78 amino acid N-terminal region (includes the J domain) with LT and has a unique C-terminal domain. This antigen is considered to be the main MCPyV-induced oncogenesis regulator (422). LTs unique expression is sufficient to transform fibroblasts *in vitro* (423) and *in vivo* models (424). sT keeps the eukaryotic 4EB-P1 binding protein hyperphosphorylated and inactive, which leads to deregulation of cellular translation events and contributes to cell proliferation and transformation (422). Through these surface antigen expression, viruses modulate the autophagic machinery and contribute to immunosuppression and viral oncogenesis. In MCC tumors associated with MCPyV infection, sT and LT antigen expression suppresses autophagy through miR-375, miR-30a-3p, and miR30a-5p gene modulation. These microRNAs act on *atg7*, *p62*, and *bcn1* to inhibit autophagic initial stages. These events protect cancer cells from cell death (425).

Human papillomavirus (HPV) is the leading cause of cervical cancer (426). It is also associated with the development of non-melanoma skin cancers, cancer of the head, neck, oropharynx, and the development of various anogenital neoplasms (427). The viral genome is integrated into the host cell’s DNA and encodes the expression of early viral genes (E1 to E7) essential for cell replication, transcription, and transformation. These viruses can also regulate the expression of late genes L1 and L2, which encode viral capsid proteins (428).

Binding and internalizing the virus are processes closely related to manipulating the host cell autophagic machinery. The entry of HPV into cells is associated with autophagy suppression through mTOR activation (429). This event promotes mTORC1, 4EB-P1, and S6K1 (ribosomal protein kinase 1) phosphorylation and activation (430) and ULK1 inactivation. This -in turn- contributes to inhibiting the initial steps of the autophagic mechanism (431).

Once internalized in cells, HPV circulates through an endosomal compartment where capsid proteins are degraded within acidified endosomes, and the viral genome enters the nucleus (432). Here, HPV DNA is amplified and maintained as episomes in the epithelium basal cells by mechanisms mediated by E1 and E2 (433). These early expression proteins are involved in various cellular signaling mechanisms. In particular, E5, E6, and E7 oncoproteins modulate the host cell autophagic machinery. Keratinocytes with HPV-16 infection, followed by E5 expression, produce a decrease in LC3 levels and prevent p62 degradation. E5 interferes mechanically with the transcriptional activation of the autophagic machinery, regulating the expression of *bcn1*, *atg5*, *lc3*, *ulk1*, *atg4a*, and *atg7* (434).

Unlike E5, viral gene E6/E7 inhibits autolysosome formation by a mechanism involving p53 (435). HPV-16 and HPV-18 infections are associated with the development of squamous cell carcinomas of the head and neck, two neoplasms with a high incidence worldwide (436). In addition to regulating the autophagic machinery, viral proteins E6 and E7 contribute to p53 and p-Rb degradation of. All these events cause the activation of specific transcription factors that modulate cell fate (437). The information obtained so far reveals that autophagy inhibition promotes HPV life cycle and tumor progression.

## Conclusions

In recent decades, studying the different cellular functions associated with autophagy has kept specialized scientists alert. This degradative mechanism, used by mammalian cells to maintain cell homeostasis, also directly contributes to modulating the progression of various diseases, such as cancer. Cellular context is essential to determine the functionality of autophagy. In general, cells accumulate damage at disease initial stages, affecting critical points in cell cycle regulation and thus determining cell transformation. Cells can therefore activate autophagy to shrink the damaged organelles and counteract the stressful stimuli to which they are exposed and restore normal state. However, in the context of tumor progression and invasion, the situation may be different. Here, cells present multiple alterations in their signaling pathways, which develops an aggressive cell phenotype, active and uncontrolled division, and high metabolism. Tumor cells can then induce autophagy to generate a fuel supply to maintain tumor cell metabolism.

Furthermore, autophagy can regulate tumor angiogenesis and immunity to benefit disease progression. Before infection, cells can eliminate intracellular pathogens by enzymatic digestion in autophagic vesicles. However, many viruses have developed strategies that allow them to bypass host attack and achieve successful replication and permanence. The number of viruses that modulate the autophagic mechanism for their benefit is increasing. In general, these intracellular organisms regulate the autophagic machinery in a bimodal manner. Upon entering cells, they promote autophagosome formation and inhibit binding to lysosomes, thus losing their degradative capacity. Viruses can use these dysfunctional vesicles to replicate within them, while debris and damage accumulate in cell organelles due to the mechanism’s inefficiency. Other viruses, on the other hand, directly inhibit the autophagic machinery from preventing its degradation. Dysfunctional vesicle accumulation contributes to cell damage accumulation, which benefits cell transformation and tumor development over time. However, more studies are needed to clarify autophagy’s relation to viral infections and tumor development. A thorough understanding of these molecular mechanisms is crucial for developing new antiviral drugs and targeted oncogenic therapies.

## Author Contributions

AS received an invitation to contribute to the Research topic: New functions of autophagy pathways in cancer. This author designed and wrote the manuscript, MM created the schematic figures and provided critical reading, and OC contributed to the overall design, supervision of the work, and essential reading. All authors contributed to the article and approved the submitted version.

## Funding

This work was supported by NIH GRANT 1U54CA221208-01 (to OC), UBACYT GRANT 20020150100200BA (to OC), and PICT GRANT 2015-3436 (to OC). The funders had no role in study design, data collection, and analysis, decision to publish or preparation of the manuscript.

## Conflict of Interest

The authors declare that the research was conducted in the absence of any commercial or financial relationships that could be construed as a potential conflict of interest.

The handling Editor declared a shared affiliation, though no other collaboration, with the authors.

## References

[1] Hegyi G Vincze G Szasz A. On the Dynamic Equilibrium in Homeostasis. Open J Biophys (2012) 02(03):60–7. 10.4236/ojbiphy.2012.23009

[2] Greives TJ Dochtermann NA Stewart EC. Estimating heritable genetic contributions to innate immune and endocrine phenotypic correlations: A need to explore repeatability. Horm Behav (2017) 88:106–11. 10.1016/j.yhbeh.2016.11.015 27913139

[3] Bektas A Schurman SH Sen R Ferrucci L. Aging, inflammation and the environment. Exp Gerontol (2018) 105(October):10–8. 10.1016/j.exger.2017.12.015 PMC5909704 29275161

[4] Chatterjee N Walker GC. Mechanisms of DNA damage, repair, and mutagenesis. Environ Mol Mutagen (2017) 58(5):235–63. 10.1002/em.22087 PMC5474181 28485537

[5] Costas MA Rubio MF. Autofagia, una estrategia de supervivencia celular. Medicina (Buenos Aires) (2017) 77(4):314–20. 28825576

[6] Feng Y He D Yao Z Klionsky DJ. The machinery of macroautophagy. Cell Res (2014) 24(1):24–41. 10.1038/cr.2013.168 24366339 PMC3879710

[7] White E. The role for autophagy in cancer. J Clin Invest (2015) 125(1):42–6. 10.1172/JCI73941 PMC4382247 25654549

[8] Yu L Chen Y Tooze SA. Autophagy pathway: Cellular and molecular mechanisms. Autophagy (2018) 14(2):207–15. 10.1080/15548627.2017.1378838 PMC5902171 28933638

[9] Anding AL Baehrecke EH. Cleaning House: Selective Autophagy of Organelles. Dev Cell (2017) 41(1):10–22. 10.1016/j.devcel.2017.02.016 28399394 PMC5395098

[10] Noda NN Inagaki F. Mechanisms of Autophagy. Annu Rev Biophys (2015) 44(1):101–22. 10.1146/annurev-biophys-060414-034248 25747593

[11] Mizushima N. The ATG conjugation systems in autophagy. Curr Opin Cell Biol (2020) 63:1–10. 10.1016/j.ceb.2019.12.001 31901645

[12] Pyo J-O Nah J Jung Y-K. Molecules and their functions in autophagy. Exp Mol Med (2012) 44(2):73. 10.3858/emm.2012.44.2.029 22257882 PMC3296815

[13] Russell RC Yuan HX Guan K-L. Autophagy regulation by nutrient signaling. Cell Res (2014) 24(1):42–57. 10.1038/cr.2013.166 24343578 PMC3879708

[14] Ha J Guan K-L Kim J. AMPK and autophagy in glucose/glycogen metabolism. Mol Aspects Med (2015) 46:46–62. 10.1016/j.mam.2015.08.002 26297963

[15] Gui L Liu B LV G. Hypoxia induces autophagy in cardiomyocytes via a hypoxia-inducible factor 1-dependent mechanism. Exp Ther Med (2016) 11(6):2233–9. 10.3892/etm.2016.3190 PMC4887955 27284306

[16] Lai MC Chang CM Sun HS. Hypoxia Induces Autophagy through Translational Up-Regulation of Lysosomal Proteins in Human Colon Cancer Cells. Trajkovic V, ed. PloS One (2016) 11(4):e0153627. 10.1371/journal.pone.0153627 27078027 PMC4831676

[17] Chen A Sceneay J Gödde N Kinwel T Ham S Thompson EW. Intermittent hypoxia induces a metastatic phenotype in breast cancer. Oncogene (2018) 37(31):4214–25. 10.1038/s41388-018-0259-3 29713057

[18] Xia G Zhu T Li X Jin Y Zhou J Xiao J. ROS−mediated autophagy through the AMPK signaling pathway protects INS−1 cells from human islet amyloid polypeptide−induced cytotoxicity. Mol Med Rep (2018) 18(3):2744–52. 10.3892/mmr.2018.9248 PMC6102651 30015901

[19] Han X Zhong Z Kou J Zheng Y Liu Z Jiang Y. ROS Generated by Upconversion Nanoparticle-Mediated Photodynamic Therapy Induces Autophagy Via PI3K/AKT/ mTOR Signaling Pathway in M1 Peritoneal Macrophage. Cell Physiol Biochem (2018) 48(4):1616–27. 10.1159/000492283 30071509

[20] Yorimitsu T Klionsky DJ. Endoplasmic Reticulum Stress: A New Pathway to Induce Autophagy. Autophagy (2007) 3(2):160–2. 10.4161/auto.3653 17204854

[21] Nie T Yang S Ma H Zhang L Lu F Tao K. Regulation of ER stress-induced autophagy by GSK3β-TIP60-ULK1 pathway. Cell Death Dis (2016) 7(12):e2563–3. 10.1038/cddis.2016.423 PMC5260977 28032867

[22] Corona Velazquez AF Jackson WT. So Many Roads: the Multifaceted Regulation of Autophagy Induction. Mol Cell Biol (2018) 38(21):1–14. 10.1128/MCB.00303-18 PMC6189458 30126896

[23] Jang M Park R Kim H Namkoong S Jo D Huh YH. AMPK contributes to autophagosome maturation and lysosomal fusion. Sci Rep (2018) 8(1):12637. 10.1038/s41598-018-30977-7 30140075 PMC6107659

[24] Jung CH Ro SH Cao J Otto NM Kim DH. mTOR regulation of autophagy. FEBS Lett (2010) 584(7):1287–95. 10.1016/j.febslet.2010.01.017 PMC2846630 20083114

[25] Shao X Lai D Zhang L Xu H. Induction of Autophagy and Apoptosis via PI3K/AKT/TOR Pathways by Azadirachtin A in Spodoptera litura Cells. Sci Rep (2016) 6(December 2015):1–12. 10.1038/srep35482 27752103 PMC5067515

[26] Bourouis M Mondin M Dussert A Leopold P. Control of basal autophagy rate by vacuolar peduncle. Trajkovic V, ed. PloS One (2019) 14(2):e0209759. 10.1371/journal.pone.0209759 30735514 PMC6368412

[27] Rabanal-Ruiz Y Otten EG Krolchuk VI. mTORC1 as the main Gateway to autophagy. Essays Biochem (2017) 61:565–84. 10.1042/EBC20170027 PMC5869864 29233869

[28] Cork GK Thompson J Slawson C. Real Talk; The Inter-play Between the mTOR, AMPK, and Hexosamine Biosynthetic Pathways in Cell Signaling. Front Endocrinol (2018) 522(9):1–9. 10.1111/odi.12254 PMC6136272 30237786

[29] Klionsky DJ Schulman BA. Dynamic regulation of macroautophagy by distinctive ubiquitin-like proteins. Nat Struct Mol Biol (2014) 21(4):336–45. 10.1038/nsmb.2787 PMC4036234 24699082

[30] Zaza G Granata S Caletti C Signorini L Stallone G Lupo A. mTOR Inhibition Role in Cellular Mechanisms. Transplantation (2018) 102(>2S Suppl 1):S3–S16. 10.1097/TP.0000000000001806 29369970

[31] Li X He S Ma B. Autophagy and autophagy-related proteins in cancer. Mol Cancer (2020) 19(1):12. 10.1186/s12943-020-1138-4 31969156 PMC6975070

[32] Heras-Sandoval D Pérez-Rojas JM Pedraza-Chaverri J. Novel compounds for the modulation of mTOR and autophagy to treat neurodegenerative diseases. Cell Signal (2020) 65:109442. 10.1016/j.cellsig.2019.109442 31639492

[33] Qin L Wang Z Tao L Wang Y. ER stress negatively regulates AKT/TSC/mTOR pathway to enhance autophagy. Autophagy (2010) 6(2):239–47. 10.4161/auto.6.2.11062 20104019

[34] Di Nardo A Wertz MH Kwiatkowski E Tsai PT Leech JD Greene-Colozzi E. Neuronal Tsc1/2 complex controls autophagy through AMPK-dependent regulation of ULK1. Hum Mol Genet (2014) 23(14):3865–74. 10.1093/hmg/ddu101 PMC4065158 24599401

[35] Galluzzi L Pietrocola F Bravo-San Pedro JM Amaravadi RK Baehrecke EH Cecconi F. Autophagy in malignant transformation and cancer progression. EMBO J (2015) 34(7):856–80. 10.15252/embj.201490784 PMC4388596 25712477

[36] Janku F McConkey DJ Hong DS Kurzrock R. Autophagy as a target for anticancer therapy. Nat Rev Clin Oncol (2011) 8(9):528–39. 10.1038/nrclinonc.2011.71 21587219

[37] Kotani T Kirisako H Koizumi M Ohsumi Y Nakatogawa H. The Atg2-Atg18 complex tethers pre-autophagosomal membranes to the endoplasmic reticulum for autophagosome formation. Proc Natl Acad Sci (2018) 115(41):10363–8. 10.1073/pnas.1806727115 PMC6187169 30254161

[38] Ktistakis NT Tooze SA. Digesting the Expanding Mechanisms of Autophagy. Trends Cell Biol (2016) 26(8):624–35. 10.1016/j.tcb.2016.03.006 27050762

[39] Ge L Melville D Zhang M Schekman R. The ER–Golgi intermediate compartment is a key membrane source for the LC3 lipidation step of autophagosome biogenesis. Elife (2013) 2:1–23. 10.7554/eLife.00947 PMC3736544 23930225

[40] Wei Y Liu M Li X Liu J Li H. Origin of the Autophagosome Membrane in Mammals. BioMed Res Int (2018) 2018:1–9. 10.1155/2018/1012789 PMC6174804 30345294

[41] Lamb CA Yoshimori T Tooze SA. The autophagosome: origins unknown, biogenesis complex. Nat Rev Mol Cell Biol (2013) 14(12):759–74. 10.1038/nrm3696 24201109

[42] Ge L Baskaran S Schekman R Hurley JH. The protein-vesicle network of autophagy. Curr Opin Cell Biol (2014) 29(1):18–24. 10.1016/j.ceb.2014.02.005 24681112

[43] Decuypere JP Parys JB Bultynck G. Regulation of the Autophagic Bcl-2/Beclin 1 Interaction. Cells (2012) 1(3):284–312. 10.3390/cells1030284 24710477 PMC3901098

[44] Wen J Mai Z Zhao M Wang X Chen T. Full anti-apoptotic function of Bcl-XL complexed with Beclin-1 verified by live-cell FRET assays. Biochem Biophys Res Commun (2019) 511(3):700–4. 10.1016/j.bbrc.2019.02.107 30827509

[45] Nakajima S Aikawa C Nozawa T Minowa-Nozawa A Toh H Nakagawa I. Bcl-xL Affects Group A Streptococcus-Induced Autophagy Directly, by Inhibiting Fusion between Autophagosomes and Lysosomes, and Indirectly, by Inhibiting Bacterial Internalization via Interaction with Beclin 1-UVRAG. Trajkovic V, ed. PloS One (2017) 12(1):e0170138. 10.1371/journal.pone.0170138 28085926 PMC5235370

[46] Into T Inomata M Takayama E Takigawa T. Autophagy in regulation of Toll-like receptor signaling. Cell Signal (2012) 24(6):1150–62. 10.1016/j.cellsig.2012.01.020 22333395

[47] Marquez RT Xu L. Bcl-2:Beclin 1 complex: multiple, mechanisms regulating autophagy/apoptosis toggle switch. Am J Cancer Res (2012) 2(2):214–21. 2156-6976/ajcr0000098ajcr0000098 PMC3304572 22485198

[48] Grasso D Ropolo A Vaccaro MI. Autophagy in Cell Fate and Diseases. Cell Death - Autophagy, Apoptosis and Necrosis (2015) 1:3–26. 10.5772/61553

[49] Fujita N Itoh T Omori H Fukuda M Noda T Yoshimori T. The Atg16L Complex Specifies the Site of LC3 Lipidation for Membrane Biogenesis in Autophagy. Riezman H, ed. Mol Biol Cell (2008) 19(5):2092–100. 10.1091/mbc.e07-12-1257 PMC2366860 18321988

[50] Winer H Fraiberg M Abada A Dadosh T Tamim-Yecheskel BC Elazar Z. Autophagy differentially regulates TNF receptor Fn14 by distinct mammalian Atg8 proteins. Nat Commun (2018) 9(1):3744. 10.1038/s41467-018-06275-1 30218067 PMC6138730

[51] Schaaf MBE Keulers TG Vooijs MA Rouschop KMA. LC3/GABARAP family proteins: autophagy-(un)related functions. FASEB J (2016) 30(12):3961–78. 10.1096/fj.201600698R 27601442

[52] Brier LW Ge L Stjepanovic G Thelen AM Hurley J Schekman R. Regulation of LC3 Lipidation by the Autophagy-Specific Class III Phosphatidylinositol-3 Kinase Complex. Mol Biol Cell (2019) 30(9):1051–128. 10.1091/mbc.E18-11-0743 PMC6724508 30811270

[53] Moscat J Karin M Diaz-Meco MT. p62 in Cancer: Signaling Adaptor Beyond Autophagy. Cell (2016) 167(3):606–9. 10.1016/j.cell.2016.09.030 PMC5114003 27768885

[54] Lau A Wang XJ Zhao F Villeneuve NF Wu T Jiang T. A Noncanonical Mechanism of Nrf2 Activation by Autophagy Deficiency: Direct Interaction between Keap1 and p62. Mol Cell Biol (2010) 30(13):3275–85. 10.1128/MCB.00248-10 PMC2897585 20421418

[55] Valencia T Kim JY Abu-Baker S Moscat-Pardos J Ahn CS Reina-Campos M. Metabolic Reprogramming of Stromal Fibroblasts through p62-mTORC1 Signaling Promotes Inflammation and Tumorigenesis. Cancer Cell (2014) 26(1):121–35. 10.1016/j.ccr.2014.05.004 PMC4101061 25002027

[56] Zhao YG Zhang H. Autophagosome maturation: An epic journey from the ER to lysosomes. J Cell Biol (2019) 218(3):757–70. 10.1083/jcb.201810099 PMC6400552 30578282

[57] Parzych KR Klionsky DJ. An Overview of Autophagy: Morphology, Mechanism, and Regulation. Antioxid Redox Signal (2014) 20(3):460–73. 10.1089/ars.2013.5371 PMC3894687 23725295

[58] Jager S. Role for Rab7 in maturation of late autophagic vacuoles. J Cell Sci (2004) 117(20):4837–48. 10.1242/jcs.01370 15340014

[59] Itakura E Mizushima N. Characterization of autophagosome formation site by a hierarchical analysis of mammalian Atg proteins. Autophagy (2010) 6(6):764–76. 10.4161/auto.6.6.12709 PMC3321844 20639694

[60] Gatica D Lahiri V Klionsky DJ. Cargo recognition and degradation by selective autophagy. Nat Cell Biol (2018) 20(3):233–42. 10.1038/s41556-018-0037-z PMC6028034 29476151

[61] Jing K Lim K. Why is autophagy important in human diseases? Exp Mol Med (2012) 44(2):69. 10.3858/emm.2012.44.2.028 22257881 PMC3296814

[62] Yang Y Klionsky DJ. Autophagy and disease: unanswered questions. Cell Death Differ (2020) 27(3):858–71. 10.1038/s41418-019-0480-9 PMC7206137 31900427

[63] Zachari M Gudmundsson SR Li Z Manifava M Shah R Smith M. Selective Autophagy of Mitochondria on a Ubiquitin-Endoplasmic-Reticulum Platform. Dev Cell (2019) 50(5):627–43.e5. 10.1016/j.devcel.2019.06.016 31353311 PMC6739445

[64] Germain K Kim PK. Pexophagy: A Model for Selective Autophagy. Int J Mol Sci (2020) 21(2):578. 10.3390/ijms21020578 PMC7013971 31963200

[65] Cebollero E Reggiori F Kraft C. Reticulophagy and Ribophagy: Regulated Degradation of Protein Production Factories. Int J Cell Biol (2012) 2012:1–9. 10.1155/2012/182834 PMC3299282 22481944

[66] Nakatogawa H Mochida K. Reticulophagy and nucleophagy: New findings and unsolved issues. Autophagy (2015) 11(12):2377–8. 10.1080/15548627.2015.1106665 PMC4835146 26566146

[67] Singh R Cuervo AM. Lipophagy: Connecting Autophagy and Lipid Metabolism. Int J Cell Biol (2012) 2012:1–12. 10.1155/2012/282041 PMC3320019 22536247

[68] Bauckman KA Owusu-Boaitey N Mysorekar IU. Selective autophagy: Xenophagy. Methods (2015) 75:120–7. 10.1016/j.ymeth.2014.12.005 PMC4355331 25497060

[69] Lamark T Johansen T. Aggrephagy: Selective Disposal of Protein Aggregates by Macroautophagy. Int J Cell Biol (2012) 2012:1–21. 10.1155/2012/736905 PMC3320095 22518139

[70] Doherty J Baehrecke EH. Life, death and autophagy. Nat Cell Biol (2018) 20(10):1110–7. 10.1038/s41556-018-0201-5 PMC9721133 30224761

[71] Nah J Zablocki D Sadoshima J. Autosis. JACC Basic to Transl Sci (2020) 5(8):857–69. 10.1016/j.jacbts.2020.04.014 PMC7452304 32875173

[72] Swart C Du Toit A Loos B. Autophagy and the invisible line between life and death. Eur J Cell Biol (2016) 95(12):598–610. 10.1016/j.ejcb.2016.10.005 28340912

[73] Kriel J Loos B. The good, the bad and the autophagosome: exploring unanswered questions of autophagy-dependent cell death. Cell Death Differ (2019) 26(4):640–52. 10.1038/s41418-018-0267-4 PMC6460391 30659234

[74] Sridhar S Botbol Y MacIan F Cuervo AM. Autophagy and disease: Always two sides to a problem. J Pathol (2012) 226(2):255–73. 10.1002/path.3025 PMC3996449 21990109

[75] Santana-Codina N Mancias JD Kimmelman AC. The Role of Autophagy in Cancer. Annu Rev Cancer Biol (2017) 1(1):19–39. 10.1146/annurev-cancerbio-041816-122338 31119201 PMC6527373

[76] Hanahan D Weinberg RA. Hallmarks of Cancer: The Next Generation. Cell (2011) 144(5):646–74. 10.1016/j.cell.2011.02.013 21376230

[77] Choi KS. Autophagy and cancer. Exp Mol Med (2012) 44(2):109. 10.3858/emm.2012.44.2.033 22257886 PMC3296807

[78] Das CK Mandal M Kögel D. Pro-survival autophagy and cancer cell resistance to therapy. Cancer Metastasis Rev (2018) 37(4):749–66. 10.1007/s10555-018-9727-z 29536228

[79] Singh SS Vats S Chia AY-Q Tan TZ Deng S Ong MS. Dual role of autophagy in hallmarks of cancer. Oncogene (2018) 37(9):1142–58. 10.1038/s41388-017-0046-6 29255248

[80] Wang K Klionsky DJ. Mitochondria removal by autophagy. Autophagy (2011) 7(3):297–300. 10.4161/auto.7.3.14502 21252623 PMC3359476

[81] Liang XH Jackson S Seaman M Brown K Kempkes B Hibshoosh H. Induction of autophagy and inhibition of tumorigenesis by beclin 1. Nature (1999) 402(6762):672–6. 10.1038/45257 10604474

[82] Erlich S Mizrachy L Segev O Lindenboim L Zmira O Aldi-Harel S. Differential Interactions Between Beclin 1 and Bcl-2 Family Members. Autophagy (2007) 2007:3(6):561–8. 10.4161/auto.4713 17643073

[83] Kang R Zeh HJ Lotze MT Tang D. The Beclin 1 network regulates autophagy and apoptosis. Cell Death Differ (2011) 18(4):571–80. 10.1038/cdd.2010.191 PMC3131912 21311563

[84] Toton E Lisiak N Sawicka P Rybczynska M. Beclin-1 and its role as a target for anticancer therapy. J Physiol Pharmacol (2014) 65(4):459–67. 25179078

[85] Hill SM Wrobel L Rubinsztein DC. Post-translational modifications of Beclin 1 provide multiple strategies for autophagy regulation. Cell Death Differ (2019) 26(4):617–29. 10.1038/s41418-018-0254-9 PMC6460389 30546075

[86] Ávalos Y Canales J Bravo-Sagua R Criollo A Lavandero S Quest AFG. Tumor Suppression and Promotion by Autophagy. BioMed Res Int (2014) 2014:1–15. 10.1155/2014/603980 PMC4189854 25328887

[87] Eliopoulos AG Havaki S Gorgoulis VG. DNA Damage Response and Autophagy: A Meaningful Partnership. Front Genet (2016) 7:204(NOV). 10.3389/fgene.2016.00204 27917193 PMC5116470

[88] Rodriguez-Rocha H Garcia-Garcia A Panayiotidis MI Franco R. DNA damage and autophagy. Mutat Res Mol Mech Mutagen (2011) 711(1-2):158–66. 10.1016/j.mrfmmm.2011.03.007 PMC3105359 21419786

[89] Yun C Lee S. The Roles of Autophagy in Cancer. Int J Mol Sci (2018) 19(11):3466. 10.3390/ijms19113466 PMC6274804 30400561

[90] Takamura A Komatsu M Hara T Sakamoto A Kishi C Waguri S. Autophagy-deficient mice develop multiple liver tumors. Genes Dev (2011) 25(8):795–800. 10.1101/gad.2016211 21498569 PMC3078705

[91] Kimmelman AC. The dynamic nature of autophagy in cancer. Genes Dev (2011) 25(19):1999–2010. 10.1101/gad.17558811 21979913 PMC3197199

[92] Mariño G Salvador-Montoliu N Fueyo A Knecht E Mizushima N López-Otín C. Tissue-specific Autophagy Alterations and Increased Tumorigenesis in Mice Deficient in Atg4C/Autophagin-3. J Biol Chem (2007) 282(25):18573–83. 10.1074/jbc.M701194200 17442669

[93] Cianfanelli V Fuoco C Lorente M Salazar M Quondamatteo F Gherardini PF. AMBRA1 links autophagy to cell proliferation and tumorigenesis by promoting c-Myc dephosphorylation and degradation. Nat Cell Biol (2015) 17(1):20–30. 10.1038/ncb3072 25438055 PMC4976803

[94] Maiuri MC Galluzzi L Morselli E Kepp O Malik SA Kroemer G. Autophagy regulation by p53. Curr Opin Cell Biol (2010) 22(2):181–5. 10.1016/j.ceb.2009.12.001 20044243

[95] Luo Z Zang M Guo W. AMPK as a metabolic tumor suppressor: control of metabolism and cell growth. Futur Oncol (2010) 6(3):457–70. 10.2217/fon.09.174 PMC2854547 20222801

[96] Tasdemir E Maiuri MC Morselli E Criollo A D’Amelio M Djavaheri-Mergny M. A dual role of p53 in the control of autophagy. Autophagy (2008) 4(6):810–4. 10.4161/auto.6486 18604159

[97] Mrakovcic M Fröhlich LF. p53-Mediated Molecular Control of Autophagy in Tumor Cells. Biomolecules (2018) 8(2):14. 10.3390/biom8020014 PMC6022997 29561758

[98] Rosenfeldt MT O’Prey J Morton JP Nixon C MacKay G Mrowinska A. p53 status determines the role of autophagy in pancreatic tumour development. Nature (2013) 504(7479):296–300. 10.1038/nature12865 24305049

[99] White E. Autophagy and p53. Cold Spring Harb Perspect Med (2016) 6(4):a026120. 10.1101/cshperspect.a026120 27037419 PMC4817743

[100] Kenzelmann Broz D Spano Mello S Bieging KT Jiang D Dusek RL Brady CA. Global genomic profiling reveals an extensive p53-regulated autophagy program contributing to key p53 responses. Genes Dev (2013) 27(9):1016–31. 10.1101/gad.212282.112 PMC3656320 23651856

[101] Eby KG Rosenbluth JM Mays DJ Marshall CB Barton CE Sinha S. ISG20L1 is a p53 family target gene that modulates genotoxic stress-induced autophagy. Mol Cancer (2010) 9(1):95. 10.1186/1476-4598-9-95 20429933 PMC2873442

[102] Mowers EE Sharifi MN Macleod KF. Autophagy in cancer metastasis. Oncogene (2017) 36(12):1619–30. 10.1038/onc.2016.333 PMC5337449 27593926

[103] Yang ZJ Chee CE Huang S Sinicrope FA. The Role of Autophagy in Cancer: Therapeutic Implications. Mol Cancer Ther (2011) 10(9):1533–41. 10.1158/1535-7163.MCT-11-0047 PMC3170456 21878654

[104] Simon T Gagliano T Giamas G. Direct Effects of Anti-Angiogenic Therapies on Tumor Cells: VEGF Signaling. Trends Mol Med (2017) 23(3):282–92. 10.1016/j.molmed.2017.01.002 28162910

[105] Salabei JK Cummins TD Singh M Jones SP Bhatnagar A Hill BG. PDGF-mediated autophagy regulates vascular smooth muscle cell phenotype and resistance to oxidative stress. Biochem J (2013) 451(3):375–88. 10.1042/BJ20121344 PMC4040966 23421427

[106] Bharath LP Cho JM Park S-K Ruan T Li Y Mueller R. Endothelial Cell Autophagy Maintains Shear Stress–Induced Nitric Oxide Generation via Glycolysis-Dependent Purinergic Signaling to Endothelial Nitric Oxide Synthase. Arterioscler Thromb Vasc Biol (2017) 37(9):1646–56. 10.1161/ATVBAHA.117.309510 PMC5693355 28684613

[107] Qu X. Promotion of tumorigenesis by heterozygous disruption of the beclin 1 autophagy gene. J Clin Invest (2003) 112(12):1809–20. 10.1172/JCI200320039 PMC297002 14638851

[108] Das CK Banerjee I Mandal M. Pro-survival autophagy: An emerging candidate of tumor progression through maintaining hallmarks of cancer. Semin Cancer Biol (2019) 66:59–74. 10.1016/j.semcancer.2019.08.020 31430557

[109] Bykov VJN Eriksson SE Bianchi J Wiman KG. Targeting mutant p53 for efficient cancer therapy. Nat Rev Cancer (2018) 18(2):89–102. 10.1038/nrc.2017.109 29242642

[110] Huo Y Cai H Teplova I Bowman-Colin C Chen G Price S. Autophagy Opposes p53-Mediated Tumor Barrier to Facilitate Tumorigenesis in a Model of PALB2-Associated Hereditary Breast Cancer. Cancer Discov (2013) 3(8):894–907. 10.1158/2159-8290.CD-13-0011 23650262 PMC3740014

[111] Moscat J Diaz-Meco MT. p62: a versatile multitasker takes on cancer. Trends Biochem Sci (2012) 37(6):230–6. 10.1016/j.tibs.2012.02.008 PMC3531712 22424619

[112] Li L Shen C Nakamura E Ando K Signoretti S Beroukhim R. SQSTM1 Is a Pathogenic Target of 5q Copy Number Gains in Kidney Cancer. Cancer Cell (2013) 24(6):738–50. 10.1016/j.ccr.2013.10.025 PMC3910168 24332042

[113] Umemura A He F Taniguchi K Nakagawa H Yamachika S font-Burgada J. p62, Upregulated during Preneoplasia, Induces Hepatocellular Carcinogenesis by Maintaining Survival of Stressed HCC-Initiating Cells. Cancer Cell (2016) 29(6):935–48. 10.1016/j.ccell.2016.04.006 PMC4907799 27211490

[114] Zhang J Yang S Xu B Wang T Zheng Y Liu F. p62 functions as an oncogene in colorectal cancer through inhibiting apoptosis and promoting cell proliferation by interacting with the vitamin D receptor. Cell Prolif (2019) 52(3):e12585. 10.1111/cpr.12585 30793399 PMC6536406

[115] Ren F Shu G Liu G Liu D Zhou J Yuan L. Knockdown of p62/sequestosome 1 attenuates autophagy and inhibits colorectal cancer cell growth. Mol Cell Biochem (2014) 385(1-2):95–102. 10.1007/s11010-013-1818-0 24065390

[116] Schmukler E Kloog Y Pinkas-Kramarski R. Ras and autophagy in cancer development and therapy. Oncotarget (2014) 5(3):577–86. 10.18632/oncotarget.1775 PMC3996671 24583697

[117] Lock R Kenific CM Leidal AM Salas E Debnath J. Autophagy-dependent production of secreted factors facilitates oncogenic RAS-Driven invasion. Cancer Discov (2014) 4(4):466–79. 10.1158/2159-8290.CD-13-0841 PMC3980002 24513958

[118] Guo JY Karsli-Uzunbas G Mathew R Aisner SC Kamphorst JJ Strohecker AM. Autophagy suppresses progression of K-ras-induced lung tumors to oncocytomas and maintains lipid homeostasis. Genes Dev (2013) 27(13):1447–61. 10.1101/gad.219642.113 PMC3713426 23824538

[119] Ling J Kang Y Zhao R Xia Q Lee D-F Chang Z. KrasG12D-Induced IKK2/β/NF-κB Activation by IL-1α and p62 Feedforward Loops Is Required for Development of Pancreatic Ductal Adenocarcinoma. Cancer Cell (2012) 21(1):105–20. 10.1016/j.ccr.2011.12.006 PMC3360958 22264792

[120] Sosa MS Bragado P Debnath J Aguirre-Ghiso JA. Regulation of Tumor Cell Dormancy by Tissue Microenvironments and Autophagy. In: Enderling H Almog N Hlatky L, editors. Advances in Experimental Medicine and Biology, vol. 734 . New York, NY: Springer New York (2013). p. 73–89. 10.1007/978-1-4614-1445-2_5 PMC3651695 23143976

[121] Lorin S Hamaï A Mehrpour M Codogno P. Autophagy regulation and its role in cancer. Semin Cancer Biol (2013) 23(5):361–79. 10.1016/j.semcancer.2013.06.007 23811268

[122] Paoli P Giannoni E Chiarugi P. Anoikis molecular pathways and its role in cancer progression. Biochim Biophys Acta - Mol Cell Res (2013) 1833(12):3481–98. 10.1016/j.bbamcr.2013.06.026 23830918

[123] Buchheit CL Weigel KJ Schafer ZT. Cancer cell survival during detachment from the ECM: multiple barriers to tumour progression. Nat Rev Cancer (2014) 14(9):632–41. 10.1038/nrc3789 25098270

[124] Zhi X Zhong Q. Autophagy in cancer. F1000Prime Rep (2015) 7(February):1–12. 10.12703/P7-18 PMC4338832 25750736

[125] Swartz MA Iida N Roberts EW Sangaletti S Wong MH Yull FE. Tumor Microenvironment Complexity: Emerging Roles in Cancer Therapy: Figure 1. Cancer Res (2012) 72(10):2473–80. 10.1158/0008-5472.CAN-12-0122 PMC3653596 22414581

[126] Camuzard O Santucci-Darmanin S Carle GF Pierrefite-Carle V. Autophagy in the crosstalk between tumor and microenvironment. Cancer Lett (2020) 490:143–53. 10.1016/j.canlet.2020.06.015 32634449

[127] Roma-Rodrigues C Mendes R Baptista P Fernandes A. Targeting Tumor Microenvironment for Cancer Therapy. Int J Mol Sci (2019) 20(4):840. 10.3390/ijms20040840 PMC6413095 30781344

[128] Ye J Wu D Wu P Chen Z Huang J. The cancer stem cell niche: cross talk between cancer stem cells and their microenvironment. Tumor Biol (2014) 35(5):3945–51. 10.1007/s13277-013-1561-x 24420150

[129] Kalluri R Zeisberg M. Fibroblasts in cancer. Nat Rev Cancer (2006) 6(5):392–401. 10.1038/nrc1877 16572188

[130] Folkerts H Hilgendorf S Vellenga E Bremer E Wiersma VR. The multifaceted role of autophagy in cancer and the microenvironment. Med Res Rev (2019) 39(2):517–60. 10.1002/med.21531 PMC6585651 30302772

[131] Guo JY Teng X Laddha SV Ma S Van Nostrand SC Yang Y. Autophagy provides metabolic substrates to maintain energy charge and nucleotide pools in Ras-driven lung cancer cells. Genes Dev (2016) 30(15):1704–17. 10.1101/gad.283416.116 PMC5002976 27516533

[132] Ngabire D Kim G-D. Autophagy and Inflammatory Response in the Tumor Microenvironment. Int J Mol Sci (2017) 18(9):2016. 10.3390/ijms18092016 PMC5618664 28930154

[133] Hui L Chen Y. Tumor microenvironment: Sanctuary of the devil. Cancer Lett (2015) 368(1):7–13. 10.1016/j.canlet.2015.07.039 26276713

[134] Anthony BA Link DC. Regulation of hematopoietic stem cells by bone marrow stromal cells. Trends Immunol (2014) 35(1):32–7. 10.1016/j.it.2013.10.002 PMC3947365 24210164

[135] Guan J-L Simon AK Prescott M Menendez JA Liu F Wang F. Autophagy in stem cells. Autophagy (2013) 9(6):830–49. 10.4161/auto.24132 PMC3672294 23486312

[136] Ito K Ito K. Hematopoietic stem cell fate through metabolic control. Exp Hematol (2018) 64:1–11. 10.1016/j.exphem.2018.05.005 29807063 PMC6084457

[137] Salemi S Yousefi S Constantinescu MA Fey MF Simon H-U. Autophagy is required for self-renewal and differentiation of adult human stem cells. Cell Res (2012) 22(2):432–5. 10.1038/cr.2011.200 PMC3271583 22184008

[138] Liu Q Chen L Atkinson JM Claxton DF Wang H-G. Atg5-dependent autophagy contributes to the development of acute myeloid leukemia in an MLL-AF9-driven mouse model. Cell Death Dis (2016) 7(9):e2361–1. 10.1038/cddis.2016.264 PMC5059867 27607576

[139] Jacquel A Obba S Boyer L Dufies M Robert G Gounon P. Autophagy is required for CSF-1–induced macrophagic differentiation and acquisition of phagocytic functions. Blood (2012) 119(19):4527–31. 10.1182/blood-2011-11-392167 22452982

[140] Jacquel A Benikhlef N Paggetti J Lalaoui N Guery L Dufour EK. Colony-stimulating factor-1–induced oscillations in phosphatidylinositol-3 kinase/AKT are required for caspase activation in monocytes undergoing differentiation into macrophages. Blood (2009) 114(17):3633–41. 10.1182/blood-2009-03-208843 19721010

[141] Zhang Y Morgan MJ Chen K Choksi S Liu Z. Induction of autophagy is essential for monocyte-macrophage differentiation. Blood (2012) 119(12):2895–905. 10.1182/blood-2011-08-372383 PMC3327464 22223827

[142] Modi J Menzie-Suderam J Xu H Trujillo P Medley K Marshall ML. Mode of action of granulocyte-colony stimulating factor (G-CSF) as a novel therapy for stroke in a mouse model. J BioMed Sci (2020) 27(1):19. 10.1186/s12929-019-0597-7 31907023 PMC6943893

[143] Martinez-Outschoorn UE Whitaker-Menezes D Lin Z Flomenberg N Howell A Pestell RG. Cytokine production and inflammation drive autophagy in the tumor microenvironment. Cell Cycle (2011) 10(11):1784–93. 10.4161/cc.10.11.15674 PMC3142462 21566463

[144] Goswami KK Ghosh T Ghosh S Sarkar M Bose A Baral R. Tumor promoting role of anti-tumor macrophages in tumor microenvironment. Cell Immunol (2017) 316(April):1–10. 10.1016/j.cellimm.2017.04.005 28433198

[145] Martinez-Outschoorn UE Trimmer C Lin Z Whitaker-Menezes D Chiavarina B Zhou J. Autophagy in cancer associated fibroblasts promotes tumor cell survival. Cell Cycle (2010) 9(17):3515–33. 10.4161/cc.9.17.12928 PMC3047617 20855962

[146] Capparelli C Guido C Whitaker-Menezes D Bonuccelli G Balliet R Pestell TG. Autophagy and senescence in cancer-associated fibroblasts metabolically supports tumor growth and metastasis, via glycolysis and ketone production. Cell Cycle (2012) 11(12):2285–302. 10.4161/cc.20718 PMC3383590 22684298

[147] Grasso D Garcia MN Iovanna JL. Autophagy in Pancreatic Cancer. Int J Cell Biol (2012) 2012:1–7. 10.1155/2012/760498 PMC3265076 22291707

[148] Mowers EE Sharifi MN Macleod KF. Functions of autophagy in the tumor microenvironment and cancer metastasis. FEBS J (2018) 285(10):1751–66. 10.1111/febs.14388 PMC5992019 29356327

[149] Endo S Nakata K Ohuchida K Takesue S Nakayama H Abe T. Autophagy Is Required for Activation of Pancreatic Stellate Cells, Associated With Pancreatic Cancer Progression and Promotes Growth of Pancreatic Tumors in Mice. Gastroenterology (2017) 152(6):1492–1506.e24. 10.1053/j.gastro.2017.01.010 28126348

[150] Schaaf MB Houbaert D Meçe O Agostinis P. Autophagy in endothelial cells and tumor angiogenesis. Cell Death Differ (2019) 26(4):665–79. 10.1038/s41418-019-0287-8 PMC6460396 30692642

[151] Welti J Loges S Dimmeler S Carmeliet P. Recent molecular discoveries in angiogenesis and antiangiogenic therapies in cancer. J Clin Invest (2013) 123(8):3190–200. 10.1172/JCI70212 PMC3726176 23908119

[152] Carmeliet P Jain RK. Angiogenesis in cancer and other diseases. Nature (2000) 407(6801):249–57. 10.1038/35025220 11001068

[153] Filippi I Saltarella I Aldinucci C Carraro F Ria R Vacca A. Different Adaptive Responses to Hypoxia in Normal and Multiple Myeloma Endothelial Cells. Cell Physiol Biochem (2018) 46(1):203–12. 10.1159/000488423 29587264

[154] Chandra A Rick J Yagnik G Aghi MK. Autophagy as a mechanism for anti-angiogenic therapy resistance. Semin Cancer Biol (2020) 66(January):75–88. 10.1016/j.semcancer.2019.08.031 31472232 PMC7047534

[155] Maes H Olmeda D Soengas MS Agostinis P. Vesicular trafficking mechanisms in endothelial cells as modulators of the tumor vasculature and targets of antiangiogenic therapies. FEBS J (2016) 283(1):25–38. 10.1111/febs.13545 26443003

[156] Maes H Rubio N Garg AD Agostinis P. Autophagy: shaping the tumor microenvironment and therapeutic response. Trends Mol Med (2013) 19(7):428–46. 10.1016/j.molmed.2013.04.005 23714574

[157] Ma Y Galluzzi L Zitvogel L Kroemer G. Autophagy and Cellular Immune Responses. Immunity (2013) 39(2):211–27. 10.1016/j.immuni.2013.07.017 23973220

[158] Caron E Vincent K Fortier M Laverdure J-P Bramoullé A Hardy M-P. The MHC I immunopeptidome conveys to the cell surface an integrative view of cellular regulation. Mol Syst Biol (2011) 7(1):533. 10.1038/msb.2011.68 21952136 PMC3202804

[159] Michaud M Martins I Sukkurwala AQ Adjemian S Ma Y Pellegatti P. Autophagy-Dependent Anticancer Immune Responses Induced by Chemotherapeutic Agents in Mice. Science (80- ) (2011) 334(6062):1573–7. 10.1126/science.1208347 22174255

[160] Wildenberg ME Vos ACW Wolfkamp SCS Duijvestein M Verhaar AP Te Velde AA. Autophagy Attenuates the Adaptive Immune Response by Destabilizing the Immunologic Synapse. Gastroenterology (2012) 142(7):1493–503.e6. 10.1053/j.gastro.2012.02.034 22370477

[161] Jia W He Y-W. Temporal Regulation of Intracellular Organelle Homeostasis in T Lymphocytes by Autophagy. J Immunol (2011) 186(9):5313–22. 10.4049/jimmunol.1002404 21421856

[162] Fiegl D Kägebein D Liebler-Tenorio EM Weisser T Sens M Gutjahr M. Amphisomal Route of MHC Class I Cross-Presentation in Bacteria-Infected Dendritic Cells. J Immunol (2013) 190(6):2791–806. 10.4049/jimmunol.1202741 23418629

[163] Vesely MD Kershaw MH Schreiber RD Smyth MJ. Natural Innate and Adaptive Immunity to Cancer. Annu Rev Immunol (2011) 29(1):235–71. 10.1146/annurev-immunol-031210-101324 21219185

[164] Lapaquette P Guzzo J Bretillon L Bringer M-A. Cellular and Molecular Connections between Autophagy and Inflammation. Mediators Inflamm (2015) 2015:1–13. 10.1155/2015/398483 PMC4499609 26221063

[165] Vidya MK Kumar VG Sejian V Bagath M Krishnan G Bhatta R. Toll-like receptors: Significance, ligands, signaling pathways, and functions in mammals. Int Rev Immunol (2018) 37(1):20–36. 10.1080/08830185.2017.1380200 29028369

[166] Hontelez S Sanecka A Netea MG van Spriel AB Adema GJ. Molecular view on PRR cross-talk in antifungal immunity. Cell Microbiol (2012) 14(4):467–74. 10.1111/j.1462-5822.2012.01748.x 22233321

[167] Sharma V Verma S Seranova E Sarkar S Kumar D. Selective Autophagy and Xenophagy in Infection and Disease. Front Cell Dev Biol (2018) 6:147(NOV). 10.3389/fcell.2018.00147 30483501 PMC6243101

[168] Delgado MA Elmaoued RA Davis AS Kyei G Deretic V. Toll-like receptors control autophagy. EMBO J (2008) 27(7):1110–21. 10.1038/emboj.2008.31 PMC2323261 18337753

[169] Shi C-S Kehrl JH. MyD88 and Trif Target Beclin 1 to Trigger Autophagy in Macrophages. J Biol Chem (2008) 283(48):33175–82. 10.1074/jbc.M804478200 PMC2586260 18772134

[170] Weichhart T Hengstschläger M Linke M. Regulation of innate immune cell function by mTOR. Nat Rev Immunol (2015) 15(10):599–614. 10.1038/nri3901 26403194 PMC6095456

[171] Hamon MA Quintin J. Innate immune memory in mammals. Semin Immunol (2016) 28(4):351–8. 10.1016/j.smim.2016.05.003 27264334

[172] Mah LY Ryan KM. Autophagy and Cancer. Cold Spring Harb Perspect Biol (2012) 4(1):a008821–a008821. 10.1101/cshperspect.a008821 22166310 PMC3249624

[173] Wieczorek M Abualrous ET Sticht J Álvalo-Benito M Stolzenberg S Noé F. Major Histocompatibility Complex (MHC) Class I and MHC Class II Proteins: Conformational Plasticity in Antigen Presentation. Front Immunol (2017) 8:292(MAR). 10.3389/fimmu.2017.00292 28367149 PMC5355494

[174] Li B Lei Z Lichty BD Li D Zhang G-M Feng Z-H. Autophagy facilitates major histocompatibility complex class I expression induced by IFN-γ in B16 melanoma cells. Cancer Immunol Immunother (2010) 59(2):313–21. 10.1007/s00262-009-0752-1 PMC11029913 19680649

[175] Crotzer VL Blum JS. Autophagy and adaptive immunity. Immunology (2010) 131(1):no–o. 10.1111/j.1365-2567.2010.03321.x PMC2966753 20586810

[176] You L Jin S Zhu L Qian W. Autophagy, autophagy-associated adaptive immune responses and its role in hematologic malignancies. Oncotarget (2017) 8(7):12374–88. 10.18632/oncotarget.13583 PMC5355352 27902471

[177] Puleston DJ Simon AK. Autophagy in the immune system. Immunology (2014) 141(1):1–8. 10.1111/imm.12165 23991647 PMC3893844

[178] Mortensen M Ferguson DJP Edelmann M Kessler B Morten KJ Komatsu M. Loss of autophagy in erythroid cells leads to defective removal of mitochondria and severe anemia in vivo. Proc Natl Acad Sci (2010) 107(2):832–7. 10.1073/pnas.0913170107 PMC2818953 20080761

[179] Miller BC Zhao Z Stephenson LM Cadwell K Pua HH Lee HK. The autophagy gene ATG5 plays an essential role in B lymphocyte development. Autophagy (2008) 4(3):309–14. 10.4161/auto.5474 18188005

[180] Rybstein MD Bravo-San Pedro JM Kroemer G Galluzzi L. The autophagic network and cancer. Nat Cell Biol (2018) 20(3):243–51. 10.1038/s41556-018-0042-2 29476153

[181] Monkkonen T Debnath J. Inflammatory signaling cascades and autophagy in cancer. Autophagy (2018) 14(2):190–8. 10.1080/15548627.2017.1345412 PMC5902219 28813180

[182] Nakamura K Smyth MJ. Targeting cancer-related inflammation in the era of immunotherapy. Immunol Cell Biol (2017) 95(4):325–32. 10.1038/icb.2016.126 27999432

[183] Deretic V Jiang S Dupont N. Autophagy intersections with conventional and unconventional secretion in tissue development, remodeling and inflammation. Trends Cell Biol (2012) 22(8):397–406. 10.1016/j.tcb.2012.04.008 22677446 PMC3408825

[184] Nawas AF Mistry R Narayanan S Thomas-Jardin SE Ramachandran J Ravichandran J. IL-1 induces p62/SQSTM1 and autophagy in ERα + /PR + BCa cell lines concomitant with ERα and PR repression, conferring an ERα – /PR – BCa-like phenotype. J Cell Biochem (2019) 120(2):1477–91. 10.1002/jcb.27340 PMC6465183 30324661

[185] Li TT Zhu D Mou T Guo Z Pu JL Chen QS. IL-37 induces autophagy in hepatocellular carcinoma cells by inhibiting the PI3K/AKT/mTOR pathway. Mol Immunol (2017) 87(1):132–40. 10.1016/j.molimm.2017.04.010 28433890

[186] Chio IIC Tuveson DA. ROS in Cancer: The Burning Question. Trends Mol Med (2017) 23(5):411–29. 10.1016/j.molmed.2017.03.004 PMC5462452 28427863

[187] Hoesel B Schmid JA. The complexity of NF-κB signaling in inflammation and cancer. Mol Cancer (2013) 12(1):86. 10.1186/1476-4598-12-86 23915189 PMC3750319

[188] Duran A Linares JF Galvez AS Wikenheiser K Flores JM Diaz-Meco MT. The Signaling Adaptor p62 Is an Important NF-κB Mediator in Tumorigenesis. Cancer Cell (2008) 13(4):343–54. 10.1016/j.ccr.2008.02.001 18394557

[189] Schwartz L Supuran C Alfarouk K. The Warburg Effect and the Hallmarks of Cancer. Anticancer Agents Med Chem (2017) 17(2):164–70. 10.2174/1871520616666161031143301 27804847

[190] Pavlides S Tsirigos A Migneco G Whitaker-Menezes D chiavarina B Flomenberg N. The autophagic tumor stroma model of cancer. Cell Cycle (2010) 9(17):3485–505. 10.4161/cc.9.17.12721 PMC3047615 20861672

[191] Xing Y Zhao S Zhou BP Mi J. Metabolic reprogramming of the tumour microenvironment. FEBS J (2015) 282(20):3892–8. 10.1111/febs.13402 26255648

[192] Wei H Wei S Gan B Peng X Zou W Guan J-L. Suppression of autophagy by FIP200 deletion inhibits mammary tumorigenesis. Genes Dev (2011) 25(14):1510–27. 10.1101/gad.2051011 PMC3143941 21764854

[193] Karvela M Baquero P Kuntz EM Mukhopadhyay A Mitchell R Allan EK. ATG7 regulates energy metabolism, differentiation and survival of Philadelphia-chromosome-positive cells. Autophagy (2016) 12(6):936–48. 10.1080/15548627.2016.1162359 PMC4922442 27168493

[194] Correia M Pinheiro P Batista R Soares P Sobrinho-Simões M Máximo V. Etiopathogenesis of oncocytomas. Semin Cancer Biol (2017) 47:82–94. 10.1016/j.semcancer.2017.06.014 28687249

[195] Amaravadi R Kimmelman AC White E. Recent insights into the function of autophagy in cancer. Genes Dev (2016) 30(17):1913–30. 10.1101/gad.287524.116 PMC5066235 27664235

[196] Vara-Perez M Felipe-Abrio B Agostinis P. Mitophagy in Cancer: A Tale of Adaptation. Cells (2019) 8(5):493. 10.3390/cells8050493 PMC6562743 31121959

[197] Semenza GL. Hypoxia-inducible factor 1: Regulator of mitochondrial metabolism and mediator of ischemic preconditioning. Biochim Biophys Acta - Mol Cell Res (2011) 1813(7):1263–8. 10.1016/j.bbamcr.2010.08.006 PMC3010308 20732359

[198] Sowter HM Ratcliffe PJ Watson P Greenberg AH Harris AL. HIF-1-dependent regulation of hypoxic induction of the cell death factors BNIP3 and NIX in human tumors. Cancer Res (2001) 61(18):6669–73. 11559532

[199] Lyons A Coleman M Riis S Favre C O’Flanagan CH Zhdanov AV. Insulin-like growth factor 1 signaling is essential for mitochondrial biogenesis and mitophagy in cancer cells. J Biol Chem (2017) 292(41):16983–98. 10.1074/jbc.M117.792838 PMC5641874 28821609

[200] Agnihotri S Golbourn B Huang X Remke M Younger S Cairns RA. PINK1 Is a Negative Regulator of Growth and the Warburg Effect in Glioblastoma. Cancer Res (2016) 76(16):4708–19. 10.1158/0008-5472.CAN-15-3079 27325644

[201] Chang HW Kim MR Lee HJ Lee HJ Kim GC Lee YS. p53/BNIP3-dependent mitophagy limits glycolytic shift in radioresistant cancer. Oncogene (2019) 38(19):3729–42. 10.1038/s41388-019-0697-6 30664690

[202] Hoshino A Ariyoshi M Okawa Y Kaimoto S Uchihashi M Fukai K. Inhibition of p53 preserves Parkin-mediated mitophagy and pancreatic -cell function in diabetes. Proc Natl Acad Sci (2014) 111(8):3116–21. 10.1073/pnas.1318951111 PMC3939874 24516131

[203] Mariño G Pietrocola F Eisenberg T Kong Y Malik SA Andryushkova A. Regulation of Autophagy by Cytosolic Acetyl-Coenzyme A. Mol Cell (2014) 53(5):710–25. 10.1016/j.molcel.2014.01.016 24560926

[204] Lee JV Berry CT Kim K Sen P Kim T Carrer A. Acetyl-CoA promotes glioblastoma cell adhesion and migration through Ca 2+ –NFAT signaling. Genes Dev (2018) 32(7-8):497–511. 10.1101/gad.311027.117 29674394 PMC5959234

[205] Schnittert J Bansal R Prakash J. Targeting Pancreatic Stellate Cells in Cancer. Trends Cancer (2019) 5(2):128–42. 10.1016/j.trecan.2019.01.001 30755305

[206] Sousa CM Biancur DE Wang X Halbrook CJ Sherman MH Zhang L. Pancreatic stellate cells support tumour metabolism through autophagic alanine secretion. Nature (2016) 536(7617):479–83. 10.1038/nature19084 PMC5228623 27509858

[207] Kim J Gao P Liu Y-C Semenza GL Dang CV. Hypoxia-Inducible Factor 1 and Dysregulated c-Myc Cooperatively Induce Vascular Endothelial Growth Factor and Metabolic Switches Hexokinase 2 and Pyruvate Dehydrogenase Kinase 1. Mol Cell Biol (2007) 27(21):7381–93. 10.1128/MCB.00440-07 PMC2169056 17785433

[208] Nagao A Kobayashi M Koyasu S Chow CCT Harada H. HIF-1-Dependent Reprogramming of Glucose Metabolic Pathway of Cancer Cells and Its Therapeutic Significance. Int J Mol Sci (2019) 20(2):238. 10.3390/ijms20020238 PMC6359724 30634433

[209] Dang CV. The Interplay Between MYC and HIF in the Warburg Effect. Ernst Schering Found Symp Proc (2008) 8:35–53. 10.1007/2789_2008_088 18811052

[210] Xiong J Wang L Fei X-C Jiang X-F Zheng Z Zhao Y. MYC is a positive regulator of choline metabolism and impedes mitophagy-dependent necroptosis in diffuse large B-cell lymphoma. Blood Cancer J (2017) 7(7):e582–2. 10.1038/bcj.2017.61 PMC5549253 28686226

[211] Cianfanelli V Fuoco C Lorente M Salazar M Quondamatteo F Gherardini PF. Erratum: Corrigendum: AMBRA1 links autophagy to cell proliferation and tumorigenesis by promoting c-Myc dephosphorylation and degradation. Nat Cell Biol (2015) 17(5):706–6. 10.1038/ncb3171 25925585

[212] GARGINI R GARCÍA-ESCUDERO V IZQUIERDO M WANDOSELL F. Oncogene-mediated tumor transformation sensitizes cells to autophagy induction. Oncol Rep (2016) 35(6):3689–95. 10.3892/or.2016.4699 27035659

[213] Poillet-Perez L Xie X Zhan L Yang Y Sharp DW Hu ZS. Autophagy maintains tumour growth through circulating arginine. Nature (2018) 563(7732):569–73. 10.1038/s41586-018-0697-7 PMC6287937 30429607

[214] Saha S Panigrahi DP Patil S Bhutia SK. Autophagy in health and disease: A comprehensive review. BioMed Pharmacother (2018) 104(February):485–95. 10.1016/j.biopha.2018.05.007 29800913

[215] Perot BP Ingersoll MA Albert ML. The impact of macroautophagy on CD8 + T-cell-mediated antiviral immunity. Immunol Rev (2013) 255(1):40–56. 10.1111/imr.12096 23947346

[216] Abdoli A Alirezaei M Mehrbod P Forouzanfar F. Autophagy: The multi-purpose bridge in viral infections and host cells. Rev Med Virol (2018) 28(4):e1973. 10.1002/rmv.1973 29709097 PMC7169200

[217] Rey-Jurado E Riedel CA González PA Bueno SM Kalergis AM. Contribution of autophagy to antiviral immunity. FEBS Lett (2015) 589(22):3461–70. 10.1016/j.febslet.2015.07.047 PMC7094639 26297829

[218] Tang D Kang R Coyne CB Zeh HJ Lotze MT. PAMPs and DAMPs: signal 0s that spur autophagy and immunity. Immunol Rev (2012) 249(1):158–75. 10.1111/j.1600-065X.2012.01146.x PMC3662247 22889221

[219] Wang Z Li C. Xenophagy in innate immunity: A battle between host and pathogen. Dev Comp Immunol (2020) 109(March):103693. 10.1016/j.dci.2020.103693 32243873

[220] Wu M-F Chen S-T Hsieh S-L. Distinct regulation of dengue virus-induced inflammasome activation in human macrophage subsets. J BioMed Sci (2013) 20(1):36. 10.1186/1423-0127-20-36 23742038 PMC3686598

[221] Ojha CR Rodriguez M Karuppan MKM Lapierre J Kashanchi F El-Hage N. Toll-like receptor 3 regulates Zika virus infection and associated host inflammatory response in primary human astrocytes. Jin D-Y, ed. PloS One (2019) 14(2):e0208543. 10.1371/journal.pone.0208543 30735502 PMC6368285

[222] Li Y Wang L-X Yang G Hao F Urba WJ Hu H-M. Efficient Cross-presentation Depends on Autophagy in Tumor Cells. Cancer Res (2008) 68(17):6889–95. 10.1158/0008-5472.CAN-08-0161 PMC2905686 18757401

[223] Hanada T Noda NN Satomi Y Ichimura Y Fujioka Y Takao T. The Atg12-Atg5 Conjugate Has a Novel E3-like Activity for Protein Lipidation in Autophagy. J Biol Chem (2007) 282(52):37298–302. 10.1074/jbc.C700195200 17986448

[224] Shaw PJ Lamkanfi M Kanneganti T-D. NOD-like receptor (NLR) signaling beyond the inflammasome. Eur J Immunol (2010) 40(3):624–7. 10.1002/eji.200940211 PMC3001129 20201016

[225] Elinav E Strowig T Henao-Mejia J Flavell RA. Regulation of the Antimicrobial Response by NLR Proteins. Immunity (2011) 34(5):665–79. 10.1016/j.immuni.2011.05.007 21616436

[226] Caruso R Warner N Inohara N Núñez G. NOD1 and NOD2: Signaling, Host Defense, and Inflammatory Disease. Immunity (2014) 41(6):898–908. 10.1016/j.immuni.2014.12.010 25526305 PMC4272446

[227] Philpott DJ Sorbara MT Robertson SJ Croitoru K Girardin SE. NOD proteins: regulators of inflammation in health and disease. Nat Rev Immunol (2014) 14(1):9–23. 10.1038/nri3565 24336102

[228] Xi J Yan M Li S Song H Liu L Shen Z. NOD1 activates autophagy to aggravate hepatic ischemia-reperfusion injury in mice. J Cell Biochem (2019) 120(6):10605–12. 10.1002/jcb.28349 30644583

[229] Negroni A Colantoni E Vitali R Palone F Pierdomenico M Constanzo M. NOD2 induces autophagy to control AIEC bacteria infectiveness in intestinal epithelial cells. Inflamm Res (2016) 65(10):803–13. 10.1007/s00011-016-0964-8 27335178

[230] Osorio F Reis e Sousa C. Myeloid C-type Lectin Receptors in Pathogen Recognition and Host Defense. Immunity (2011) 34(5):651–64. 10.1016/j.immuni.2011.05.001 21616435

[231] Pahari S Negi S Aqdas M Arnett E Schlesinger LS Agrewala JN. Induction of autophagy through CLEC4E in combination with TLR4: an innovative strategy to restrict the survival of Mycobacterium tuberculosis. Autophagy (2020) 16(6):1021–43. 10.1080/15548627.2019.1658436 PMC7469444 31462144

[232] Rodriguez KR Bruns AM Horvath CM. MDA5 and LGP2: Accomplices and Antagonists of Antiviral Signal Transduction. J Virol (2014) 88(15):8194–200. 10.1128/JVI.00640-14 PMC4135949 24850739

[233] Subramani S Malhotra V. Non-autophagic roles of autophagy-related proteins. EMBO Rep (2013) 14(2):143–51. 10.1038/embor.2012.220 PMC3566844 23337627

[234] Ahlers LRH Goodman AG. Nucleic Acid Sensing and Innate Immunity: Signaling Pathways Controlling Viral Pathogenesis and Autoimmunity. Curr Clin Microbiol Rep (2016) 3(3):132–41. 10.1007/s40588-016-0043-5 PMC5108628 27857881

[235] Tian Y Wang M-L Zhao J. Crosstalk between Autophagy and Type I Interferon Responses in Innate Antiviral Immunity. Viruses (2019) 11(2):132. 10.3390/v11020132 PMC6409909 30717138

[236] Liang Q Seo GJ Choi YJ Kwak M-J Ge J Rodgers MA. Crosstalk between the cGAS DNA Sensor and Beclin-1 Autophagy Protein Shapes Innate Antimicrobial Immune Responses. Cell Host Microbe (2014) 15(2):228–38. 10.1016/j.chom.2014.01.009 PMC3950946 24528868

[237] Randow F Münz C. Autophagy in the regulation of pathogen replication and adaptive immunity. Trends Immunol (2012) 33(10):475–87. 10.1016/j.it.2012.06.003 PMC3461100 22796170

[238] Van Kaer L Parekh VV Postoak JL Wu L. Role of autophagy in MHC class I-restricted antigen presentation. Mol Immunol (2019) 113(October):2–5. 10.1016/j.molimm.2017.10.021 29126597 PMC5940586

[239] Mintern JD Macri C Villadangos JA. Modulation of antigen presentation by intracellular trafficking. Curr Opin Immunol (2015) 34(Mhc I):16–21. 10.1016/j.coi.2014.12.006 25578446

[240] Joffre OP Segura E Savina A Amigorena S. Cross-presentation by dendritic cells. Nat Rev Immunol (2012) 12(8):557–69. 10.1038/nri3254 22790179

[241] Noubade R Majri-Morrison S Tarbell KV. Beyond cDC1: Emerging Roles of DC Crosstalk in Cancer Immunity. Front Immunol (2019) 10:1014(MAY). 10.3389/fimmu.2019.01014 31143179 PMC6521804

[242] Merzougui N Kratzer R Saveanu L van Endert P. A proteasome-dependent, TAP-independent pathway for cross-presentation of phagocytosed antigen. EMBO Rep (2011) 12(12):1257–64. 10.1038/embor.2011.203 PMC3245693 22037009

[243] Uhl M Kepp O Jusforgues-Saklani H Vicencio J-M Kroemer G Albert ML. Autophagy within the antigen donor cell facilitates efficient antigen cross-priming of virus-specific CD8+ T cells. Cell Death Differ (2009) 16(7):991–1005. 10.1038/cdd.2009.8 19229247

[244] Münz C. Autophagy Beyond Intracellular MHC Class II Antigen Presentation. Trends Immunol (2016) 37(11):755–63. 10.1016/j.it.2016.08.017 27667710

[245] Schelhaas M. Viruses and cancer: molecular relations and perspectives. Biol Chem (2017) 398(8):815–6. 10.1515/hsz-2017-0176 28628473

[246] Kvansakul M Hinds MG. Structural biology of the Bcl-2 family and its mimicry by viral proteins. Cell Death Dis (2013) 4(11):e909–9. 10.1038/cddis.2013.436 PMC3847314 24201808

[247] Wimmer P Schreiner S. Viral mimicry to usurp ubiquitin and sumo host pathways. Viruses (2015) 7(9):4854–77. 10.3390/v7092849 PMC4584293 26343706

[248] Wong HH Sanyal S. Manipulation of autophagy by (+) RNA viruses. Semin Cell Dev Biol (2020) 101(March):3–11. 10.1016/j.semcdb.2019.07.013 31382014 PMC7102625

[249] Bird SW Maynard ND Covert MW Kirkegaard K. Nonlytic viral spread enhanced by autophagy components. Proc Natl Acad Sci (2014) 111(36):13081–6. 10.1073/pnas.1401437111 PMC4246951 25157142

[250] Jackson WT Giddings TH Taylor MP Mulinyawe S Rabinovitch M Kopito RR. Subversion of Cellular Autophagosomal Machinery by RNA Viruses. Sugden B, ed. PloS Biol (2005) 3(5):e156. 10.1371/journal.pbio.0030156 15884975 PMC1084330

[251] Alirezaei M Flynn CT Wood MR Harkins S Whitton JL. Coxsackievirus can exploit LC3 in both autophagy-dependent and -independent manners in vivo. Autophagy (2015) 11(8):1389–407. 10.1080/15548627.2015.1063769 PMC4590631 26090585

[252] Mohamud Y Shi J Qu J Poon T Xue YC Deng H. Enteroviral Infection Inhibits Autophagic Flux via Disruption of the SNARE Complex to Enhance Viral Replication. Cell Rep (2018) 22(12):3292–303. 10.1016/j.celrep.2018.02.090 29562184

[253] Yoon SY Ha YE Choi JE Ahn J Leen H Kweon H-S. Coxsackievirus B4 Uses Autophagy for Replication after Calpain Activation in Rat Primary Neurons. J Virol (2008) 82(23):11976–8. 10.1128/JVI.01028-08 PMC2583648 18799585

[254] Lee Y-R Wang P-S Wang J-R Liu H-S. Enterovirus 71-induced autophagy increases viral replication and pathogenesis in a suckling mouse model. J BioMed Sci (2014) 21(1):80. 10.1186/s12929-014-0080-4 25139436 PMC4237791

[255] Klein KA Jackson WT. Human Rhinovirus 2 Induces the Autophagic Pathway and Replicates More Efficiently in Autophagic Cells. J Virol (2011) 85(18):9651–4. 10.1128/JVI.00316-11 PMC3165776 21752910

[256] O’Donnell V Pacheco JM LaRocco M Burrage T Jackson W Rodríguez LL. Foot-and-mouth disease virus utilizes an autophagic pathway during viral replication. Virology (2011) 410(1):142–50. 10.1016/j.virol.2010.10.042 PMC7126820 21112602

[257] Hou L Ge X Xin L Zhou L Guo X Yang H. Nonstructural proteins 2C and 3D are involved in autophagy as induced by the encephalomyocarditis virus. Virol J (2014) 11(1):156. 10.1186/1743-422X-11-156 25178311 PMC4161894

[258] Heaton NS Randall G. Dengue Virus-Induced Autophagy Regulates Lipid Metabolism. Cell Host Microbe (2010) 8(5):422–32. 10.1016/j.chom.2010.10.006 PMC3026642 21075353

[259] Zhang J Lan Y Li MY Lamers MM Fusade-Boyer M Klemm E. Flaviviruses Exploit the Lipid Droplet Protein AUP1 to Trigger Lipophagy and Drive Virus Production. Cell Host Microbe (2018) 23(6):819–31.e5. 10.1016/j.chom.2018.05.005 29902443

[260] Offerdahl DK Dorward DW Hansen BT Bloom ME. Cytoarchitecture of Zika virus infection in human neuroblastoma and Aedes albopictus cell lines. Virology (2017) 501(November 2016):54–62. 10.1016/j.virol.2016.11.002 27863275 PMC5201448

[261] Cortese M Goellner S Acosta EG Neufeldt CJ Oleksiuk O Lampe M. Ultrastructural Characterization of Zika Virus Replication Factories. Cell Rep (2017) 18(9):2113–23. 10.1016/j.celrep.2017.02.014 PMC5340982 28249158

[262] de Haan CAM Molinari M Reggiori F. Autophagy-independent LC3 function in vesicular traffic. Autophagy (2010) 6(7):994–6. 10.4161/auto.6.7.13309 20814233

[263] Sun Y Yu S Ding N Meng C Meng S Zhang S. Autophagy Benefits the Replication of Newcastle Disease Virus in Chicken Cells and Tissues. J Virol (2014) 88(1):525–37. 10.1128/JVI.01849-13 PMC3911706 24173218

[264] Prentice E Jerome WG Yoshimori T Mizushima N Denison MR. Coronavirus Replication Complex Formation Utilizes Components of Cellular Autophagy. J Biol Chem (2004) 279(11):10136–41. 10.1074/jbc.M306124200 PMC7957857 14699140

[265] Krejbich-Trotot P Gay B Li-Pat-Yuen G Hoarau J-J Jaffar-Bandjee M-C Briant L. Chikungunya triggers an autophagic process which promotes viral replication. Virol J (2011) 8(1):432. 10.1186/1743-422X-8-432 21902836 PMC3179960

[266] Sharma M Bhattacharyya S Sharma KB Chauhan S Asthana S Abdin MZ. Japanese encephalitis virus activates autophagy through XBP1 and ATF6 ER stress sensors in neuronal cells. J Gen Virol (2017) 98(5):1027–39. 10.1099/jgv.0.000792 28535855

[267] Dales S Eggers HJ Tamm I Palade GE. Electron microscopic study of the formation of poliovirus. Virology (1965) 26(3):379–89. 10.1016/0042-6822(65)90001-2 14319710

[268] Jackson WT. Poliovirus-induced changes in cellular membranes throughout infection. Curr Opin Virol (2014) 9:67–73. 10.1016/j.coviro.2014.09.007 25310497 PMC4267968

[269] Corona Velazquez A Corona AK Klein KA Jackson WT. Poliovirus induces autophagic signaling independent of the ULK1 complex. Autophagy (2018) 14(7):1201–13. 10.1080/15548627.2018.1458805 PMC6103675 29929428

[270] Wang H Li K Ma L Wu S Hu J Yan H. Berberine inhibits enterovirus 71 replication by downregulating the MEK/ERK signaling pathway and autophagy. Virol J (2017) 14(1):2. 10.1186/s12985-016-0674-4 28081706 PMC5234143

[271] Rozières A Viret C Faure M. Autophagy in Measles Virus Infection. Viruses (2017) 9(12):359. 10.3390/v9120359 PMC5744134 29186766

[272] Chiramel A Brady N Bartenschlager R. Divergent Roles of Autophagy in Virus Infection. Cells (2013) 2(1):83–104. 10.3390/cells2010083 24709646 PMC3972664

[273] Joubert P-E Meiffren G Grégoire IP Guillemette P Clémence R Flacher M. Autophagy Induction by the Pathogen Receptor CD46. Cell Host Microbe (2009) 6(4):354–66. 10.1016/j.chom.2009.09.006 19837375

[274] Richetta C Grégoire IP Verlhac P Azocar O Baguet J Flacher M. Sustained Autophagy Contributes to Measles Virus Infectivity. Deretic V, ed. PloS Pathog (2013) 9(9):e1003599. 10.1371/journal.ppat.1003599 24086130 PMC3784470

[275] Cordero R. Patogénesis del VIH / SIDA. Rev Clínica la Esc Med (2017) 7(5):28–46.

[276] Moir S Fauci AS. B-cell responses to HIV infection. Immunol Rev (2017) 275(1):33–48. 10.1111/imr.12502 28133792 PMC5300048

[277] Yarchoan R Uldrick TS. HIV-Associated Cancers and Related Diseases. Longo DL, ed. N Engl J Med (2018) 378(11):1029–41. 10.1056/NEJMra1615896 PMC6890231 29539283

[278] Wood NH Feller L. The malignant potential of HIV-associated Kaposi sarcoma. Cancer Cell Int (2008) 8(1):14. 10.1186/1475-2867-8-14 18976452 PMC2633277

[279] Lu D-Y Wu H-Y Yarla NS Xu B Ding J Lu T-R. HAART in HIV/AIDS Treatments: Future Trends. Infect Disord Drug Targets (2018) 18(1):15–22. 10.2174/1871526517666170505122800 28474549

[280] Tang S-W Ducroux A Jeang K-T Neuveut C. Impact of cellular autophagy on viruses: Insights from hepatitis B virus and human retroviruses. J BioMed Sci (2012) 19(1):92. 10.1186/1423-0127-19-92 23110561 PMC3495035

[281] Castro-Gonzalez S Shi Y Colomer-Lluch M Song Y Mowery K Almodovar S. HIV-1 Nef counteracts autophagy restriction by enhancing the association between BECN1 and its inhibitor BCL2 in a PRKN-dependent manner. Autophagy (2020) 00(00):1–25. 10.1080/15548627.2020.1725401 PMC8007141 32097085

[282] Jia P. Autophagy and virus infection. Infect Int (2018) 6(4):124–8. 10.2478/ii-2018-0001

[283] Wang X Gao Y Tan J Devadas K Ragupathy V Takeda K. HIV-1 and HIV-2 infections induce autophagy in Jurkat and CD4+ T cells. Cell Signal (2012) 24(7):1414–9. 10.1016/j.cellsig.2012.02.016 22406083

[284] Neher RA Dyrdak R Druelle V Hodcroft EB Albert J. Potential impact of seasonal forcing on a SARS-CoV-2 pandemic. Swiss Med Wkly (2020) 150(March):w20224. 10.4414/smw.2020.20224 32176808

[285] Raoult D Zumla A Locatelli F Ippolito G Kroemer G. Coronavirus infections: Epidemiological, clinical and immunological features and hypotheses. Cell Stress (2020) 4(4):66–75. 10.15698/cst2020.04.216 32292881 PMC7064018

[286] Carmona-Gutierrez D Bauer MA Zimmermann A Kainz K Hofer SJ Kroemer G. Digesting the crisis: autophagy and coronaviruses. Microb Cell (2020) 2020:7(5):119–128. 10.15698/mic2020.05.715 PMC7199282 32391393

[287] Petrova VN Russell CA. The evolution of seasonal influenza viruses. Nat Rev Microbiol (2018) 16(1):47–60. 10.1038/nrmicro.2017.118 29081496

[288] Paulino J Pang X Hung I Zhou H-X Cross TA. Influenza A M2 Channel Clustering at High Protein/Lipid Ratios: Viral Budding Implications. Biophys J (2019) 116(6):1075–84. 10.1016/j.bpj.2019.01.042 PMC6428927 30819568

[289] Beale R Wise H Stuart A Ravenhill BJ Digard P Randow F. A LC3-Interacting Motif in the Influenza A Virus M2 Protein Is Required to Subvert Autophagy and Maintain Virion Stability. Cell Host Microbe (2014) 15(2):239–47. 10.1016/j.chom.2014.01.006 PMC3991421 24528869

[290] Joubert P-E Werneke SW de la Calle C Florence G-B Alessandra G Lucie P. Chikungunya virus–induced autophagy delays caspase-dependent cell death. J Exp Med (2012) 209(5):1029–47. 10.1084/jem.20110996 PMC3348111 22508836

[291] Jordan TX Randall G. Manipulation or capitulation: virus interactions with autophagy. Microbes Infect (2012) 14(2):126–39. 10.1016/j.micinf.2011.09.007 PMC3264745 22051604

[292] Zompi S Harris E. Animal Models of Dengue Virus Infection. Viruses (2012) 4(1):62–82. 10.3390/v4010062 22355452 PMC3280519

[293] Heaton NS Randall G. Dengue Virus and Autophagy. Viruses (2011) 3(8):1332–41. 10.3390/v3081332 PMC3185800 21994782

[294] Musso D Ko AI Baud D. Zika Virus Infection — After the Pandemic. Longo DL, ed. N Engl J Med (2019) 381(15):1444–57. 10.1056/NEJMra1808246 31597021

[295] Chiramel AI Best SM. Role of autophagy in Zika virus infection and pathogenesis. Virus Res (2018) 254:34–40. 10.1016/j.virusres.2017.09.006 28899653 PMC5844781

[296] Fishbein A Hammock BD Serhan CN Panigrahy D. Carcinogenesis: Failure of resolution of inflammation? Pharmacol Ther (2020) 61(18):107670. 10.1016/j.pharmthera.2020.107670 PMC7470770 32891711

[297] Kakavandi E Shahbahrami R Goudarzi H Eslami G Faghihloo E. Anoikis resistance and oncoviruses. J Cell Biochem (2018) 119(3):2484–91. 10.1002/jcb.26363 28836703

[298] Tornesello ML Annunziata C Tornesello AL Buonaguro L Buonaguro FM. Human Oncoviruses and p53 Tumor Suppressor Pathway Deregulation at the Origin of Human Cancers. Cancers (Basel) (2018) 10(7):213. 10.3390/cancers10070213 PMC6071257 29932446

[299] Mesri EA Feitelson MA Munger K. Human Viral Oncogenesis: A Cancer Hallmarks Analysis. Cell Host Microbe (2014) 15(3):266–82. 10.1016/j.chom.2014.02.011 PMC3992243 24629334

[300] Mui U Haley C Tyring S. Viral Oncology: Molecular Biology and Pathogenesis. J Clin Med (2017) 6(12):111. 10.3390/jcm6120111 PMC5742800 29186062

[301] Krump NA You J. Molecular mechanisms of viral oncogenesis in humans. Nat Rev Microbiol (2018) 16(11):684–98. 10.1038/s41579-018-0064-6 PMC6336458 30143749

[302] Tornesello ML Buonaguro L Buonaguro FM. An overview of new biomolecular pathways in pathogen-related cancers. Future Oncol (2015) 11(11):1625–39. 10.2217/fon.15.87 26043216

[303] Akram N Imran M Noreen M Ahmed F Atif M Fatima Z. Oncogenic Role of Tumor Viruses in Humans. Viral Immunol (2017) 30(1):20–7. 10.1089/vim.2016.0109 27830995

[304] Jackson WT. Viruses and the autophagy pathway. Virology (2015) 479-480:450–6. 10.1016/j.virol.2015.03.042 PMC5917100 25858140

[305] Levine B Mizushima N Virgin HW. Autophagy in immunity and inflammation. Nature (2011) 469(7330):323–35. 10.1038/nature09782 PMC3131688 21248839

[306] Haley CT Mui UN Vangipuram R Rady PL Tyring SK. Human oncoviruses: Mucocutaneous manifestations, pathogenesis, therapeutics, and prevention. J Am Acad Dermatol (2019) 81(1):1–21. 10.1016/j.jaad.2018.09.062 30502418

[307] Ringehan M McKeating JA Protzer U. Viral hepatitis and liver cancer. Philos Trans R Soc B Biol Sci (2017) 372(1732):20160274. 10.1098/rstb.2016.0274 PMC5597741 28893941

[308] Cao J Li D. Searching for human oncoviruses: Histories, challenges, and opportunities. J Cell Biochem (2018) 119(6):4897–906. 10.1002/jcb.26717 29377246

[309] Herbein G. The Human Cytomegalovirus, from Oncomodulation to Oncogenesis. Viruses (2018) 10(8):408. 10.3390/v10080408 PMC6115842 30081496

[310] Yamagishi M Fujikawa D Watanabe T Uchimaru K. HTLV-1-Mediated Epigenetic Pathway to Adult T-Cell Leukemia–Lymphoma. Front Microbiol (2018) 9:1686(JUL). 10.3389/fmicb.2018.01686 30087673 PMC6066519

[311] Vescovo T Pagni B Piacentini M Fimia GM Antonioli M. Regulation of Autophagy in Cells Infected With Oncogenic Human Viruses and Its Impact on Cancer Development. Front Cell Dev Biol (2020) 8:47(February). 10.3389/fcell.2020.00047 32181249 PMC7059124

[312] McElwee M Vijayakrishnan S Rixon F Bhella D. Structure of the herpes simplex virus portal-vertex. Sugden B, ed. PloS Biol (2018) 16(6):e2006191. 10.1371/journal.pbio.2006191 29924793 PMC6028144

[313] Agelidis AM Shukla D. Cell entry mechanisms of HSV: what we have learned in recent years. Future Virol (2015) 10(10):1145–54. 10.2217/fvl.15.85 PMC4822157 27066105

[314] Jha H Banerjee S Robertson E. The Role of Gammaherpesviruses in Cancer Pathogenesis. Pathogens (2016) 5(1):18. 10.3390/pathogens5010018 PMC4810139 26861404

[315] Chang Y Moore PS Weiss RA. Human oncogenic viruses: nature and discovery. Philos Trans R Soc B Biol Sci (2017) 372(1732):20160264. 10.1098/rstb.2016.0264 PMC5597731 28893931

[316] Cirone M. EBV and KSHV Infection Dysregulates Autophagy to Optimize Viral Replication, Prevent Immune Recognition and Promote Tumorigenesis. Viruses (2018) 10(11):599. 10.3390/v10110599 PMC6266050 30384495

[317] Chang Y Cesarman E Pessin M Lee F Culpepper J Knowles D. Identification of herpesvirus-like DNA sequences in AIDS-associated Kaposi’s sarcoma. Science (80- ) (1994) 266(5192):1865–9. 10.1126/science.7997879 7997879

[318] Calabrò ML Sarid R. Human Herpesvirus 8 And Lymphoproliferative Diseases. Mediterr J Hematol Infect Dis (2018) 10(1):e2018061. 10.4084/mjhid.2018.061 30416693 PMC6223575

[319] De Paoli P Carbone A. Kaposi’s Sarcoma Herpesvirus: twenty years after its discovery. Eur Rev Med Pharmacol Sci (2016) 20(7):1288–94. 27097948

[320] Wakeman BS Izumiya Y Speck SH. Identification of Novel Kaposi’s Sarcoma-Associated Herpesvirus Orf50 Transcripts: Discovery of New RTA Isoforms with Variable Transactivation Potential. Jung JU, ed. J Virol (2017) 91(1):1–23. 10.1128/JVI.01434-16 PMC5165194 27795414

[321] Cesarman E Damania B Krown SE Martin J Bower M Whitby D. Kaposi sarcoma. Nat Rev Dis Primers (2019) 5(1):9. 10.1038/s41572-019-0060-9 30705286 PMC6685213

[322] Veettil M Bandyopadhyay C Dutta D Chandran B. Interaction of KSHV with Host Cell Surface Receptors and Cell Entry. Viruses (2014) 6(10):4024–46. 10.3390/v6104024 PMC4213576 25341665

[323] Giffin L Damania B. Chapter Two-KSHV: Pathways to Tumorigenesis and Persisten Infection. Advances in Virus Research, 1st ed, vol. 88. . Elsevier Inc (2014). p. 111–59. 10.1016/B978-0-12-800098-4.00002-7 PMC4104069 24373311

[324] Rosario SA Santiago GE Mesri EA Verdun RE. Kaposi’s Sarcoma-Associated Herpesvirus-Encoded Viral IL-6 (vIL-6) Enhances Immunoglobulin Class-Switch Recombination. Front Microbiol (2018) 9:3119(December). 10.3389/fmicb.2018.03119 30619193 PMC6305588

[325] Choi Y Bowman JW Jung JU. Autophagy during viral infection — a double-edged sword. Nat Rev Microbiol (2018) 16(6):341–54. 10.1038/s41579-018-0003-6 PMC6907743 29556036

[326] Ueda K. KSHV Genome Replication and Maintenance in Latency. Adv Exp Med Biol (2018) 1045:299–320. 10.1007/978-981-10-7230-7_14 29896673

[327] Wang L. The Kaposi’s Sarcoma-Associated Herpesvirus (KSHV/HHV-8) K1 Protein Induces Expression of Angiogenic and Invasion Factors. Cancer Res (2004) 64(8):2774–81. 10.1158/0008-5472.CAN-03-3653 15087393

[328] Leidal AM Cyr DP Hill RJ Lee PWK McCormick C. Subversion of Autophagy by Kaposi’s Sarcoma-Associated Herpesvirus Impairs Oncogene-Induced Senescence. Cell Host Microbe (2012) 11(2):167–80. 10.1016/j.chom.2012.01.005 22341465

[329] Anders PM Zhang Z Bhende PM Giffin L Damania B. The KSHV K1 Protein Modulates AMPK Function to Enhance Cell Survival. Schulz TF, ed. PloS Pathog (2016) 12(11):e1005985. 10.1371/journal.ppat.1005985 27829024 PMC5102384

[330] Yin H Shao S Jiang X Xie P Sun W Yu T. Interactions between Autophagy and DNA Viruses. Viruses (2019) 11(9):776. 10.3390/v11090776 PMC6784137 31450758

[331] He M Zhang W Bakken T Schutten M Toth Z Jung JU. Cancer Angiogenesis Induced by Kaposi Sarcoma-Associated Herpesvirus Is Mediated by EZH2. Cancer Res (2012) 72(14):3582–92. 10.1158/0008-5472.CAN-11-2876 PMC4034471 22593192

[332] Granato M Santarelli R Filardi M Gonnrlls R Farina A Torrisi MR. The activation of KSHV lytic cycle blocks autophagy in PEL cells. Autophagy (2015) 11(11):1978–86. 10.1080/15548627.2015.1091911 PMC4824593 26391343

[333] Santarelli R Gonnella R Di Giovenale G Cuomo L Capobianchi A Granato M. STAT3 activation by KSHV correlates with IL-10, IL-6 and IL-23 release and an autophagic block in dendritic cells. Sci Rep (2015) 4(1):4241. 10.1038/srep04241 PMC3937791 24577500

[334] Yan L Majerciak V Zheng Z-M Lan K. Towards Better Understanding of KSHV Life Cycle: from Transcription and Posttranscriptional Regulations to Pathogenesis. Virol Sin (2019) 34(2):135–61. 10.1007/s12250-019-00114-3 PMC6513836 31025296

[335] Broussard G Damania B. Regulation of KSHV Latency and Lytic Reactivation. Viruses (2020) 12(9):1034. 10.3390/v12091034 PMC7551196 32957532

[336] De Leo A Chen H-S Hu C-CA Lieberman PM. Deregulation of KSHV latency conformation by ER-stress and caspase-dependent RAD21-cleavage. Flemington EK, ed. PloS Pathog (2017) 13(8):e1006596. 10.1371/journal.ppat.1006596 28854249 PMC5595345

[337] Guito J Lukac D. KSHV Reactivation and Novel Implications of Protein Isomerization on Lytic Switch Control. Viruses (2015) 7(1):72–109. 10.3390/v7010072 25588053 PMC4306829

[338] Pringle ES Robinson C-A McCormick C. Kaposi’s Sarcoma-Associated Herpesvirus Lytic Replication Interferes with mTORC1 Regulation of Autophagy and Viral Protein Synthesis. Jung JU, ed. J Virol (2019) 93(21):1–22. 10.1128/JVI.00854-19 PMC6803247 31375594

[339] Wen H-J Yang Z Zhou Y Wood C. Enhancement of Autophagy during Lytic Replication by the Kaposi’s Sarcoma-Associated Herpesvirus Replication and Transcription Activator. J Virol (2010) 84(15):7448–58. 10.1128/JVI.00024-10 PMC2897602 20484505

[340] Mariggiò G Koch S Zhang G Weidner-Glunde M Rϋckert J Kati S. Kaposi Sarcoma Herpesvirus (KSHV) Latency-Associated Nuclear Antigen (LANA) recruits components of the MRN (Mre11-Rad50-NBS1) repair complex to modulate an innate immune signaling pathway and viral latency. Robertson ES, ed. PloS Pathog (2017) 13(4):e1006335. 10.1371/journal.ppat.1006335 28430817 PMC5415203

[341] Gilardini Montani MS Falcinelli L Santarelli R Romeo MA Granato M Faggioni A. Kaposi Sarcoma Herpes Virus (KSHV) infection inhibits macrophage formation and survival by counteracting Macrophage Colony-Stimulating Factor (M-CSF)-induced increase of Reactive Oxygen Species (ROS), c-Jun N-terminal kinase (JNK) phosphorylation and auto. Int J Biochem Cell Biol (2019) 114(June):105560. 10.1016/j.biocel.2019.06.008 31220583

[342] Cai Q Chen K Young KH. Epstein–Barr virus-positive T/NK-cell lymphoproliferative disorders. Exp Mol Med (2015) 47(1):e133–3. 10.1038/emm.2014.105 PMC4314580 25613730

[343] Shinozaku-Ushiku A Kunita A Fukuyama M. Update on Epstein-Barr virus and gastric cancer (Review). Int J Oncol (2015) 46(4):1421–34. 10.3892/ijo.2015.2856 25633561

[344] Stanfield BA Luftig MA. Recent advances in understanding Epstein-Barr virus. F1000Research (2017) 6(0):386. 10.12688/f1000research.10591.1 28408983 PMC5373418

[345] Pujals A Favre L Pioche-Durieu C Robert A Meurice G Le Gentil M. Constitutive autophagy contributes to resistance to TP53-mediated apoptosis in Epstein-Barr virus-positive latency III B-cell lymphoproliferations. Autophagy (2015) 11(12):2275–87. 10.1080/15548627.2015.1115939 PMC4835200 26565591

[346] Farrell PJ. Epstein–Barr Virus and Cancer. Annu Rev Pathol Mech Dis (2019) 14(1):29–53. 10.1146/annurev-pathmechdis-012418-013023 30125149

[347] Ascherio A Munger KL. Epstein–Barr Virus Infection and Multiple Sclerosis: A Review. J Neuroimmune Pharmacol (2010) 5(3):271–7. 10.1007/s11481-010-9201-3 20369303

[348] Chen J. Roles of the PI3K/Akt pathway in Epstein-Barr virus-induced cancers and therapeutic implications. World J Virol (2012) 1(6):154. 10.5501/wjv.v1.i6.154 24175221 PMC3782276

[349] Fang W-L Huang K-H Lan Y-T Lin C-H Chang S-C Chen M-H. Mutations in PI3K/AKT pathway genes and amplifications of PI3KCA are associated with patterns of recurrence in gastric cancers. Oncotarget (2016) 7(5):6201–20. 10.18632/oncotarget.6641 PMC4868750 26701847

[350] Cen O Longnecker R. Latent Membrane Protein 2 (LMP2). Epstein Barr Virus Volume 2. Curr Top Microbiol Immunol (2015) 391:151–80. 10.1007/978-3-319-22834-1_5 26428374

[351] Fish K Sora RP Schaller SJ Longnecker R Ikeda M. EBV latent membrane protein 2A orchestrates p27kip1 degradation via Cks1 to accelerate MYC-driven lymphoma in mice. Blood (2017) 130(23):2516–26. 10.1182/blood-2017-07-796821 PMC5721284 29074502

[352] Smatti MK Al-Sadeq DW Ali NH Pintus G Abou-Saleh H Nasrallah GK. Epstein–Barr Virus Epidemiology, Serology, and Genetic Variability of LMP-1 Oncogene Among Healthy Population: An Update. Front Oncol (2018) 8:211(JUN):1–16. 10.3389/fonc.2018.00211 29951372 PMC6008310

[353] Hurwitz SN Cheerathodi MR Nkosi D York SB Meckes DG. Tetraspanin CD63 Bridges Autophagic and Endosomal Processes To Regulate Exosomal Secretion and Intracellular Signaling of Epstein-Barr Virus LMP1. Longnecker RM, ed. J Virol (2017) 92(5):1–21. 10.1128/JVI.01969-17 PMC5809724 29212935

[354] Bhattacharjee S Bose P Patel K Roy SG Gain C Gowda H. Transcriptional and epigenetic modulation of autophagy promotes EBV oncoprotein EBNA3C induced B-cell survival. Cell Death Dis (2018) 9(6):605. 10.1038/s41419-018-0668-9 29789559 PMC5964191

[355] De Leo A Colavita F Ciccosanti F Fimia GM Lieberman PM Mattia E. Inhibition of autophagy in EBV-positive Burkitt’s lymphoma cells enhances EBV lytic genes expression and replication. Cell Death Dis (2015) 6(9):e1876–6. 10.1038/cddis.2015.156 PMC4650432 26335716

[356] Nowag H Guhl B Thriene K Romao S Ziegler U Dengjel J. Macroautophagy Proteins Assist Epstein Barr Virus Production and Get Incorporated Into the Virus Particles. EBioMedicine (2014) 1(2-3):116–25. 10.1016/j.ebiom.2014.11.007 PMC4457436 26137519

[357] Granato M Santarelli R Farina A Gonnela R Lotti LV Faggioni A. Epstein-Barr Virus Blocks the Autophagic Flux and Appropriates the Autophagic Machinery To Enhance Viral Replication. J Virol (2014) 88(21):12715–26. 10.1128/JVI.02199-14 PMC4248894 25142602

[358] Hung C-H Chen L-W Wang W-H Chang P-J Chiu Y-F Hung C-C. Regulation of Autophagic Activation by Rta of Epstein-Barr Virus via the Extracellular Signal-Regulated Kinase Pathway. J Virol (2014) 88(20):12133–45. 10.1128/JVI.02033-14 PMC4178756 25122800

[359] Khanim F Mackett M Young LS Dawson J Meseda CA Dawson C. BHRF1, a viral homologue of the Bcl-2 oncogene, is conserved at both the sequence and functional level in different Epstein-Barr virus isolates. J Gen Virol (1997) 78(11):2987–99. 10.1099/0022-1317-78-11-2987 9367386

[360] Marshall WL Yim C Gustafson E Graf T Sage DR Hanify K. Epstein-Barr Virus Encodes a Novel Homolog of the bcl-2 Oncogene That Inhibits Apoptosis and Associates with Bax and Bak. J Virol (1999) 73(6):5181–5. 10.1128/JVI.73.6.5181-5185.1999 PMC112567 10233985

[361] Altmann M Hammerschmidt W. Epstein-Barr Virus Provides a New Paradigm: A Requirement for the Immediate Inhibition of Apoptosis. Sugden B, ed. PloS Biol (2005) 3(12):e404. 10.1371/journal.pbio.0030404 16277553 PMC1283332

[362] Henderson S Huen D Rowe M Dawson C Johnson G Rickinson A. Epstein-Barr virus-coded BHRF1 protein, a viral homologue of Bcl-2, protects human B cells from programmed cell death. Proc Natl Acad Sci U S A (1993) 90(18):8479–83. 10.1073/pnas.90.18.8479 PMC47380 8397406

[363] Bellows DS Howell M Pearson C Hazlewood SA Hardwick JM. Epstein-Barr Virus BALF1 Is a BCL-2-Like Antagonist of the Herpesvirus Antiapoptotic BCL-2 Proteins. J Virol (2002) 76(5):2469–79. 10.1128/jvi.76.5.2469-2479.2002 PMC153809 11836425

[364] Shao Z Borde C Quignon F Escargueil A Maréchal V. Epstein–Barr Virus BALF0 and BALF1 Modulate Autophagy. Viruses (2019) 11(12):1099. 10.3390/v11121099 PMC6950364 31783609

[365] Plotkin SA Boppana SB. Vaccination against the human cytomegalovirus. Vaccine (2019) 37(50):7437–42. 10.1016/j.vaccine.2018.02.089 PMC6892274 29622379

[366] Lussignol M Esclatine A. Cytomegalovirus and Autophagy. Immunology (2018) 1:9–21. 10.1016/B978-0-12-809819-6.00002-2

[367] Joseph GP McDermott R Baryshnikova MA Cobbs CS Ulasov IV. Cytomegalovirus as an oncomodulatory agent in the progression of glioma. Cancer Lett (2017) 384:79–85. 10.1016/j.canlet.2016.10.022 27777041

[368] Chaumorcel M Lussignol M Mouna L Cavignac Y Fahie K Cotte-Laffitte J. The Human Cytomegalovirus Protein TRS1 Inhibits Autophagy via Its Interaction with Beclin 1. J Virol (2012) 86(5):2571–84. 10.1128/JVI.05746-11 PMC3302257 22205736

[369] Mouna L Hernandez E Bonte D Brost R Amazit L Delgui LR. Analysis of the role of autophagy inhibition by two complementary human cytomegalovirus BECN1/Beclin 1-binding proteins. Autophagy (2016) 12(2):327–42. 10.1080/15548627.2015.1125071 PMC4836022 26654401

[370] Mlera L Moy M Maness K Tran LN Goodrum FD. The Role of the Human Cytomegalovirus UL133-UL138 Gene Locus in Latency and Reactivation. Viruses (2020) 12(7):714. 10.3390/v12070714 PMC7411667 32630219

[371] Tey SK Khanna R. Autophagy mediates transporter associated with antigen processing- independent presentation of viral epitopes through MHC class I pathway. Blood (2012) 120(5):994–1004. 10.1182/blood-2012-01-402404 22723550

[372] Loi M Müller A Steinbach K Niven J Barreira da Silva R Paul P. Macroautophagy Proteins Control MHC Class I Levels on Dendritic Cells and Shape Anti-viral CD8 + T Cell Responses. Cell Rep (2016) 15(5):1076–87. 10.1016/j.celrep.2016.04.002 27117419

[373] Reddehase MJ Lemmermann NAW. Cellular reservoirs of latent cytomegaloviruses. Med Microbiol Immunol (2019) 208(3-4):391–403. 10.1007/s00430-019-00592-y 31011793

[374] Møller R Schwarz TM Noriega VM Panis M Sachs D Tortorella D. miRNA-mediated targeting of human cytomegalovirus reveals biological host and viral targets of IE2. Proc Natl Acad Sci (2018) 115(5):1069–74. 10.1073/pnas.1719036115 PMC5798380 29339472

[375] Li M Ball CB Collins G Hu Q Luse DS Price DH. Human cytomegalovirus IE2 drives transcription initiation from a select subset of late infection viral promoters by host RNA polymerase II. Glaunsinger BA, ed. PloS Pathog (2020) 16(4):e1008402. 10.1371/journal.ppat.1008402 32251483 PMC7162547

[376] Zhang X Xi T Zhang L Bi Y Huang Y Lu Y. The role of autophagy in human cytomegalovirus IE2 expression. J Med Virol 2020) 25:jmv.26357. 10.1002/jmv.26357 32710640

[377] Martin J Maldonado J Mueller J Zhang W Mansky L. Molecular Studies of HTLV-1 Replication: An Update. Viruses (2016) 8(2):31. 10.3390/v8020031 PMC4776186 26828513

[378] Poiesz BJ Ruscetti FW Gazdar AF Bunn PA Minna JD Gallo RC. Detection and isolation of type C retrovirus particles from fresh and cultured lymphocytes of a patient with cutaneous T-cell lymphoma. Proc Natl Acad Sci (1980) 77(12):7415–9. 10.1073/pnas.77.12.7415 PMC350514 6261256

[379] Futsch N Prates G Mahieux R Casseb J Dutartre H. Cytokine Networks Dysregulation during HTLV-1 Infection and Associated Diseases. Viruses (2018) 10(12):691. 10.3390/v10120691 PMC6315340 30563084

[380] Schierhout G McGregor S Gessain A Einsiedel L Martinello M Kaldor J. Association between HTLV-1 infection and adverse health outcomes: a systematic review and meta-analysis of epidemiological studies. Lancet Infect Dis (2020) 20(1):133–43. 10.1016/S1473-3099(19)30402-5 31648940

[381] Gessain A Cassar O. Epidemiological Aspects and World Distribution of HTLV-1 Infection. Front Microbiol (2012) 3:388(NOV). 10.3389/fmicb.2012.00388 23162541 PMC3498738

[382] Tang S-W Chen C-Y Klase Z Zane L Jeang K-T. The Cellular Autophagy Pathway Modulates Human T-Cell Leukemia Virus Type 1 Replication. J Virol (2013) 87(3):1699–707. 10.1128/JVI.02147-12

[383] Chen L Liu D Zhang Y Zhang H Cheng H. The autophagy molecule Beclin 1 maintains persistent activity of NF-κB and Stat3 in HTLV-1-transformed T lymphocytes. Biochem Biophys Res Commun (2015) 465(4):739–45. 10.1016/j.bbrc.2015.08.070 PMC4580621 26319552

[384] Nakahata S Syahrul C Nakatake A Sakamoto K Yoshihama M Nishikata I. Clinical significance of soluble CADM1 as a novel marker for adult T-cell leukemia/lymphoma. Haematologica (2020) 106(2):532–42. 10.3324/haematol.2019.234096 PMC7849584 32054656

[385] Sarkar B Nishikata I Nakahata S Ichikawa T Shiraga T Saha HR. Degradation of p47 by autophagy contributes to CADM1 overexpression in ATLL cells through the activation of NF-κB. Sci Rep (2019) 9(1):3491. 10.1038/s41598-019-39424-7 30837480 PMC6400899

[386] Mukai R Ohshima T. HTLV-1 HBZ positively regulates the mTOR signaling pathway via inhibition of GADD34 activity in the cytoplasm. Oncogene (2014) 33(18):2317–28. 10.1038/onc.2013.181 23708656

[387] Baratella M Forlani G Accolla RS. HTLV-1 HBZ Viral Protein: A Key Player in HTLV-1 Mediated Diseases. Front Microbiol (2017) 8:2615(DEC). 10.3389/fmicb.2017.02615 29312275 PMC5744428

[388] Ren T Takahashi Y Liu X Loughran TP Sun S-C Wang H-G. HTLV-1 Tax deregulates autophagy by recruiting autophagic molecules into lipid raft microdomains. Oncogene (2015) 34(3):334–45. 10.1038/onc.2013.552 PMC4067462 24362528

[389] Manns MP Buti M Gane E Pawlotsky J-M Razavi H Terrault N. Hepatitis C virus infection. Nat Rev Dis Primers (2017) 3(1):17006. 10.1038/nrdp.2017.6 28252637

[390] Balakrishnan M Glover MT Kanwal F. Hepatitis C and Risk of Nonhepatic Malignancies. Clin Liver Dis (2017) 21(3):543–54. 10.1016/j.cld.2017.03.009 28689592

[391] Pol S Vallet-Pichard A Hermine O. Extrahepatic cancers and chronic HCV infection. Nat Rev Gastroenterol Hepatol (2018) 15(5):283–90. 10.1038/nrgastro.2017.172 29339810

[392] Paul D Hoppe S Saher G Krijnse-Locker J Bartenschlager R. Morphological and Biochemical Characterization of the Membranous Hepatitis C Virus Replication Compartment. J Virol (2013) 87(19):10612–27. 10.1128/JVI.01370-13 PMC3807400 23885072

[393] Ferraris P Beaumont E Uzbekov R Brand D Gaillard J Blanchard E. Sequential biogenesis of host cell membrane rearrangements induced by hepatitis C virus infection. Cell Mol Life Sci (2013) 70(7):1297–306. 10.1007/s00018-012-1213-0 PMC4901162 23184194

[394] Vescovo T Refolo G Romagnoli A Ciccosanti F Corazzari M Alonzi T. Autophagy in HCV Infection: Keeping Fat and Inflammation at Bay. BioMed Res Int (2014) 2014:1–10. 10.1155/2014/265353 PMC4138948 25162004

[395] Fahmy AM Labonté P. The autophagy elongation complex (ATG5-12/16L1) positively regulates HCV replication and is required for wild-type membranous web formation. Sci Rep (2017) 7(1):40351. 10.1038/srep40351 28067309 PMC5220323

[396] Wang L James Ou J. Hepatitis C virus and autophagy. Biol Chem (2015) 396(11):1215–22. 10.1515/hsz-2015-0172 PMC4778564 26024249

[397] Jassey A Liu C-H Changou C Richardson C Hsu H-Y Lin L-T. Hepatitis C Virus Non-Structural Protein 5A (NS5A) Disrupts Mitochondrial Dynamics and Induces Mitophagy. Cells (2019) 8(4):290. 10.3390/cells8040290 PMC6523690 30934919

[398] Kim S-J Syed GH Siddiqui A. Hepatitis C Virus Induces the Mitochondrial Translocation of Parkin and Subsequent Mitophagy. Ou JJ, ed. PloS Pathog (2013) 9(3):e1003285. 10.1371/journal.ppat.1003285 23555273 PMC3610669

[399] Seto W-K Lo Y-R Pawlotsky J-M Yuen M-F. Chronic hepatitis B virus infection. Lancet (2018) 392(10161):2313–24. 10.1016/S0140-6736(18)31865-8 30496122

[400] Herrscher C Roingeard P Blanchard E. Hepatitis B Virus Entry into Cells. Cells (2020) 9(6):1486. 10.3390/cells9061486 PMC7349259 32570893

[401] Schinzari V Barnaba V Piconese S. Chronic hepatitis B virus and hepatitis C virus infections and cancer: synergy between viral and host factors. Clin Microbiol Infect (2015) 21(11):969–74. 10.1016/j.cmi.2015.06.026 26163104

[402] Seeger C Mason WS. Molecular biology of hepatitis B virus infection. Virology (2015) 479-480:672–86. 10.1016/j.virol.2015.02.031 PMC4424072 25759099

[403] Ringelhan M Heikenwalder M Protzer U. Direct Effects of Hepatitis B Virus-Encoded Proteins and Chronic Infection in Liver Cancer Development. Dig Dis (2013) 31(1):138–51. 10.1159/000347209 23797136

[404] Li J Liu Y Wang Z Liu K Wang Y Liu J. Subversion of Cellular Autophagy Machinery by Hepatitis B Virus for Viral Envelopment. J Virol (2011) 85(13):6319–33. 10.1128/JVI.02627-10 PMC3126540 21507968

[405] Tang H Da L Mao Y Li Y Li D Xu Z. Hepatitis B virus X protein sensitizes cells to starvation-induced autophagy via up-regulation of beclin 1 expression. Hepatology (2009) 49(1):60–71. 10.1002/hep.22581 19065679

[406] Bouchard MJ Schneider RJ. The Enigmatic X Gene of Hepatitis B Virus. J Virol (2004) 78(23):12725–34. 10.1128/JVI.78.23.12725-12734.2004 PMC524990 15542625

[407] Zhang H-T Chen GG Hu B-G Zhang Z-Y Yun J-P He M-L. Hepatitis B virus x protein induces autophagy via activating death-associated protein kinase. J Viral Hepat (2014) 21(9):642–9. 10.1111/jvh.12191 24188325

[408] Liu B Fang M Hu Y Huang B Li N Chang C. Hepatitis B virus X protein inhibits autophagic degradation by impairing lysosomal maturation. Autophagy (2014) 10(3):416–30. 10.4161/auto.27286 PMC4077881 24401568

[409] Zhou T Jin M Ding Y Zhang Y Sun Y Huang S. Hepatitis B virus dampens autophagy maturation via negative regulation of Rab7 expression. Biosci Trends (2016) 10(4):244–50. 10.5582/bst.2016.01049 27396843

[410] Kim S-J Khan M Quan J Till A Subramani S Siddiqui A. Hepatitis B Virus Disrupts Mitochondrial Dynamics: Induces Fission and Mitophagy to Attenuate Apoptosis. Luo G, ed. PloS Pathog (2013) 9(12):e1003722. 10.1371/journal.ppat.1003722 24339771 PMC3855539

[411] Wang X Lin Y Kemper T Chen J Yuan Z Liu S. AMPK and Akt/mTOR signalling pathways participate in glucose-mediated regulation of hepatitis B virus replication and cellular autophagy. Cell Microbiol (2020) 22(2):1–16. 10.1111/cmi.13131 31746509

[412] Chen L Ming X Li W Bi M Yan B Wang X. The microRNA-155 mediates hepatitis B virus replication by reinforcing SOCS1 signalling–induced autophagy. Cell Biochem Funct (2020) 38(4):436–42. 10.1002/cbf.3488 31930529

[413] Wu S-Y. Autophagy and microRNA in hepatitis B virus-related hepatocellular carcinoma. World J Gastroenterol (2016) 22(1):176. 10.3748/wjg.v22.i1.176 26755869 PMC4698484

[414] Ballestar E Li T. New insights into the epigenetics of inflammatory rheumatic diseases. Nat Rev Rheumatol (2017) 13(10):593–605. 10.1038/nrrheum.2017.147 28905855

[415] Liu D Li P Guo J Li L-L Guo B Jiao H-H. Exosomes derived from HBV−associated liver cancer promote chemoresistance by upregulating chaperone−mediated autophagy. Oncol Lett (2018) 17(1):323–31. 10.3892/ol.2018.9584 PMC6313222 30655770

[416] Moens U. Human Polyomaviruses and Papillomaviruses. Int J Mol Sci (2018) 19(8):2360. 10.3390/ijms19082360 PMC6122080 30103449

[417] Moens U Rasheed K Abdulsalam I Sveinbjørnsson B. The Role of Merkel Cell Polyomavirus and Other Human Polyomaviruses in Emerging Hallmarks of Cancer. Viruses (2015) 7(4):1871–901. 10.3390/v7041871 PMC4411681 25866902

[418] Liu W MacDonald M You J. Merkel cell polyomavirus infection and Merkel cell carcinoma. Curr Opin Virol (2016) 20:20–7. 10.1016/j.coviro.2016.07.011 PMC5102790 27521569

[419] Grundhoff A Fischer N. Merkel cell polyomavirus, a highly prevalent virus with tumorigenic potential. Curr Opin Virol (2015) 14:129–37. 10.1016/j.coviro.2015.08.010 26447560

[420] Wendzicki JA Moore PS Chang Y. Large T and small T antigens of Merkel cell polyomavirus. Curr Opin Virol (2015) 11:38–43. 10.1016/j.coviro.2015.01.009 25681708 PMC4456251

[421] Borchert S Czech-Sioli M Neumann F Schmidt C Wimmer P Dobner T. High-Affinity Rb Binding, p53 Inhibition, Subcellular Localization, and Transformation by Wild-Type or Tumor-Derived Shortened Merkel Cell Polyomavirus Large T Antigens. J Virol (2014) 88(6):3144–60. 10.1128/JVI.02916-13 PMC3957953 24371076

[422] Berrios C Padi M Keibler MA Park DE Molla V Cheng J. Merkel Cell Polyomavirus Small T Antigen Promotes Pro-Glycolytic Metabolic Perturbations Required for Transformation. Kalejta RF, ed. PloS Pathog (2016) 12(11):e1006020. 10.1371/journal.ppat.1006020 27880818 PMC5120958

[423] Shuda M Arora R Kwun HJ Feng H Sarid R Fernández-Figueras M-T. Human Merkel cell polyomavirus infection I. MCV T antigen expression in Merkel cell carcinoma, lymphoid tissues and lymphoid tumors. Int J Cancer (2009) 125(6):1243–9. 10.1002/ijc.24510 PMC6388400 19499546

[424] Verhaegen ME Mangelberger D Harms PW Vozheiko TD Weich JW Wilbert DM. Merkel Cell Polyomavirus Small T Antigen Is Oncogenic in Transgenic Mice. J Invest Dermatol (2015) 135(5):1415–24. 10.1038/jid.2014.446 PMC4397111 25313532

[425] Kumar S Xie H Shi H Gao J Juhlin CC Bjornhagen V. Merkel cell polyomavirus oncoproteins induce microRNAs that suppress multiple autophagy genes. Int J Cancer (2020) 146(6):1652–66. 10.1002/ijc.32503 PMC7003823 31180579

[426] Schiffman M Castle PE Jeronimo J Rodriguez AC Wacholder S. Human papillomavirus and cervical cancer. Lancet (2007) 370(9590):890–907. 10.1016/S0140-6736(07)61416-0 17826171

[427] Berman TA Schiller JT. Human papillomavirus in cervical cancer and oropharyngeal cancer: One cause, two diseases. Cancer (2017) 123(12):2219–29. 10.1002/cncr.30588 28346680

[428] DiGiuseppe S Bienkowska-Haba M Sapp M. Human Papillomavirus Entry: Hiding in a Bubble. Tsai B, ed. J Virol (2016) 90(18):8032–5. 10.1128/JVI.01065-16 PMC5008090 27412595

[429] Mattoscio D Medda A Chiocca S. Human Papilloma Virus and Autophagy. Int J Mol Sci (2018) 19(6):1775. 10.3390/ijms19061775 PMC6032050 29914057

[430] Kim YC Guan KL. MTOR: A pharmacologic target for autophagy regulation. J Clin Invest (2015) 125(1):25–32. 10.1172/JCI73939 25654547 PMC4382265

[431] Papinski D Kraft C. Regulation of Autophagy By Signaling Through the Atg1/ULK1 Complex. J Mol Biol (2016) 428(9):1725–41. 10.1016/j.jmb.2016.03.030 27059781

[432] Sapp M Bienkowska-Haba M. Viral entry mechanisms: human papillomavirus and a long journey from extracellular matrix to the nucleus. FEBS J (2009) 276(24):7206–16. 10.1111/j.1742-4658.2009.07400.x PMC2795018 19878308

[433] Pinidis P Tsikouras P Iatrakis G Zervoudis S Koukouli Z Bothou A. Human Papilloma Virus’ Life Cycle and Carcinogenesis. Maedica (Buchar) (2016) 11(1):48–54. PMC5394500 28465751

[434] Belleudi F Nanni M Raffa S Torrisi MR. HPV16 E5 deregulates the autophagic process in human keratinocytes. Oncotarget (2015) 6(11):9370–86. 10.18632/oncotarget.3326 PMC4496223 25826082

[435] Mattoscio D Casadio C Miccolo C Maffini F Raimondi A Tacchetti C. Autophagy regulates UBC9 levels during viral-mediated tumorigenesis. Raab-Traub N, ed. PloS Pathog (2017) 13(3):e1006262. 10.1371/journal.ppat.1006262 28253371 PMC5349695

[436] Dayyani F Etzel CJ Liu M Ho C-H Lippman SM Tsao AS. Meta-analysis of the impact of human papillomavirus (HPV) on cancer risk and overall survival in head and neck squamous cell carcinomas (HNSCC). Head Neck Oncol (2010) 2(1):15. 10.1186/1758-3284-2-15 20587061 PMC2908081

[437] Sannigrahi M Singh V Sharma R Panda N Khullar M. Role of autophagy in head and neck cancer and therapeutic resistance. Oral Dis (2015) 21(3):283–91. 10.1111/odi.12254 24797102

